# Design, Synthesis, Antitumor, and Antiplasmodial Evaluation of New 7-Chloroquinoline–Benzimidazole Hybrids

**DOI:** 10.3390/molecules29132997

**Published:** 2024-06-24

**Authors:** Luka Krstulović, Vesna Rastija, Lais Pessanha de Carvalho, Jana Held, Zrinka Rajić, Zorislava Živković, Miroslav Bajić, Ljubica Glavaš-Obrovac

**Affiliations:** 1 Department of Chemistry and Biochemistry, Faculty of Veterinary Medicine, University of Zagreb, Heinzelova 55, HR-10000 Zagreb, Croatia; mbajic@vef.hr; 2 Department of Agroecology and Environmental Protection, Faculty of Agrobiotechnical Sciences Osijek, Josip Juraj Strossmayer University of Osijek, Vladimira Preloga 1, HR-31000 Osijek, Croatia; vrastija@fazos.hr; 3 Institute of Tropical Medicine, University of Tuebingen, Wilhelmstrasse 27, D-72074 Tuebingen, Germany; lais_pessanha@hotmail.com (L.P.d.C.); jana.held@uni-tuebingen.de (J.H.); 4 Partner Site Tuebingen, German Center for Infection Research (DZIF),Wilhelmstrasse 27, D-72074 Tuebingen, Germany; 5 Department of Medicinal Chemistry, Faculty of Pharmacy and Biochemistry, University of Zagreb, A. Kovačića 1, HR-10000 Zagreb, Croatia; zrinka.rajic@pharma.unizg.hr; 6 General County Hospital of Našice, Bana Jelačića 10, HR-31500 Našice, Croatia; zorislava.zivkovic@obnasice.hr; 7 Department of Medicinal Chemistry, Biochemistry, and Clinical Chemistry, Faculty of Medicine Osijek, Josip Juraj Strossmayer University of Osijek, J. Huttlera 4, HR-31000 Osijek, Croatia

**Keywords:** quinoline–benzimidazole hybrids, synthesis of benzimidazoles, nucleophilic substitution, oxidative coupling of cyclocondesation, antiproliferative effect, antiplasmodial effect, QSAR

## Abstract

Newly synthesized 7-chloro-4-aminoquinoline–benzimidazole hybrids were characterized by NMR and elemental analysis. Compounds were tested for their effects on the growth of the non-tumor cell line MRC-5 (human fetal lung fibroblasts) and carcinoma (HeLa and CaCo-2), leukemia, and lymphoma (Hut78, THP-1, and HL-60) cell lines. The obtained results, expressed as the concentration at which 50% inhibition of cell growth is achieved (IC_50_ value), show that the tested compounds affect cell growth differently depending on the cell line and the applied dose (IC_50_ ranged from 0.2 to >100 µM). Also, the antiplasmodial activity of these hybrids was evaluated against two *P. falciparum* strains (*Pf*3D7 and *Pf*Dd2). The tested compounds showed potent antiplasmodial activity, against both strains, at nanomolar concentrations. Quantitative structure–activity relationship (QSAR) analysis resulted in predictive models for antiplasmodial activity against the 3D7 strain (*R*^2^ = 0.886; *R*_ext_^2^ = 0.937; *F* = 41.589) and Dd2 strain (*R*^2^ = 0.859; *R*_ext_^2^ = 0.878; *F* = 32.525) of *P. falciparum*. QSAR models identified the structural features of these favorable effects on antiplasmodial activities.

## 1. Introduction

Malaria is a life-threatening infectious disease caused by *Plasmodium* parasites. *Plasmodium falciparum* is the deadliest of the five *Plasmodium* parasite species that cause malaria in humans [1,2]. In 2022, there were 249 million cases of malaria with 608,000 estimated deaths. The African region accounts for 95% of global deaths, and it has been estimated that 80% of all deaths in this region are in children under five years of age [3]. Quinoline-based drugs such as quinine, chloroquine, and mefloquine were all used as monotherapy for malaria [4]. Unfortunately, the use of the aforementioned drugs was soon followed by reports of drug-resistant *P. falciparum* parasites [5,6,7]. Although quinoline drugs are still used against *P. vivax* [3], artemisinin-based combination therapies (ACT) are now the mainstay for the treatment of *P. falciparum* in malaria-endemic regions over the last two decades [8]. ACT therapy relies on a fast-acting artemisinin derivative that quickly reduces parasite biomass and a long-acting partner drug that kills remaining parasites and prevents recrudescence [9]. First-line ACT treatments for *P. falciparum* use quinoline-based partner drugs such piperaquine, mefloquine, and amodiaquine [3]. Recent reports of artemisinin partner drug resistance, of which reports from Africa are the most worrying, are pushing for more effective strategies and therapeutics/drug combinations [3,4,10,11,12].

Cancer and malaria have completely different pathophysiologies, and although cancer is a non-communicable disease, it was responsible for almost 10 million deaths in 2020 [13]. Combination therapy is one of the most important strategies for both cancer and malaria treatment [14,15]. Another link between the two diseases is drug resistance. Despite numerous efforts, drug resistance remains a major obstacle to the clinical application of cancer chemotherapy [16,17].

As part of our ongoing efforts to synthesize and biologically evaluate hybrid compounds [18,19,20], we report here on newly prepared hybrids containing two pharmacophores found in natural and synthetic therapeutics: quinoline and benzimidazole. Molecular hybridization is a strategy based on the fusion of two or more known pharmacophores into a single molecule [21,22,23]. The hybridization approach attempts to produce a multitarget molecule that would overcome the drawbacks of combination therapy such as drug resistance, dose limitations, and adverse drug–drug effects [24,25].

The quinoline nucleus plays a decisive role not only in the history of malaria control but also in cancer research [26,27], and it is contained in various approved molecules that act as kinase inhibitors, such as neratinib, lenvatinib, bosutinib, and cabozantinib [28,29,30,31]. Quinoline-containing antimalarials such as chloroquine and its less-toxic derivative hydroxychloroquine have been investigated in numerous clinical trials as potential anti-cancer therapeutics [32]. Benzimidazole, also a nitrogen-containing heterocycle, has been explored in medicinal chemistry for decades [33,34]. In recent years, benzimidazole compounds have gained even more attention due to their clinical application as anti-cancer therapeutics [35]. There are several examples of benzimidazole-based compounds that have been approved as anti-cancer therapeutics or have been a part of clinical trials, such as binimetinib, bendamustine, and dovitinib [36,37,38]. Due to their ability to interact with various therapeutic targets, benzimidazole compounds have also been investigated as antimalarials [39,40].

The following report describes the synthesis of 23 new 7-chloroquinoline–benzimidazole hybrids. We have investigated the biological activity of 24 hybrid compounds (including one compound previously synthesized by our group) against two *P. falciparum* strains, as well as one non-tumor and five tumor cell lines. Furthermore, we performed a quantitative structure–activity relationship (QSAR) analysis to identify the structural features of 7-chloroquinoline–benzimidazole hybrids that are important for their antiplasmodial activities and generated models for the prediction of future activities and untested analogues.

## 2. Results and Discussion

### 2.1. Chemistry

#### Design and Synthesis of New 7-Chloro-4-aminoquinoline–benzimidazoles

**Scheme 1:**
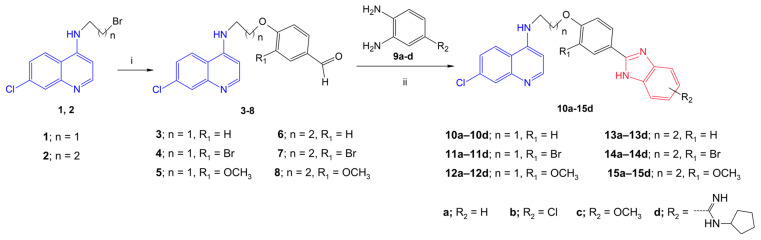
Reagents and reaction conditions: (i) appropriate 4-hydroxybenzaldehyde, K_2_CO_3_, DMF, 60 °C, 24h, (ii) Na_2_S_2_O_5_, DMSO, 165 °C, 15 min.

The synthesis of the new 7-chloro-4-aminoquinoline–benzimidazoles **10a**–**15d** was carried out as shown in Scheme 1. Synthesis, characterization, and antiproliferative activity against the HeLa and CaCo-2 cell line for compound **10d** was described in our previous report, where we found a significant difference in activity between amidine- and non-amidine benzimidazole-substituted compounds with an ethoxy linker [18]. Compound **10d** was used as the starting point for this current study, as it was a compound representative for the group that showed selective activity against leukemia cells. To further investigate the effect of non-amidine benzimidazole substituents on antiproliferative activity and to extend our studies to antiplasmodial activity, we prepared quinoline–benzimidazole hybrids with -ethyl-phenoxy- or -propyl-phenoxy- linkers between quinoline and benzimidazole, and with amidine or non-amidine substituents on benzimidazole. The compounds were further diversified by substituents at the C-2 position of the phenoxy moiety.

The quinoline-based starting compounds **1**–**8** were prepared following a previously reported procedure [18]. The benzene-1,2-diamine **9d** was prepared following previously reported procedures for a 4-(*N*-Isopropyl)amidino-1,2-phenylene diamine compound [41,42].

Quinoline benzimidazoles **10a**–**15d** were prepared by employing a condensation reaction between aldehydes **3**–**8** and benzene-1,2-diamines **4a**–**e** in the presence of sodium metabisulfite Na_2_S_2_O_5_ in DMSO at 165 °C [43].

### 2.2. Biological Activity

#### 2.2.1. Evaluation of Antiproliferative Activity of the Novel Compounds on Human Cells In Vitro

The aim of this work was to investigate the antiproliferative effect of newly synthesized 7-chloro-4-aminoquinoline–benzimidazoles with a special consideration of three structural features: (1) length of the linker (-ethyl-phenoxy or -propyl-phenoxy) between 7-chloro-4-aminoquinoline and benzimidazole, (2) introduction of a substituent (bromo or methoxy) at C-2 of the phenoxy moiety and (3) introduction of a substituent (chloro, methoxy or cyclopentylamidine (Am)) at the 5(6) position of the benzimidazole moiety.

The cell lines selected for evaluation included one non-tumor cell line, MRC-5 (human fetal lung fibroblasts), and five tumor cell lines—HeLa (human cervical adenocarcinoma), CaCo-2 (colon adenocarcinoma), Hut78 (T-cell lymphoma), THP-1 (acute monocytic leukemia), and HL-60 (acute promyelocytic leukemia). The results of the experiments, quantified by the concentration at which 50% growth inhibition (IC_50_ value) was achieved, showed different effects of the tested compounds on the growth of non-tumor and tumor cells, with IC_50_ values ranging from 0.2 to >100 µM.

As shown in Table 1, compounds **10a**–**c**, **11a**–**c**, **12a**–**c**, **13a**–**c**, **14a**–**c**, and **15a**–**c** showed strong cytotoxic effects on both non-tumor and tumor cell lines at micromolar concentrations, with IC_50_ values ranging from 0.2 to 6.1 µM. No correlation was observed between the cytotoxic effects and changes in the length of the linker between 7-chloro-4-aminoquinoline and benzimidazole, nor with the introduction of a substituent at C-2 of the phenoxy moiety or the introduction of a non-amidine substituent (chlorine or methoxy) at position 5(6) of the benzimidazole moiety.

The introduction of the cyclopentylamidine group at the 5(6) position of the benzimidazole moiety resulted in a loss in antiproliferative activity of **10d**–**15d** against the non-tumor cell line (MRC-5) and tumor lines (HeLa and CaCo-2) of epithelial origin. Compared with MRC-5 cells and carcinoma cell lines, **11d**–**15d** showed significant antiproliferative activity against lymphoma cells HuT78 (IC_50_ ranged from 15.2 to 19.7 μM) and leukemia cells (HL-60: IC_50_ ranged from 18.2 and 52.2 μM).

#### 2.2.2. Evaluation of Antiplasmodial Activity via In Vitro and SAR Analysis

**Figure 1:**
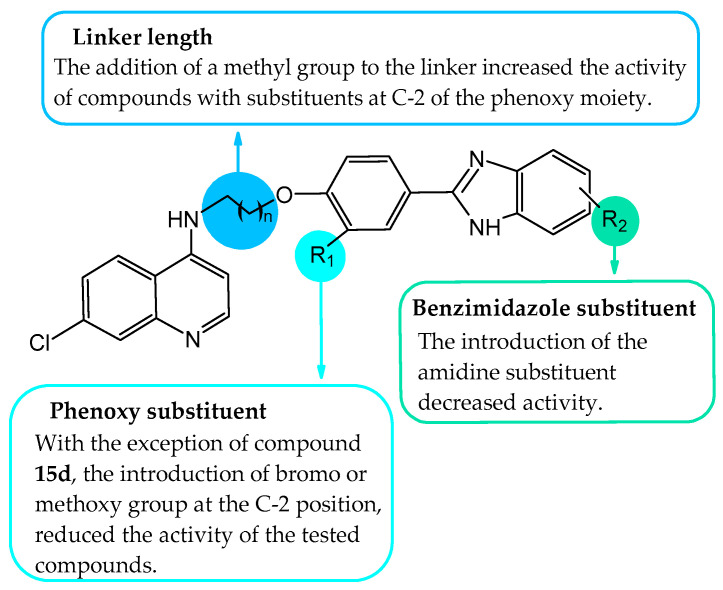
Structure activity relationship for antiplasmodial activity of the quinoline–benzimidazole hybrids.

Method used to evaluate the antiplasmodial activity of quinoline–benzimidazole hybrids against two *P. falciparum* strains, chloroquine-sensitive (*Pf*3D7) and chloroquine-resistant (*Pf*Dd2), was previously described [44,45]. As shown in Table 2, the tested compounds showed potent antiplasmodial activity, against both strains, at nanomolar concentrations. Chloroquine was used as a positive control. An insight into the observed effects of structural modifications against the two *P. falciparum* strains is shown in Figure 1.

The addition of a -CH_2_- group to the linker generally increased the activity of compounds with substituents at C-2 of the phenoxy moiety (**14a**–**15d**). Compounds **14b** (bromine at C-2) and **15d** (methoxy at C-2) showed significantly higher activity against both strains compared to their shorter chain counterparts (**11b** and **12d**).

The introduction of the bromo or methoxy group at the C-2 position of phenoxy residue generally reduced the activity of the tested compounds. The effect was most prominent for the amidine-substituted compounds **11d**, **12d**, and **14d**. Interestingly, the only exception to this trend is amidine-substituted compound **15d**. Compound **15d**, when compared to C-2 non-substituted compound **13d**, is the only compound that, after the introduction of methoxy substituent at the C-2 position, showed significantly higher activity against both strains.

The addition of benzimidazole substituents showed a similar trend to the observed anticancer activity of these compounds; the introduction of the cyclopentylamidine group decreased the activity against both strains. This effect is most pronounced for compounds **11a**–**11d** and **14a**–**14d** and least noticeable for compounds **15a**–**15d**. All non-amidine compounds showed high antiplasmodial activity in the nM range, and considering that their cytotoxicity is in the µM range (Table 1), we conclude that these compounds are selective against Plasmodium. Among the amidine-substituted compounds, **15d** is the most active hybrid (IC_50_: *Pf*3D7 = 28.7 nM, *Pf*Dd2 = 22.9 nM) and is more selective than all other non-amidine compounds (cytotoxicity is in the range of 12.8–100 µM).

### 2.3. QSAR Models for Antiplasmodial Activities

The best QSAR model obtained for the activities against the 3D7 strain of *P. falciparum* is the following:

log*Pf*3D7 = 2.553 − 1.461 *GATS7v* + 0.013 *G(N...Br)* + 0.615 *RDF135v* (1)

Five compounds (20%) (**10b**, **10c**, **14b**, **14c**, **14d**, **15a**) were selected randomly for the test set.

For activities against the Dd2 strain, the five compounds (20%) (**11c**, **14c**, 1**4d**, **15a**, **15d**) were selected by the ranking method, and the QSAR model with the best statistical qualities and strength was developed.

log*Pf*Dd2 = −5.882 + 47.216 *RBF* + 0.934 *LAI* + 0.224 *F10[C-N]* (2)

Statistical parameters of the internal and external validation of obtained QSAR models are presented in Table 3. Experimental and calculated log*Pf*3D7 and log*Pf*Dd2 by models 1 and 2, as well as values of descriptors included in models, are presented in the Supplementary Materials, Table S1 and Table S2, respectively.

The statistical parameters shown in Table 3 indicate that both models are robust and predictive and satisfy internal validation criteria. Low collinearity between them is verified by the low *Kxx* and Δ*K* ≥ 0.05 values. Both models have satisfactorily fitting performances; the coefficients of determination (*R*^2^*_tr_*) are greater than 0.60 and higher than the adjusted coefficient of determination (*R*^2^_adj_). Also, the concordance correlation coefficient of the training set (*CCC_tr_*) is higher than 0.80. To detect the reliability of the developed QSAR models, a leave-one-out (LOO) cross-validation technique was performed. The high predictive power of the models (1) and (2) was proven by great cross-validated correlation coefficients (*Q*^2^_LOO_ = 0.84 and 0.799, respectively). The proximity between the observed and predicted activity data was additionally proven by the *r^2^_m_* metric, which is higher than 0.5 [46]. The robustness of the QSAR models (1–2) was proven by the Y-randomization test. Y-scramble correlation coefficients (*R*^2^*_Yscr_*) and Y-scramble cross-validation coefficients (*Q*^2^*_Yscr_*) are lower than 0.02, which implies that the bot models were not obtained by chance [47]. The predictive power of the obtained QSAR models was proven by acceptable parameters of the external validation; the coefficients of the determination of the validation set (*R*^2^*_ext_*) are > 0.60, and an external explained variance (*Q*^2^*_F_*_1_, *Q*^2^*_F_*_2_, *Q*^2^*_F_*_3_) that are higher than 0.60. The concordance correlation coefficients of the test set (*CCC_ext_*) are higher than 0.80, which confirms the reliability of the models [48].

**Figure 2:**
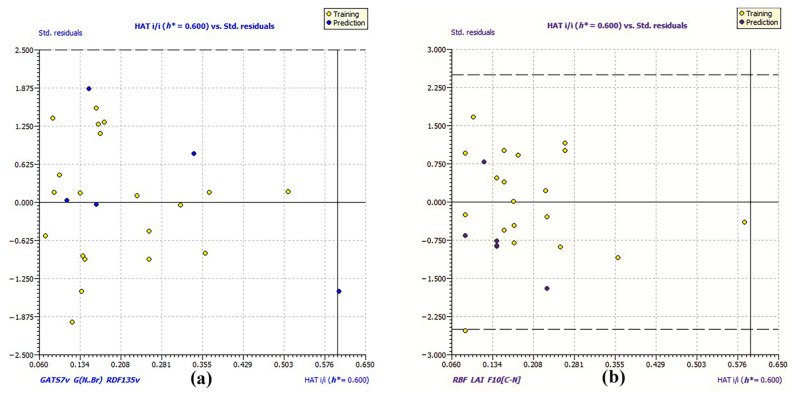
Applicability domain of QSAR models for the antiplasmodial activity against 3D7 strain (**a**) and Dd2 strain (**b**) of *P. falciparum*, represented by Williams plot. h* = warning leverage.

A Williams plot defined the chemical domain of applicability for which QSAR models can make reliable predictions [49]. A graphical representation of the applicability domain of model 1 (Williams plot) (Figure 2a) reveals molecule 14d slightly behind the warning leverage, so its predicted value must be used carefully. A Williams plot of model 2 pointed out the outlier, molecule **12a**, with a border value of standard residual (−2.519) (Figure 2b).

Molecular descriptors included in QSAR models can explain how molecular structure influences the biological mechanism of the studied compounds. The QSAR model (Equation (1)) for antiplasmodial activity against 3D7 strain contains a Geary autocorrelation index weighed by van der Waals volume, *GATS7v*. This descriptor provides information about the 3D spatial distribution of atomic van der Waals volumes [50]. According to Equation (1), enhanced values of *GATS7v* imply higher antiplasmodial activity against 3D7 strain. Autocorrelation indices have a crucial influence on the modeling and prediction of antimalarial activity. Mswahili et al. (2021) [51] observed that the majority of the selected molecular descriptors for the prediction of antimalarial activity are autocorrelation-type descriptors. Descriptor *G(N…Br)* represents the sum of the geometrical distances between the N and Br atoms. Its positive coefficient in Equation (1) implies that enhanced values of that descriptor have fewer active molecules. Molecules in the amidine group, such as **11d** and **14d**, have large values of the *G(N...Br)* descriptor and also low antiplasmodial activity, which is in accordance with SAR statements of the SAR analysis (Figure 1). Descriptor *RDF135v* is the Radial Basis Functions (RDF) descriptor, centered on the interatomic distance 13.5 Å and weighted by atomic van der Waals volume. According to the positive coefficient of *RDF135v* in the model (1), molecules with an enhanced distribution of atomic van der Waals volume at the spherical volume of radius 13.5 Å from the geometrical center of the molecule have higher values of log*Pf*3D7 or they are less active. This is especially expressed for the amidine derivatives of (**11d**, **12d**, **13d**, **14d**, **15d**). Rotatable bond fraction (*RBF*) is a constitutional descriptor presented in the model (2). It is the number of rotatable bonds divided by the total number of bonds (*nBT*) in a molecule [50]. Rotatable bonds allow for free rotation around themselves and, therefore, smooth docking into the target macromolecule (enzyme or protein) related to the studied biological activity. Therefore, an enhanced number of rotatable bonds in molecules potentially enables better biological activity. Since the amide C-N bond has a high rotational energy barrier, amide derivatives are less active against the Dd2 strain of *P. falciparum.* Descriptor *F10[C-N]* represents the frequency of the presence of C and N atoms at the topological distance 10. The highest values of these descriptors have the amidine derivatives **10d**, **11d**, and **12d**, and, consequently, these molecules are less active. The third descriptor in the model (1) is a Lipinski Alert Indeks (*LAI*), an indicator variable that takes value 1 when two or more Lipinski role properties are out of range. A Lipinski role of five can predict the chance for a molecule to become an oral drug concerning absorption and permeation through biological membranes [52].

## 3. Materials and Methods

### 3.1. Chemistry

All solvents and reagents were used without purification from commercial sources. To monitor the progress of a reaction and for comparison purposes, thin-layer chromatography (TLC) was performed on pre-coated Merck silica gel 60F-254 plates using an appropriate solvent system, and the spots were detected under UV light (254 nm). Melting points (uncorrected) were determined with a Buchi 510 melting point apparatus. ^1^H and ^13^C NMR spectra were acquired on a Bruker Avance DPX-300 or Bruker AV-600 spectrometer. All data were recorded in DMSO-d6 at 298 K. Chemical shifts were referenced to the residual solvent signal of DMSO at δ 2.50 ppm for ^1^H and δ 39.50 ppm for ^13^C. Elemental analyses for carbon, hydrogen, and nitrogen were performed on a Perkin-Elmer 2400 elemental analyzer. Analyses are indicated as symbols of elements, and the analytical results obtained were within 0.4% of the theoretical value.

Compounds **3**–**8** were prepared following the procedure reported earlier by Sanchez et al. [53]. A description of synthesis and analytical data for compounds **3** and **5** was given in the aforementioned publication and will not be shown in this paper.

#### 3.1.1. General Procedure for the Synthesis of Compounds **4** and **6**–**8**

A mixture of quinoline **1** or **2** (1 mmol), appropriate 4-hydroxybenzaldehyde (1.4 mmol), and K_2_CO_3_ (3 mmol) in DMF was heated at 60 °C for 24 h. After cooling to room temperature, the mixture was diluted with CH_2_Cl_2_ and washed three times with water. An organic layer was dried on Na_2_SO_4_ and then concentrated in a rotary evaporator. The obtained residue was dissolved in a minimal volume of MeOH; the addition of water resulted in precipitation. The product was collected by filtration.

3-bromo-4-{2-[(7-chloroquinolin-4-yl)amino]ethoxy}benzaldehyde (**4**)

Compound **4** was prepared using the above-described method from compound **1** (1 g, 3.52 mmol), 3-bromo-4-hydroxybenzaldehyde (990 mg, 4.92 mmol), and K_2_CO_3_ (1460 mg, 10.56 mmol), as white powder (885 mg, 62%); mp 198–200 °C. ^1^H NMR (300 MHz, DMSO-d6) δ/ppm 9.85 (s, 1H, CHO), 8.44 (d, J = 5.4 Hz, 1H, ArH), 8.26 (d, J = 9.0 Hz, 1H, ArH), 8.09 (d, J = 1.9 Hz, 1H, ArH), 7.91 (dd, J = 8.5 Hz, J = 1.9 Hz, 1H, ArH), 7.80 (d, J = 2.1 Hz, 1H, ArH), 7.47 (dd, J = 9.0 Hz, J = 2.1 Hz, 1H, ArH), 7.47 (s, 1H, NH), 7.37 (d, J = 8.5 Hz, 1H, ArH), 6.67 (d, J = 5.4 Hz, 1H, ArH), 4.47 (t, J = 5.4 Hz, 2H, CH_2_), 3.77 (q, J = 5.4 Hz,CH_2_). ^13^C NMR (151 MHz, DMSO-d_6_) 190.5, 159.4, 152.0, 150.0, 149.0, 135.0, 133.4, 131.1, 130.6, 127.5, 124.2, 123.94, 117.4, 113.7, 111.7, 99.2, 67.7, 41.6. Anal. calcd. for C_18_H_14_BrClN_2_O_2_ (M_r_ = 405.67): C 53.29, H 3.48, N 6.91; found: C 53.41, H 3.83, N 6.67.

4-{3-[(7-chloroquinolin-4-yl)amino]propoxy}benzaldehyde (**6**)

Compound **6** was prepared using the above-described method from compound **2** (1000 mg, 3.34 mmol), 4-hydroxybenzaldehyde (570 mg, 4.67 mmol), and K_2_CO_3_ (1380 mg, 10.02 mmol), as white powder (700 mg, 61%); mp 188–190 °C. ^1^H NMR (300 MHz, DMSO-d6) δ/ppm 9.87 (s, 1H, CHO), 8.39 (d, J = 5.4 Hz, 1H, ArH), 8.28 (d, J = 9.0 Hz, 1H, ArH), 7.87 (d, J = 8.7 Hz, 2H, ArH), 7.79 (d, J = 2.1 Hz, 1H, ArH), 7.45 (dd, J = 9.0 Hz, J = 2.1 Hz, 1H, ArH), 7.39 (t, J = 5.1 Hz, 1H, NH), 7.15 (d, J = 8.7 Hz, 2H, ArH), 6.52 (d, J = 5.4 Hz, 1H, ArH), 4.24 (t, J = 6.0 Hz, 2H, CH_2_), 3.47 (q, J = 6.4 Hz, 2H, CH_2_), 2.15 (p, J = 6.5 Hz, 2H, CH_2_). ^13^C NMR (151 MHz, DMSO-d_6_) 191.2, 163.5, 151.9, 150.0, 149.1, 133.4, 131.8, 129.6, 127.5, 124.0, 117.5, 114.9, 98.7, 65.8, 27.4. Anal. calcd. for C_19_H_17_ClN_2_O_2_ (M_r_ = 340.80): C 66.96, H 5.03, N 8.22; found: C 67.13, H 5.36, N 8.05.

3-bromo-4-{3-[(7-chloroquinolin-4-yl)amino]propoxy}benzaldehyde (**7**)

Compound **7** was prepared using the above-described method from compound **2** (1000 mg, 3.34 mmol), 3-bromo-4-hydroxybenzaldehyde (940 mg, 4.67 mmol), and K_2_CO_3_ (1380 mg, 10.02 mmol), as white powder (810 mg, 66%); mp 215–217 °C. ^1^H NMR (300 MHz,DMSO-d_6_) δ/ppm 9.86 (s, 1H, CHO), 8.39 (d, J = 5.4 Hz, 1H, ArH), 8.27 (d, J = 9.1 Hz, 1H, ArH), 8.13 (d, J = 1.9 Hz, 1H, ArH), 7.92 (dd J = 8.5, 1.9 Hz, 1H, ArH), 7.79 (d J = 1.9 Hz, 1H, ArH), 7.49–7.38 (m, 2H, ArH + NH), 7.32 (d, J = 8.5 Hz, 1H, ArH), 6.55 (d, J = 5.4 Hz, 1H, ArH), 4.32 (t, J = 5.9 Hz, 2H, CH_2_), 3.52 (q, J = 5.9 Hz, J = 6.4 Hz 2H, CH_2_), 2.17 (p, J = 6.5 Hz, 2H, CH_2_). ^13^C NMR (150 MHz, DMSO-d_6_) δ/ppm 190.5, 159.4, 151.9, 150.0, 149.1, 133.9, 133.4, 131.2, 130.5, 127.5, 124.1, 124.0, 117.5, 113.5, 111.7, 98.6, 67.0, 27.4. Anal. calcd. for C_19_H_16_BrClN_2_O_2_ (M_r_ = 419.70): C 54.37, H 3.84, N 6.67; found: C 54.28, H 3.75, N 6.44.

4-{3-[(7-chloroquinolin-4-yl)amino]propoxy}-3-methoxybenzaldehyde (**8**)

Compound **8** was prepared using the above-described method from compound **2** (1000 mg, 3.34 mmol), 3-methoxy-4-hydroxybenzaldehyde (710 mg, 4.67 mmol), and K_2_CO_3_ (1380 mg, 10.02 mmol), as white powder (935 mg, 67%); mp 185–187 °C. ^1^H NMR (300 MHz, DMSO-d_6_) δ/ppm 9.85 (s, 1H, CHO), 8.39 (d, J = 5.4 Hz, 1H, ArH), 8.27 (d, J = 9.1 Hz, 1H, ArH), 7.79 (d, J = 2.1 Hz, 1H, ArH), 7.54 (dd J = 8.2, 1.7 Hz, 1H, ArH), 7.45 (dd J = 9.0, 2.2 Hz, 1H, ArH), 7.42 (d, J = 1.7 Hz, 1H, ArH), 7.38 (t, J = 5.1 Hz, 1H, NH), 7.19 (d, J = 8.2 Hz, 1H, ArH), 6.53 (d, J = 5.4 Hz, 1H, ArH), 4.23 (t, J = 5.9 Hz, 2H, CH_2_), 3.86 (s, 3H, CH_3_), 3.52 (q, J = 5.7 Hz, J = 6.3 Hz 2H, CH_2_), 2.16 (p, J = 6.3 Hz, 2H, CH_2_). ^13^C NMR (150 MHz, DMSO-d_6_) δ/ppm 191.3, 153.4, 152.0, 150.0, 149.2, 149.1, 133.4, 129.6, 127.5, 126.0, 124.0, 124.0, 117.5, 112.1, 98.6, 66.3, 55.6, 27.5. Anal. calcd. for C_19_H_16_BrClN_2_O_2_ (M_r_ = 370.83): C 64.78, H 5.16, N 7.55; found: C 64.44, H 5.24, N 7.20.

#### 3.1.2. General procedure for the synthesis of compounds **10a**–**15d**

A solution of aldehyde **3**–**8** (1 mmol), appropriate benzene-1,2-diamine **9a**–**d** (1 mmol), and Na_2_S_2_O_5_ (0.5 mmol) in DMSO (15 mL) was heated at 165 °C for 15 min. The mixture was cooled down to room temperature. The addition of water (5 mL) resulted in precipitation. The resulting residues for compounds **10a**–**c**, **11a**–**c**, **12a**–**c**, **13a**–**c**, **14a**–**c**, and **15a**–**c** were collected with filtration, and products were obtained by recrystallization using methanol or ethanol. The resulting residues for compounds **10d**–**15d** were collected with filtration, and products were obtained by recrystallization using methanol and converted to hydrochloride salts using anhydrous methanol saturated with HCl(g).

*N*-{2-[4-(1*H*-benzo[*d*]imidazole-2-yl)phenoxy]ethyl)}-7-chloroquinolin-4-amine (**10a**)

Compound **10a** was prepared using the above-described method from compound **3** (350 mg, 1.07 mmol), diamine **9a** (115 mg, 1.07 mmol), and Na_2_S_2_O_5_ (101 mg, 0.535 mmol), as beige powder (190 mg, 43%); mp 171–173 °C. ^1^H NMR (300 MHz,DMSO-d_6_) δ/ppm 12.74 (s, 1H, NH), 8.44 (d, J = 5.5 Hz, 1H, ArH), 8.32 (d, J = 9.1 Hz, 1H, ArH), 8.11 (d, J = 8.8 Hz, 2H, ArH), 7.81 (d, J = 2.2 Hz, 1H, ArH), 7.58 (t, J = 5.5 Hz, 1H, ArH), 7.53 (brs, 1H, NH), 7.49 (dd J = 9.0, 2.2 Hz, 1H, ArH), 7.47 (d J = 2.2 Hz, 1H, ArH), 7.16 (m, 2H), 7.14 (d, J = 8.8 Hz, 2H, ArH), 6.64 (d, J = 5.5 Hz, 1H, ArH) 4.35 (t, J = 5.5 Hz, 2H, CH_2_) 3.74 (q, J = 5.5 Hz, 2H, CH_2_). ^13^C NMR (150 MHz, DMSO-d_6_) δ/ppm 159.7, 151.7, 151.2, 150.2, 148.8, 133.5, 128.0, 127.3, 124.2, 124.1, 122.8, 117.4, 114.9, 98.9, 65.7, 41.9. Anal. calcd. for C_24_H_19_ClN_4_O (M_r_ = 414.89): C 69.48, H 4.62, N 13.50; found: C 69.37, H 4.79, N 13.27.

7-chloro-*N*-{2-[4-(5(6)-chloro-1*H*-benzo[*d*]imidazole-2-yl)phenoxy]ethyl}quinolin-4-amine (**10b**)

Compound **10b** was prepared using the above-described method from compound **3** (350 mg, 1.07 mmol), diamine **9b** (152 mg, 1.07 mmol), and Na_2_S_2_O_5_ (101 mg, 0.535 mmol), as brown powder (250 mg, 52%); mp 222–224 °C. ^1^H NMR (300 MHz,DMSO-d_6_) δ/ppm 12.94 (s, 1H, NH), 8.51 (d, J = 5.8 Hz, 1H, ArH), 8.40 (d, J = 9.0 Hz, 1H, ArH), 8.21 (brs, 1H, NH), 8.10 (d, J = 8.8 Hz, 2H, ArH), 7.85 (d, J = 2.2 Hz, 1H, ArH), 7.65 (brs, 1H, ArH), 7.59 (dd, J = 9.0, 2.2 Hz, 1H, ArH), 7.52 (brs, 1H), 7.19 (d, J = 9.0 Hz, 1H, ArH), 7.14 (d, J = 9.0 Hz, 2H, ArH), 6.64 (d, J = 5.8 Hz, 1H, ArH), 4.37 (t, J = 5.3 Hz, 2H, CH_2_), 3.84 (q, J = 5.3 Hz, 2H, CH_2_). ^13^C NMR (150 MHz, DMSO-d_6_) δ/ppm 159.9, 151.9, 149.0, 145.6, 134.9, 128.2, 125.1, 124.8, 124.5, 122.4, 121.9, 116.75, 114.9, 99.0, 65.7, 41.2. Anal. calcd. for C_24_H_18_Cl_2_N_4_O × H_2_O (M_r_ = 467.35): C 61.68, H 4.31, N 11.99; found: C 61.33, H 4.40, N 12.13.

7-chloro-*N*-{2-[4-(5(6)-methoxy-1*H*-benzo[*d*]imidazole-2-yl)phenoxy]ethyl}quinolin-4-amine (**10c**)

Compound **10c** was prepared using the above-described method from compound **3** (350 mg, 1.07 mmol), diamine **9c** (148 mg, 1.07 mmol), and Na_2_S_2_O_5_ (101 mg, 0.535 mmol), as grey powder (276 mg, 58%); mp 248 °C decomp. ^1^H NMR (300 MHz,DMSO-d_6_) δ/ppm 9.46 (t, J = 5.3 Hz, 1H, NH), 8.61 (d, J = 7.0 Hz, 1H, ArH), 8.57 (d, J = 9.1 Hz, 1H, ArH), 8.06 (d, J = 8.8 Hz, 2H, ArH), 7.95 (d, J = 2.2 Hz, 1H, ArH), 7.79 (dd, J = 9.1, 2.2 Hz, 1H, ArH), 7.46 (d, J = 8.8 Hz, 1H, ArH), 7.12 (d, J = 8.8 Hz, 2H, ArH), 7.06 (d, J = 7.0 Hz, 2H, ArH), 6.83 (dd, J = 8.8, 2.2 Hz, 1H, ArH), 4.40 (t, J = 5.3 Hz, 2H, CH_2_), 4.00 (q, J = 5.3 Hz, 2H, CH_2_), 3.80 (s, CH_3_, 3H). ^13^C NMR (150 MHz, DMSO-d_6_) δ/ppm 159.5, 155.8, 155.7, 150.5, 143.3, 138.7, 138.0, 127.9, 127.0, 125.4, 122.5, 119.4, 115.4, 114.9, 111.5, 99.2, 65.7, 55.5, 42.8. Anal. calcd. for C_25_H_21_ClN_4_O_2_ (M_r_ = 444.91): C 67.49, H 4.76, N 12.59; found: C 67.18, H 4.79, N 12.87.

*N*-{2-[4-(1*H*-benzo[*d*]imidazole-2-yl)-2-bromophenoxy]ethyl}-7-chloroquinolin-4-amine (**11a**)

Compound **11a** was prepared using the above-described method from compound **4** (300 mg, 0.74 mmol), diamine **9a** (80 mg, 0.74 mmol), and Na_2_S_2_O_5_ (70 mg, 0.37 mmol), as grey powder (115 mg, 57%); mp 226–228 °C. ^1^H NMR (300 MHz,DMSO-d_6_) δ/ppm 12.85 (s, 1H, NH), 8.54 (d, J = 6.2 Hz, 1H, ArH), 8.41 (d, J = 9.1 Hz, 2H, ArH), 8.37 (d, J = 1.9 Hz, 1H, ArH), 8.16 (dd J = 8.7, 1.9 Hz, 1H, ArH), 7.87 (d, J = 1.9 Hz, 1H, ArH), 7.65 (dd J = 9.1, 1.9 Hz, 1H, ArH), 7.56 (brs, 2H), 7.37 (d J = 8.7 Hz, 1H, ArH), 7.19 (m, 2H), 6.91 (d, J = 6.2 Hz, 1H, ArH), 4.46 (t, J = 4.9 Hz, 2H, CH_2_) 3.92 (q, J = 4.9 Hz, 2H, CH_2_). ^13^C NMR (150 MHz, DMSO-d_6_) δ/ppm 155.7, 152.8, 149.7, 147.9, 135.6, 130.8, 127.2, 125.5, 124.6, 124.4, 123.7, 122.0, 116.5, 114.1, 111.4, 99.4, 67.3, 42.1. Anal. calcd. for C_24_H_18_BrClN_4_O × 0.5H_2_O (M_r_ = 502.79): C 57.33, H 3.81, N 11.14; found: C 57.27, H 3.96, N 11.21.

*N*-{[2-bromo-4-(5(6)-chloro-1*H*-benzo[*d*]imidazole-2-yl)phenoxy]ethyl}-7-chloroquinolin-4-amine (**11b**)

Compound **11b** was prepared using the above-described method from compound **4** (300 mg, 0.74 mmol), diamine **9b** (105 mg, 0.74 mmol), and Na_2_S_2_O_5_ (70 mg, 0.37 mmol), as brown powder (225 mg, 57%); mp 238–240 °C. ^1^H NMR (300 MHz,DMSO-d_6_) δ/ppm 13.04 (d, J = 9.0, 1H, NH), 8.50 (d, J = 5.8 Hz, 1H, ArH), 8.34–8.37 (m, 2H, ArH), 8.14 (dd, J = 8.7, 1.9 Hz, 1H, ArH), 8,04 (brs, 1H), 7.84 (d, J = 1.9 Hz, 1H, ArH), 7.50–7.68 (m, 3H, ArH), 7.37 (d, J = 8.7 Hz, 1H, ArH), 7.21 (brs, 1H), 6.82 (d, J = 5.8 Hz, 1H, ArH), 4.53 (t, J = 4.8 Hz, 2H, CH_2_), 3.86 (q, J = 4.8 Hz, 2H, CH_2_). ^13^C NMR (150 MHz, DMSO-d_6_) δ/ppm 156.1, 151.6, 149.5, 146.1, 135.7, 134.7, 130.9, 127.4, 125.2, 125.0, 124.3, 123.8, 118.0, 116.8, 114.1, 112.5, 111.4, 99.3, 67.4, 41.9. Anal. calcd. for C_24_H_17_BrCl_2_N_4_O × 0.5H_2_O (M_r_ = 537.24): C 53.66, H 3.38, N 10.43; found: C 53.71, H 3.70, N 10.33.

*N*-{[2-bromo-4-(5(6)-methoxy-1*H*-benzo[*d*]imidazole-2-yl)phenoxy]ethyl}-7-chloroquinolin-4-amine (**11c**)

Compound **11c** was prepared using the above-described method from compound **4** (300 mg, 0.74 mmol), diamine **9c** (102 mg, 0.74 mmol), and Na_2_S_2_O_5_ (70 mg, 0.37 mmol), as dark brown powder (190 mg, 49%); mp 208–210 °C. ^1^H NMR (300 MHz,DMSO-d_6_) δ/ppm 9.44 (s, 1H, NH), 8.65 (d, J = 7.0 Hz, 1H, ArH), 8.55 (d, J = 1.9 Hz, 1H, ArH), 8.31 (d, J = 8.8 Hz, 1H, ArH), 8.11 (dd, J = 8.8, 1.9 Hz, 1H, ArH), 7,94 (d, J = 1.9 Hz, 1H, ArH), 7.83 (dd, J = 9.0, 1.9 Hz, 1H, ArH), 7.46 (brs, 1H), 7.36 (d, J = 7.0 Hz, 1H, ArH), 7.14 (d, J = 8.8 Hz, 1H, ArH), 7.05 (brs, 1H), 6.83 (dd, J = 8.8, 1.9 Hz, 1H, ArH), 4.48 (t, J = 4.7 Hz, 2H, CH_2_), 4.07 (q, J = 4.7 Hz, 2H, CH_2_), 3.80 (s, CH_3_, 3H). ^13^C NMR (150 MHz, DMSO-d_6_) δ/ppm 155.9, 155.4, 143.2, 138.5, 138.1, 130.5, 127.0, 126.9, 125.4, 119.3, 115.4, 114.1, 111.3, 99.6, 67.3, 55.5, 42.6. Anal. calcd. for C_25_H_20_BrClN_4_O_2_ × H_2_O (M_r_ = 541.82): C 55.42, H 4.09, N 10.34; found: C 55.33, H 4.31, N 10.67.

2-{3-bromo-4-[2-(7-chloroquinolin-4-ylamino)ethoxy]phenyl}-*N*-cyclopentyl-1*H*-benzo[*d*]imidazole-5(6)-carboximidamide trihydrochloride (**11d**)

Compound **11d** was prepared using the above-described method from compound **4** (300 mg, 0.74 mmol), diamine **9d** (162 mg, 0.74 mmol), and Na_2_S_2_O_5_ (70 mg, 0.37 mmol), as brown powder (253 mg, 48%); mp 234 °C decomp. ^1^H NMR (300 MHz,DMSO-d_6_) δ/ppm 13.95 (s, 1H, NH), 9.57 (t, J = 7.5 Hz, 2H, NH), 9.36 (s, 1H, NH), 8.88 (s, 1H, NH), 8.65 (brs, 1H, ArH), 8.60 (d, J = 9.1 Hz, 1H, ArH), 8.42 (d, J = 1.8 Hz, 1H, ArH), 8.24 (dd, J = 8.7, 1.8 Hz, 1H, ArH), 8.00–7.95 (m, 2H, ArH), 7.83 (dd, J = 9.1, 1.8 Hz, 1H, ArH), 7.75 (d, J = 8.7 Hz, 1H, ArH), 7.54 (d, J = 8.7 Hz, 1H, ArH), 7.42 (d, J = 8.7 Hz, 1H, ArH), 7.16 (d, J = 7.2 Hz, 1H, ArH), 4.50 (t, J = 4.6 Hz, 2H, CH_2_), 4.14 (m, 1H, CH), 4.11–4.05 (m, 2H, CH_2_), 2.13–1.99 (m, 2H, CH_2_), 1.83–1.50 (m, 6H, CH_2_). ^13^C NMR (150 MHz, DMSO-d_6_) δ/ppm 162.8, 156.7, 156.0, 152.0, 142.9, 138.4, 138.0, 131.7, 128.3, 127.0, 125.6, 123.3, 123.0, 119.1, 115.4, 114.2, 111.5, 99.6, 67.6, 54.2, 42.4, 34.3, 31.4, 23.7. Anal. calcd. for C_30_H_28_BrClN_6_O × H_2_O × 3HCl (M_r_ = 731.34): C 49.27, H 4.55, N 11.49; found: C 48.99, H 4.87, N 11.13.

*N*-{[4-(1*H*-benzo[*d*]imidazole-2-yl)-2-methoxyphenoxy]ethyl}-7-chloroquinolin-4-amine (**12a**)

Compound **12a** was prepared using the above-described method from compound **5** (300 mg, 0.84 mmol), diamine **9a** (91 mg, 0.84 mmol), and Na_2_S_2_O_5_ (80 mg, 0.42 mmol), as grey powder (175 mg, 47%); mp 210–212 °C. ^1^H NMR (300 MHz,DMSO-d_6_) δ/ppm 12.73 (s, 1H, NH), 8.54 (d, J = 6.0 Hz, 1H, ArH), 8.49 (brs, 1H, ArH), 8.44 (d, J = 9.0 Hz, 1H, ArH), 7.87 (d, J = 2.1 Hz, 1H, ArH), 7.77 (d, J = 1.9 Hz, 1H, ArH), 7.72 (dd J = 8.4, 1.9 Hz, 1H, ArH), 7.65 (dd J = 9.0, 2.1 Hz, 1H, ArH), 7.56 (m, 2H), 7.23–7.13 (m, 3H, ArH), 6.87 (d, J = 6.0 Hz, 1H, ArH), 4.36 (t, J = 5.3 Hz, 2H, CH_2_), 3.87 (q, J = 5.3 Hz, 2H, CH_2_), 3.85 (s, 3H, CH_3_,). ^13^C NMR (150 MHz, DMSO-d_6_) δ/ppm 153.1, 151.3, 149.2, 149.1, 147.3, 143.6, 135.8, 125.6, 124.8, 123.3, 121.8, 119.2, 116.4, 113.3, 110.0, 99.3, 66.5, 55.6, 42.4. Anal. calcd. for C_25_H_21_ClN_4_O_2_ × 0.75 H_2_O (M_r_ = 458.42): C 65.50, H 4.95, N 12.22; found: C 65.53, H 5.23, N 12.56.

7-chloro-*N*-{[4-(5(6)-chloro-benzo[*d*]imidazole-2-yl)-2-methoxyphenoxy]ethyl}quinolin-4-amine (**12b**)

Compound **12b** was prepared using the above-described method from compound **5** (300 mg, 0.84 mmol), diamine **9b** (120 mg, 0.84 mmol), and Na_2_S_2_O_5_ (80 mg, 0.42 mmol), as brown powder (225 mg, 56%); mp 222 °C. ^1^H NMR (300 MHz,DMSO-d_6_) δ/ppm 12.93 (s, 1H, NH), 8.52 (d, J = 6.2 Hz, 1H, ArH), 8.45 (brs, 1h, ArH), 8.43 (d, J = 9.0 Hz, 1H, ArH), 7.86 (d, J = 1.9 Hz, 1H, ArH), 7.75 (brs, 1H, NH), 7.72 (d, J = 9.0 Hz, 1H, ArH), 7.63 (dd, J = 9.0, 1.9 Hz, 1H, ArH), 7.60–7.52 (m, 2H, ArH), 7.19 (d, J = 8.5 Hz, 2H, ArH), 6.84 (d, J = 6.2 Hz, 1H, ArH), 4.36 (t, J = 4.9 Hz, 2H, CH_2_), 3.86 (q, J = 4.9 Hz, 2H, CH_2_), 3.84 (s, 3H, CH_3_). ^13^C NMR (150 MHz, DMSO-d_6_) δ/ppm 153.0, 149.5, 149.1, 147.4, 135.8, 125.6, 124.8, 123.3, 122.7, 122.0, 119.4, 116.4, 113.3, 110.1, 99.3, 66.4, 55.6, 42.4. Anal. calcd. for C_25_H_20_Cl_2_N_4_O_2_ × H_2_O (M_r_ = 497.4): C 59.30, H 4.58, N 11.06; found: C 59.63 H 4.89, N 11.43.

7-chloro-*N*-{[4-(5(6)-methoxy-benzo[*d*]imidazole-2-yl)-2-methoxyphenoxy]ethyl}quinolin-4-amine (**12c**)

Compound **12c** was prepared using the above-described method from compound **5** (300 mg, 0.84 mmol), diamine **9c** (116 mg, 0.84 mmol), and Na_2_S_2_O_5_ (80 mg, 0.42 mmol), as dark brown powder (190 mg, 49%); mp 218–219 °C. ^1^H NMR (300 MHz,DMSO-d_6_) δ/ppm 9.48 (t, J = 5.0 Hz, 1H, NH), 8.64 (d, J = 7.1 Hz, 1H, ArH), 8.58 (d, J = 9.1 Hz, 1H, ArH), 7.95 (d, J = 1.9 Hz, 1H, ArH), 7.83 (dd, J = 9.1, 1.9 Hz, 1H, ArH), 7.73 (s, 1H, ArH), 7.69 (dd, J = 8.3, 1.9 Hz, 1H, ArH), 7.48 (d, J = 8.9 Hz, 1H, ArH), 7.19 (d, J = 8.3 Hz, 1H, ArH), 7.10 (d, J = 7.1 Hz, 1H, ArH), 7.07 (brs, 1H), 6.85 (dd, J = 8.9, 1.9 Hz, 1H, ArH), 4.39 (t, J = 5.0 Hz, 2H, CH_2_), 4.00 (q, J = 5.0 Hz, 2H, CH_2_), 3.82 (s, 3H, CH_3_), 3.81 (s, 3H, CH_3_). ^13^C NMR (150 MHz, DMSO-d_6_) δ/ppm 156.2, 155.9, 150.19, 149.5, 149.1, 142.9, 138.5, 138.0, 127.0, 125.5, 119.5, 119.2, 115.4, 113.4, 112.1, 110.1, 99.4, 66.6, 55.7, 55.5, 42.8. Anal. calcd. for C_26_H_23_ClN_4_O_3_ × H_2_O (M_r_ = 492.95): C 63.35, H 5.11, N 11.37; found: C 63.63, H 5.42, N 11.48.

2-{3-methoxy-4-[2-(7-chloroquinolin-4-ylamino)ethoxy]phenyl}-*N*-cyclopentyl-1*H*-benzo[*d*]imidazole-5(6)-carboximidamide trihydrochloride (**12d**)

Compound **12d** was prepared using the above-described method from compound **5** (300 mg, 0.84 mmol), diamine **9d** (183 mg, 0.84 mmol), and Na_2_S_2_O_5_ (80 mg, 0.42 mmol), as dark green powder (235 mg, 40%); mp 226 °C decomp. ^1^H NMR (300 MHz,DMSO-d_6_) δ/ppm 14.31 (s, 1H, NH), 9.73 (s, 1H, NH), 9.66 (s, 1H, NH), 9.44 (s, 1H, NH), 8.97 (s, 1H, NH), 8.69 (s, 1H, ArH), 8.64 (s, 1H, ArH), 8.20–7.85 (m, 4H, ArH), 7.80 (s, 2H, ArH), 7.59 (s, 1H, ArH), 7.26 (s, 1H, ArH), 7.09 (s, 1H, ArH), 4.43 (s, 2H, CH_2_), 4.17 (s, 1H, CH), 4.03 (m, 2H, CH_2_), 3.86 (s, 3H, CH_3_), 2.07 (s, 2H, CH_2_), 1.74 (s, 4H, CH_2_), 1.61 (s, 2H, CH_2_). ^13^C NMR (150 MHz, DMSO-d_6_) δ/ppm 163.1, 155.9, 149.7, 149.0, 142.8, 138.5, 137.9, 126.9, 125.8, 122.4, 122.2, 119.1, 115.4. 113.4, 110.5, 99.5, 66.7, 55.9, 54.2, 42.8, 31.5, 23.7. Anal. calcd. for C_31_H_31_ClN_6_O_2_ × 0.5H_2_O × 3HCl (M_r_ = 673.46): C 55.29, H 5.24, N 12.48; found: C 55.47, H 5.63, N 12.49.

*N*-{2-[4-(1*H*-benzo[*d*]imidazole-2-yl)phenoxy]propyl)}-7-chloroquinolin-4-amine (**13a**)

Compound **13a** was prepared using the above-described method from compound **6** (350 mg, 1.03 mmol), diamine **9a** (112 mg, 1.03 mmol), and Na_2_S_2_O_5_ (98 mg, 0.515 mmol), as pale yellow powder (205 mg, 46%); mp 200–202 °C. ^1^H NMR (300 MHz,DMSO-d_6_) δ/ppm 8.48 (d, J = 6.1 Hz, 1H, ArH), 8.42 (d, J = 8.9 Hz, 2H, ArH), 8.11 (d, J = 8.9 Hz, 2H, ArH), 7.86 (d, J = 1.6 Hz, 1H, ArH), 7.63 (dd J = 8.9, 1.5 Hz, 1H, ArH), 7.58–7.51 (m, 2H), 7.21–7.14 (m, 2H, ArH), 7.11 (d, J = 8.9 Hz, 2H, ArH), 6.75 (d, J = 6.1 Hz, 1H, ArH), 4.21 (t, J = 5.5 Hz, 2H, CH_2_) 3.61 (q, J = 5.5 Hz, 2H, CH_2_), 2.18 (p, J = 5.5 Hz, 2H). ^13^C NMR (150 MHz, DMSO-d_6_) δ/ppm 159.8, 152.8, 151.3, 147.4, 143.7, 135.7, 128.0, 125.5, 124.8, 123.3, 122.7, 121.7, 116.4, 114.8, 98.6, 65.3, 27.5. Anal. calcd. for C_25_H_21_ClN_4_O × 0.5H_2_O (M_r_ = 437.92): C 68.57, H 5.06, N 12.79; found: C 68.36, H 5.11, N 13.17.

7-chloro-*N*-{2-[4-(5(6)-chloro-1*H*-benzo[*d*]imidazole-2-yl)phenoxy]propyl}quinolin-4-amine (**13b**)

Compound **13b** was prepared using the above-described method from compound **6** (350 mg, 1.03 mmol), diamine **9b** (152 mg, 1.03 mmol), and Na_2_S_2_O_5_ (98 mg, 0.515 mmol), as light brown powder (200 mg, 42%); mp 198–200 °C. ^1^H NMR (300 MHz,DMSO-d_6_) δ/ppm 12.93 (s, 1H, NH), 8.46 (d, J = 6.0 Hz, 1H, ArH), 8.40 (d, J = 9.0 Hz, 1H, ArH), 8.26 (brs, 1H, NH), 8.10 (d, J = 8.6 Hz, 2H, ArH), 7.85 (d, J = 1.6 Hz, 1H, ArH), 7.60 (dd, J = 9.0, 1.6 Hz, 1H, ArH), 7.54 (brs, 2H, ArH), 7.19 (dd, J = 8.6, 1.6 Hz, 1H, ArH), 7.12 (d, J = 8.6 Hz, 2H, ArH), 6.71 (d, J = 6.0 Hz, 1H, ArH), 4.21 (t, J = 5.6 Hz, 2H, CH_2_), 3.59 (q, J = 5.6 Hz, 2H, CH_2_), 2.17 (p, J = 5.6 Hz, 2H, CH_2_). ^13^C NMR (150 MHz, DMSO-d_6_) δ/ppm 160.1, 152.7, 152.0, 148.7, 145.2, 135.1, 131.8, 128.2, 126.1, 125.1, 124.6, 124.4, 122.2, 121.9, 116.7, 114.9, 98.6, 65.4, 27.5. Anal. calcd. for C_25_H_20_Cl_2_N_4_O × H_2_O (M_r_ = 481.37): C 62.38, H 4.61, N 11.64; found: C 62.06, H 4.94, N 11.90.

7-chloro-*N*-{2-[4-(5(6)-methoxy-1*H*-benzo[*d*]midazole-2-yl)phenoxy]propyl}quinolin-4-amine (**13c**)

Compound **13c** was prepared using the above-described method from compound **6** (350 mg, 1.03 mmol), diamine **9c** (142 mg, 1.03 mmol), and Na_2_S_2_O_5_ (98 mg, 0.515 mmol), as brown powder (245 mg, 53%); mp 212 °C decomp. ^1^H NMR (300 MHz,DMSO-d_6_) δ/ppm 13.11 (brs, 1H, NH), 9.37 (t, J = 4.8 Hz, 1H, NH), 8.57 (d, J = 3.1 Hz, 1H, ArH), 8.54 (d, J = 5.4 Hz, 1H, ArH), 8.06 (d, J = 8.8 Hz, 2H, ArH), 7.93 (d, J = 1.9 Hz, 1H, ArH), 7.80 (dd, J = 9.0, 1.9 Hz, 1H, ArH), 7.46 (d, J = 8.8 Hz, 1H, ArH), 7.10 (d, J = 8.8 Hz, 2H, ArH), 7.06 (d, J = 1.9 Hz, 1H, ArH), 6.96 (d, J = 7.1 Hz, 1H, ArH), 6.83 (dd, J = 8.8, 1.9 Hz, 1H, ArH), 4.22 (t, J = 5.6 Hz, 2H, CH_2_), 3.80 (s, 3H, CH_3_) 3.74 (q, J = 5.6 Hz, 2H, CH_2_), 2.19 (p, J = 5.6 Hz, 2H, CH_2_). ^13^C NMR (150 MHz, DMSO-d_6_) δ/ppm 152.8, 149.5, 149.1, 147.7, 144.1, 135.6, 125.5, 124.7, 123.6, 122.7, 119.4, 116.5, 113.3, 110.1, 99.2, 66.4, 55.6, 42.4. Anal. calcd. for C_26_H_23_ClN_4_O_2_ × H_2_O (M_r_ = 476.95): C 65.47, H 5.28, N 11.75; found: C 65.09, H 5.58, N 11.49.

2-{4-[2-(7-chloroquinolin-4-ylamino)propoxy]phenyl}-*N*-cyclopentyl-1*H*-benzo[*d*]imidazole-5(6)-carboximidamide trihydrochloride (**13d**)

Compound **13d** was prepared using the above-described method from compound **6** (350 mg, 1.03 mmol), diamine **9d** (224 mg, 1.03 mmol), and Na_2_S_2_O_5_ (98 mg, 0.515 mmol), as brown powder (345 mg, 50%); mp > 275 °C. ^1^H NMR (DMSO-d_6_) δ/ppm: 14.43 (s, 1H, NH), 9.81 (d, J = 6.7 Hz, 1H, NH), 9.75 (t, J = 5.6 Hz, 1H, NH), 9.58 (s, 1H, NH), 9.12 (s, 1H, NH), 8.74 (d, J = 9.1 Hz, 1H, ArH), 8.55 (brs, 1H, ArH), 8.44 (d, J = 8.6 Hz, 2H, ArH), 8.13–8.05 (m, 2H, ArH), 7.88 (d, J = 8.6 Hz, 1H, ArH), 7.77 (dd, J = 9.1, 1.9 Hz, 1H, ArH), 7.71 (d, J = 8.6, 1H, ArH), 7.24 (d, J = 8.6 Hz, 2H, ArH), 6.94 (d, J = 7.2 Hz, 1H, ArH), 4.28 (t, J = 5.6 Hz, 2H, CH_2_), 4.23–4.10 (m, 1H, CH), 3.75 (q, J = 5.6 Hz, 2H, CH_2_), 2.19 (p, J = 5.6 Hz, 2H, CH_2_), 2.14–2.00 (m, 2H, CH_2_), 1.84–1.50 (m, 6H, CH_2_). ^13^C NMR (DMSO-d_6_) δ/ppm: 162.4, 162.1, 155.4, 151.7, 142.7, 138.5, 137.8, 130.2, 126.7, 126.0, 124.9, 124.7, 118.9, 115.5, 115.4, 114.9, 114.0, 98.5, 65.7, 54.9, 54.4, 31.4, 27.3, 23.7. Anal. calcd. for C_31_H_31_ClN_6_O × 3HCl × H_2_O (M_r_ = 666.47): C 55.87, H 5.44, N 12.61; found: C 55.59, H 5.21, N 13.04.

*N*-{2-[4-(1*H*-benzo[*d*]imidazole-2-yl)-2-bromophenoxy]propyl}-7-chloroquinolin-4-amine (**14a**)

Compound **14a** was prepared using the above-described method from compound **7** (350 mg, 0.84 mmol), diamine **9a** (90 mg, 0.84 mmol), and Na_2_S_2_O_5_ (80 mg, 0.42 mmol), as grey powder (255 mg, 60%); mp 173–175 °C. ^1^H NMR (300 MHz,DMSO-d_6_) δ/ppm 12.83 (s, 1H, NH), 8.48 (d, J = 6.2 Hz, 1H, ArH), 8.40 (s, 1H, ArH), 8.39 (d, J = 8.7 Hz, 1H, ArH), 8.25 (t, J = 5.0 Hz, 1H, NH), 8.16 (dd J = 8.5, 1.9 Hz, 1H, ArH), 7.84 (d, J = 1.9 Hz, 1H, ArH), 7.60 (dd J = 9.0, 1.9 Hz, 1H, ArH), 7.56 (brs, 2H,), 7.31 (d J = 8.7 Hz, 1H, ArH), 7.19 (m, 2H), 6.73 (d, J = 6.2 Hz, 1H, ArH), 4.30 (t, J = 5.0 Hz, 2H, CH_2_) 3.63 (q, J = 5.0 Hz, 2H, CH_2_), 2.20 (p, J = 5.0 Hz, 2H, CH_2_). ^13^C NMR (150 MHz, DMSO-d_6_) δ/ppm 155.8, 152.1, 149.8, 148.5, 145.0, 135.2, 130.7, 127.3, 125.1, 124.7, 124.2, 124.1, 122.0, 122.0, 116.7, 113.8, 111.4, 98.6, 66.5, 27.4. Anal. calcd. for C_25_H_20_BrClN_4_O × 1.5H_2_O (M_r_ = 534.83): C 56.14, H 4.33, N 10.48; found: C 54.51, H 4.67, N 10.23.

*N*-{[2-bromo-4-(5(6)-chloro-1*H*-benzo[*d*]imidazole-2-yl)phenoxy]propyl}-7-chloroquinolin-4-amine (**14b**)

Compound **14b** was prepared using the above-described method from compound **7** (350 mg, 0.84 mmol), diamine **9b** (120 mg, 0.84 mmol), and Na_2_S_2_O_5_ (80 mg, 0.42 mmol), as brown powder (215 mg, 47%); mp 219–221 °C. ^1^H NMR (300 MHz,DMSO-d_6_) δ/ppm 13.06 (s, 1H, NH), 8.47 (d, J = 6.0 Hz, 1H, ArH), 8.40 (s, 1H, ArH), 8.38 (d, J = 6.3 Hz, 1H, ArH), 8.24 (t, J = 5.3 Hz, 1H, NH), 8.15 (dd, J = 8.6, 1.8 Hz, 1H, ArH), 7.84 (d, J = 1.8 Hz, 1H, ArH), 7.71–7.48 (m, 3H, ArH), 7.32 (d, J = 8.6 Hz, 1H, ArH), 7.21 (dd, J = 8.6 Hz, 1.8, 1H, ArH), 6.72 (d, J = 6.0 Hz, 1H, ArH), 4.30 (t, J = 5.3 Hz, 2H, CH_2_), 3.63 (q, J = 5.3 Hz, 2H, CH_2_), 2.18 (p, J = 5.3 Hz, 2H, CH_2_). ^13^C NMR (150 MHz, DMSO-d_6_) δ/ppm 156.1, 152.2, 151.3, 148.4, 144.9, 135.2, 130.9, 127.5, 125.1, 124.7, 124.2, 123.6, 122.3, 116.7, 113.8, 111.4, 98.5, 66.5, 27.4. Anal. calcd. for C_25_H_19_BrCl_2_N_4_O × 2H_2_O (M_r_ = 578.29): C 51.92, H 4.01, N 9.69; found: C 52.14, H 3.86, N 10.03.

*N*-{[2-bromo-4-(5(6)-methoxy-1*H*-benzo[*d*]imidazole-2-yl)phenoxy]propyl}-7-chloroquinolin-4-amine (**14c**)

Compound **14c** was prepared using the above-described method from compound **7** (350 mg, 0.84 mmol), diamine **9c** (116 mg, 0.84 mmol), and Na_2_S_2_O_5_ (80 mg, 0.42 mmol), as dark brown powder (221 mg, 49%); mp 230 °C decomp. ^1^H NMR (300 MHz,DMSO-d_6_) δ/ppm 12,55 (brs, 1H, NH), 9.40 (t, J = 5.4, 1H, NH), 8.58 (d, J = 7.0 Hz, 1H, ArH), 8.54 (d, J = 9.1 Hz, 1H, ArH), 8.34 (d, J = 1.8 Hz, 1H, ArH), 8.11 (dd, J = 8.7, 1.8 Hz, 1H, ArH), 7,93 (d, J = 1.8 Hz, 1H, ArH), 7.78 (dd, J = 9.1, 1.9 Hz, 1H, ArH), 7.46 (d, J = 8.7 Hz, 1H, ArH), 7.29 (d, J = 8.7 Hz, 1H, ArH), 7.06 (d, J = 1.5 Hz, 1H, ArH), 6.95 (d, J = 7.0 Hz, 1H, ArH), 6.83 (dd, J = 8.7, 2.3 Hz, 1H, ArH), 4.30 (t, J = 5.4 Hz, 2H, CH_2_), 3.80 (s, 3H, CH_3_), 3.77 (q, J = 5.4 Hz, 2H, CH_2_), 2. (p, J = 5.4 Hz, 2H, CH_2_). ^13^C NMR (150 MHz, DMSO-d_6_) δ/ppm 155.8, 155.5, 155.3, 149.3, 143.5, 138.8, 137.9, 130.4, 127.0, 126.8, 125.5, 124.2, 119.4, 115.6, 113.8, 111.6, 111.4, 98.5, 66.3, 55.4, 27.3. Anal. calcd. for C_26_H_22_BrClN_4_O_2_ × 0.5H_2_O (M_r_ = 546.84): C 57.11, H 4.24, N 10.25; found: C 56.88, H 4.27, N 10.41.

2-{3-bromo-4-[2-(7-chloroquinolin-4-ylamino)propoxy]phenyl}-*N*-cyclopentyl-1*H*-benzo[*d*]imidazole-5(6)-carboximidamide trihydrochloride (**14d**)

Compound **14d** was prepared using the above-described method from compound **7** (350 mg, 0.84 mmol), diamine **9d** (183 mg, 0.84 mmol), and Na_2_S_2_O_5_ (80 mg, 0.42 mmol), as grey powder (225 mg, 50%); mp 265 °C decomp. ^1^H NMR (300 MHz,DMSO-d_6_) δ/ppm 14.27 (d, J = 12.1 Hz, 1H, NH), 9.69 (s, 2H, NH), 9.46 (s, 1H, NH), 8.99 (s, 1H, NH), 8.68 (dd, J = 8.9, 3.3 Hz, 1H, ArH), 8.59 (m, 2H, ArH), 8.36 (m, 1H, ArH), 8.05 (d, J = 9.0 Hz, 2H, ArH), 7.80 (m, 2H, ArH), 7.60 (d, J = 8.5 Hz, 1H, ArH), 7.38 (d, J = 8.5 Hz, 1H, ArH), 6.95 (d, J = 7.0 Hz, 1H, ArH), 4.35 (t, J = 5.0 Hz, 2H, CH_2_), 4.22–4.11 (m, 1H, CH), 3.78 (m, 2H, CH_2_), 2.08 (m, 2H, CH_2_), 1.84–1.53 (m, 6H, CH_2_). ^13^C NMR (150 MHz, DMSO-d_6_) δ/ppm 162.5, 157.5, 155.4, 151.1, 142.7, 138.5, 137.8, 132.2, 129.1, 126.7, 126.1, 124.3, 124.1, 118.9, 115.6, 114.0, 111.7, 98.4, 66.8, 54.3, 31.4, 27.3, 23.7. Anal. calcd. for C_31_H_30_BrClN_6_O × 1.75H_2_O × 3HCl (M_r_ = 758.88): C 49.06, H 4.85, N 11.07; found: C 48.73, H 5.12, N 10.79.

*N*-{[4-(1*H*-benzo[*d*]imidazole-2-yl)-2-methoxyphenoxy]propyl}-7-chloroquinolin-4-amine (**15a**)

Compound **15a** was prepared using the above-described method from compound **8** (350 mg, 0.95 mmol), diamine **9a** (103 mg, 0.95 mmol), and Na_2_S_2_O_5_ (90 mg, 0.47 mmol), as grey powder (325 mg, 54%); mp 179–181 °C. ^1^H NMR (300 MHz,DMSO-d_6_) δ/ppm 8.45 (d, J = 6.0 Hz, 1H, ArH), 8.37 (d, J = 9.0 Hz, 1H, ArH), 8.06 (brs, 1H, NH), 7.83 (d, J = 2.0 Hz, 1H, ArH), 7.79 (d, J = 1.5 Hz, 1H, ArH), 7.73 (dd J = 8.4, 1.5 Hz, 1H, ArH), 7.63–7.51 (m, 3H, ArH), 7.23–7.09 (m, 3H, ArH), 6.68 (d, J = 6.0 Hz, 1H, ArH), 4.20 (t, J = 5.7 Hz, 2H, CH_2_), 3.89 (s, 3H, CH_3_), 3.57 (q, J = 5.7 Hz, 2H, CH_2_), 2.17 (p, J = 5.7 Hz, 2H, CH_2_). ^13^C NMR (150 MHz, DMSO-d_6_) δ/ppm 151.9, 151.4, 149.4, 149.0, 148.9, 145.5, 134.9, 125.0, 124.7, 124.5, 122.9, 121.8, 119.2, 116.8, 112.9, 109.9, 98.6, 66.0, 55.6, 27.6. Anal. calcd. for C_26_H_23_ClN_4_O_2_ (M_r_ = 458.94): C 68.04, H 5.05, N 12.21; found: C 67.77, H 5.13, N 12.09.

7-chloro-*N*-{[4-(5(6)-chloro-benzo[*d*]imidazole-2-yl)-2-methoxyphenoxy]propyl}quinolin-4-amine (**15b**)

Compound **15b** was prepared using the above-described method from compound **8** (350 mg, 0.95 mmol), diamine **9b** (135 mg, 0.95 mmol), and Na_2_S_2_O_5_ (90 mg, 0.47 mmol), as brown powder (253 mg, 54%); mp 190–192 °C. ^1^H NMR (300 MHz,DMSO-d_6_) δ/ppm 12.95 (s, 1H, NH), 8.45 (d, J = 5.9 Hz, 1H, ArH), 8.37 (d, J = 9.0 Hz, 1H, ArH), 8.08 (brs, 1H, NH), 7.83 (d, J = 1.9 Hz, 1H, ArH), 7.72 (d, J = 8.5 Hz, 1H, ArH), 7.72 (d, J = 8.5 Hz, 1H, ArH), 7.68–7.43 (m, 3H, ArH), 7.20 (dd, J = 8.5, 1.6 Hz, 1H, ArH), 7.15 (d, J = 8.5 Hz, 1H, ArH), 6.69 (d, J = 5.0 Hz, 1H, ArH), 4.20 (t, J = 5.6 Hz, 2H, 3.89 (s, 3H, CH_3_), CH_2_), 3.57 (q, J = 5.6 Hz, 2H, CH_2_), 2.17 (p, J = 5.6 Hz, 2H, CH_2_). ^13^C NMR (150 MHz, DMSO-d_6_) δ/ppm 152.0, 149.7, 149.0, 148.7, 145.2, 135.0, 125.1, 124.6, 124.5, 122.32, 119.5, 116.7, 112.9, 109.9, 98.6, 66.0, 55.64, 27.5. Anal. calcd. for C_26_H_22_Cl_2_N_4_O_2_ × 1.5H_2_O (M_r_ = 520.41): C 60.01, H 4.84, N 10.77; found: C 60.18, H 5.07, N 11.06.

7-chloro-*N*-{[4-(5(6)-methoxy-benzo[*d*]imidazole-2-yl)-2-methoxyphenoxy]propyl}quinolin-4-amine (**15c**)

Compound **15c** was prepared using the above-described method from compound **8** (350 mg, 0.95 mmol), diamine **9c** (130 mg, 0.95 mmol), and Na_2_S_2_O_5_ (90 mg, 0.47 mmol), as brown powder (246 mg, 53%); mp 220 °C decomp. ^1^H NMR (300 MHz,DMSO-d_6_) δ/ppm 12.96 (brs, 1H, NH), 9.38 (brs, 1H, NH), 8.55 (brs, 1H, ArH), 8.53 (d, J = 4.3 Hz, 1H, ArH), 7.93 (d, J = 1.3 Hz, 1H, ArH), 7.80 (d, J = 9.0 Hz, 1H, ArH), 7.73 (s, 1H, ArH), 7.69 (d, J = 8.4 Hz, 1H, ArH), 7.48 (d, J = 8.7 Hz, 1H, ArH), 7.14 (d, J = 8.4 Hz, 1H, ArH), 7.07 (s, 1H, ArH), 6.95 (d, J = 7.0 Hz, 1H, ArH), 6.84 (d, J = 8.7, 1.8 Hz, 1H, ArH), 4.20 (t, J = 5.1 Hz, 2H, CH_2_), 3.83 (s, CH_3_, 3H), 3.80 (s, 3H, CH_3_), 3.74 (q, J = 5.1 Hz, 2H, CH_2_), 2.19 (p, J = 5.1 Hz, 2H, CH_2_). ^13^C NMR (150 MHz, DMSO-d_6_) δ/ppm 155.8, 155.4, 150.7, 149.3, 149.0, 143.2, 138.6, 137.9, 126.8, 125.5, 122.4, 119.2, 119.1, 115.5, 113.0, 111.5, 109.7, 98.6, 65.8, 55.6, 55.5, 27.4. Anal. calcd. for C_27_H_25_ClN_4_O_3_ × 0.5H_2_O (M_r_ = 497.97): C 65.12, H 5.26, N 11.25; found: C 64.92, H 5.52, N 11.59.

2-{3-methoxy-4-[2-(7-chloroquinolin-4-ylamino)propoxy]phenyl}-*N*-cyclopentyl-1*H*-benzo[*d*]imidazole-5(6)-carboximidamide trihydrochloride (**15d**)

Compound **15d** was prepared using the above-described method from compound **8** (350 mg, 0.95 mmol), diamine **9d** (207 mg, 0.95 mmol), and Na_2_S_2_O_5_ (90 mg, 0.47 mmol), as grey powder (245 mg, 36%); mp 260 °C decomp. ^1^H NMR (300 MHz,DMSO-d_6_) δ/ppm 14.25 (s, 1H, NH), 9.76 (d, J = 6.8 Hz, 1H, NH), 9.64 (t, J = 5.2 Hz, 1H, NH), 9.53 (s, 1H, NH), 9.05 (s, 1H, NH), 8.66 (d, J = 9.1 Hz, 1H, ArH), 8.55 (brs, 1H, ArH), 8.11 (brs, 1H, ArH), 8.09–7.98 (m, 3H, ArH), 7.86 (d, J = 8.3 Hz, 1H, ArH), 7.78 (dd, J = 8.9, 1.3 Hz, 1H, ArH), 7.67 (s, J = 8.1 Hz, 1H, ArH), 7.25 (d, J = 8.5 Hz, 1H, ArH), 6.94 (d, J = 7.0 Hz, 1H, ArH), 4.26 (t, J = 4.8 Hz, 2H, CH_2_), 4.21–4.14 (m, 1H, CH), 3.87 (s, 3H, CH_3_), 3.74 (q, J = 4.8 Hz, 2H, CH_2_ 2H, CH_2_), 2.21 (p, J = 4.8 Hz, 2H, CH_2_), 2.08 (m, 2H, CH_2_), 1.84–1.50 (s, 6H, CH_2_). ^13^C NMR (150 MHz, DMSO-d_6_) δ/ppm 162.5, 155.4, 149.2, 142.6, 138.5, 137.8, 126.7, 126.0, 119.0, 115.5, 113.0, 111.3, 98.4, 66.2, 56.2, 54.4, 31.4, 27.3, 23.7. Anal. calcd. for C_32_H_33_ClN_6_O_2_ × H_2_O × 3HCl (M_r_ = 696.49.5): C 55.18, H 5.50, N 12.07; found: C 54.81, H 5.19, N 11.78.

### 3.2. Biological Activity

#### 3.2.1. Evaluation of the Antiproliferative Activity of the Novel Compounds on Human Cells

##### Cell Lines and Cell Culturing

The effect of the new synthesized compounds was tested on five human tumor cell lines, HeLa (human cervical adenocarcinoma; from ATCC), CaCo-2 (human colorectal adenocarcinoma), HL-60 (acute promyelocytic leukemia), HuT78 (T-cell lymphoma), and THP-1 (acute monocytic leukemia), as well as one non-tumor cell line, MRC-5 (human fetal lung fibroblasts). The MRC-5 cells were used between 24 and 26 passages. The cells were cultured in two different types of media: DMEM (Gibco Thermo Fisher Scinetific Inc., Runcorn, UK) and RPMI 1640 (Gibco, Thermo Fisher Scinetific Inc., UK). Both media were supplemented with 2 mM glutamine, fetal bovine serum (10%; heat inactivated), and antibiotics (100 U penicillin and 0.1 mg streptomycin). RPMI 1640 was additionally supplemented with 10 mM HEPES and 1 mM sodium pyruvate. Cells growing in a monolayer were cultured in DMEM, while cells growing in suspension were cultured in RPMI 1640. Cells were grown in humidified atmosphere under the conditions of 37 °C/5% CO_2_ gas in a CO_2_ incubator (IGO 150 CELLlife^TM^, JOUAN, Thermo Fisher Scientific, Waltham, MA, USA).

##### Proliferation Assay

Growth-inhibitory activity was assessed using a slightly modified procedure based on the National Cancer Institute’s protocol [54]. Briefly, cells were seeded in 96-well microtiter plates and incubated for 24 h. They were then treated with 10^−7^ to 10^−4^ M concentrations of the tested compounds for an additional 72 h. After the treatment period, the effects of the tested compounds on the growth rate of the cells were examined using the MTT assay [55]. Absorbance was measured at 595 nm using a microplate reader. The IC_50_ value, which represents a 50% inhibition of cell growth, and QC calculation were performed using the GraphPadPrism software (La Jolla, USA), v. 5.03. and Excel software, Office 365. The selectivity index was calculated according to the following formula:

$$SI=\frac{{IC}_{50}\;value\;of\;normal\;cell\;line}{{IC}_{50}\;value\;of\;cancer\;cell\;line}$$

The effect of each concentration was analyzed by plotting the logarithm of the concentration of the evaluated compound against the corresponding percentage inhibition value using least squares.

#### 3.2.2. Evaluation of Activity against Erythrocytic Stages of *P. falciparum*

The antiplasmodial activity of the new quinoline-benzomidazole hybrids was evaluated against two strains of *P. falciparum* (3D7—CQ-susceptible, provided by BEI resources, MRA-102 and Dd2—multiresistant, provided by BEI resources, MRA-159), as previously described, using the histidine-rich protein 2 (HRP2) assay [44,45]. In brief, 96-well plates were pre-coated with the tested compounds at 1:3 dilution before ring-stage parasites were added to the complete culture medium at a hematocrit of 1.5% and a parasitemia of 0.05%. After three days of incubation at 37 °C, 5% CO_2_ and 5% oxygen, the plates were frozen and thawed three times and analyzed by HRP2-ELISA. All compounds were analyzed in duplicate in at least two independent experiments. The IC50 was determined by nonlinear regression analysis of the logarithmic concentration-response curves using the drc package v3.0-1 of R v4.1.0 [56].

### 3.3. QSAR Analysis

QSAR analysis was performed on the antiplasmodial activities against 3D7 and Dd2 strains of *P. falciparum*. IC_50_ values (nM) were converted into their logarithmic form. The optimization of molecular structures was performed using HyperChem 8.0 (HyperCube, Inc., Gainesville, FL, USA) with two methods: molecular mechanics force fields (MM+) [57] and the semi-empirical PM3 method [58]. The molecular descriptors were generated using Parameter Client (Virtual Computational Chemistry Laboratory, https://vcclab.org/lab/pclient/+, (accessed on 10 February 2024). The initial number of 1415 calculated descriptors were reduced to 470, excluding the descriptors that were too intercorrelated (>85%), using QSARINS-Chem 2.2.1 (University of Insubria, Varese, Italy). Generation of the QSAR model was performed using QSARINS with a genetic algorithm limiting the number of descriptors in the model to three. The initial set of molecules was split on training and test by random and ranking methods (20% in the test set). The models were assessed by fitting criteria, internal cross-validation, and external validation. The reliability of the molecules for the prediction was tested by playing the molecules into the applicability domain. Williams plots were used to detect the outliers and molecules outside of warning leverage (h*) [59].

## 4. Conclusions

Target amidine- and non-amidine-substituted 7-chloro-4-aminoquinoline–benzimidazole hybrids **10a**–**15d** were designed and prepared by the oxidative coupling of *o*-phenylenediamines with 7-chloro-4-aminoquinoline benzaldehydes **3**–**8**. A biological evaluation showed that that all three structural features (length of the linker, substituents at phenoxy, and benzimidazole moiety) had significant effects on the antiplasmodial effect of the prepared compounds.

Robust, stable, and predictive QSAR models were generated for antiplasmodial activity against 3D7 and Dd2 strains of *P. falciparum.* Important structural features of 7-chloroquinoline–benzimidazole hybrids for antiplasmodial activity are the spatial distribution of the atomic van der Waals volume, the enhanced sum of the geometrical distances between the N and Br atoms, the lower frequency of the presence of C and N atoms at the topological distance 10, and the higher ratio of the number of rotatable bonds to the total number of bonds as well. QSAR models pointed out the unfavorable effects of the amidine group on antiplasmodial activity.

The influence of specific structural modifications on the antiproliferative and antiplasmodial activity of 7-chloro-4-aminoquinoline–benzimidazole hybrids suggests possible avenues for the development of selective anticancer and antiplasmodial agents.

## Figures and Tables

**Table 1:** Sensitivity of human tumor and normal cells to investigated compounds expressed as IC_50_  ^a^ value.

			IC_50_ ^a^ (µM)						
Comp.	n	R_1_	R_2_	MRC-5	HeLa	CaCo-2	THP-1	Hut78	HL-60
**10a**	**1**	**H**	**H**	3.1 ± 0.3	3.4 ± 0.5	6.1 ± 4.9	2.4 ± 0.2	2.8 ± 0.5	1.3 ± 0.9
**10b**	**1**	**H**	**Cl**	4.0 ± 1.1	2.7 ± 0.2	1.9 ± 0.2	3.4 ± 0.8	1.6 ± 1.2	2.0 ± 0.4
**10c**	**1**	**H**	**OCH_3_**	4.9 ± 1.7	2.6 ± 0.4	1.7 ± 0.1	3.8 ± 0.4	2.3 ± 0.8	1.8 ± 0.5
**10d**	**1**	**H**	**Am**	99.2 ± 8.3	>100 ^b^	>100 ^b^	>100	92± 6.1	>100
**11a**	**1**	**Br**	**H**	2.2 ± 0.8	2.6 ± 0.2	2.1 ± 0.6	4.9 ±7.3	2.4 ± 0.6	0.3 ± 0.3
**11b**	**1**	**Br**	**Cl**	3.0 ± 0.9	5.4 ± 4.6	13.3 ± 2.5	4.4 ±5.1	5.8 ± 1.9	1.3 ± 0.9
**11c**	**1**	**Br**	**OCH_3_**	5.3 ± 2.4	2.5 ± 0.4	1.5 ± 0.3	3.5 ±7.3	3.0 ± 1.1	2.2 ± 0.4
**11d**	**1**	**Br**	**Am**	>100	>100	>100	33.9 ± 3.6	17.3 ± 9.2	44.3 ±1.2
**12a**	**1**	**OCH_3_**	**H**	3.8 ± 0.4	2.2 ± 0.1	2.0 ± 0.3	3.0 ± 0.6	4.2 ± 2.1	2.0 ± 0.3
**12b**	**1**	**OCH_3_**	**Cl**	3.0 ± 1.6	5.6 ± 1.3	4.7 ± 4.0	6.1 ± 6.9	3.7 ± 5.2	3.3 ± 1.1
**12c**	**1**	**OCH_3_**	**OCH_3_**	1.1 ± 0.8	2.7 ± 0.2	2.1 ± 0.3	3.1 ± 0.8	2.4 ± 1.1	0.2 ± 0.0
**12d**	**1**	**OCH_3_**	**Am**	>100	>100	>100	100	16.1 ± 4.7	52.5 ± 5.1
**13a**	**2**	**H**	**H**	0.3 ± 0.2	3.0 ± 2.3	1.1 ± 0.4	3.0 ± 0.8	1.6 ± 0.8	0.4 ± 0.2
**13b**	**2**	**H**	**Cl**	0.4 ± 0.1	0.6 ± 0.1	1.6 ± 0.4	2.1 ± 0.7	0.3 ± 0.2	0.2 ± 0.1
**13c**	**2**	**H**	**OCH_3_**	2.7 ± 0.5	0.2 ± 0.2	1.0 ± 0.5	2.6 ± 0.0	1.1 ± 0.5	0.3 ± 0.2
**13d**	**2**	**H**	**Am**	83.0 ± 5.4	>100	>100	14.4 ± 3.1	15.2 ± 3.0	18.2 ± 6.7
**14a**	**2**	**Br**	**H**	0.6 ± 0.6	1.2 ± 0.7	5.0 ± 7.3	5.1 ± 1.7	1.7 ± 0.4	0.7 ± 0.5
**14b**	**2**	**Br**	**Cl**	2.0 ± 1.8	1.5 ± 1.0	1.5 ± 1.1	6.8 ± 3.5	0.9 ± 0.6	0.4 ± 0.1
**14c**	**2**	**Br**	**OCH_3_**	0.4 ± 0.1	0.8 ± 0.7	1.6 ± 0.9	5.2 ± 1.8	0.6 ± 0.1	0.3 ± 0.1
**14d**	**2**	**Br**	**Am**	>100	>100	>100	100	19.7 ± 4.0	24.4 ± 4.4
**15a**	**2**	**OCH_3_**	**H**	0.3 ± 0.1	0.4 ± 0.1	0.8 ± 0.1	2.2 ± 0.5	1.5 ± 0.3	0.4 ± 0.1
**15b**	**2**	**OCH_3_**	**Cl**	0.3 ± 0.0	0.3 ± 0.1	1.1 ± 0.6	2.5 ± 0.3	0.7 ± 0.4	0.2 ± 0.1
**15c**	**2**	**OCH_3_**	**OCH_3_**	0.7 ± 0.6	1.2 ± 0.6	1.5 ± 0.3	2.2 ± 0.3	0.7 ± 0.6	0.2 ± 0.0
**15d**	**2**	**OCH_3_**	**Am**	95.2 ± 9.7	>100	>100	29.6 ± 0.3	12.8 ± 5.5	19.5 ± 1.1
**5-FU**				54.1 ^c^	8.2 ^c^	5.9 ^c^	>100 ^c^	>100 ^c^	76.4 ^c^

^a^ IC_50_—Concentration of the compound that inhibits cell growth by 50%. Data represent mean IC_50_ (μM) values ± standard deviation (SD) from three independent experiments. Exponentially growing cells were treated with compounds during 72 h. Cytotoxicity was analyzed using MTT survival assay. ^b^ For previously published results, see reference [18]. ^c^ For previously published results, see reference [19]. **5-FU**: 5-fluorouracil.

**Table 2:** In vitro antiplasmodial activity of quinoline–benzimidazole hybrids against the erythrocytic stage of *P. falciparum* (*Pf*3D7 and *Pf*Dd2 strains).

				IC_50_ ^a^ (nM)	
Comp.	n	R_1_	R_2_	*Pf*3D7	*Pf*Dd2
**10a**	**1**	**H**	**H**	2.7 ± 0.2	3.3 ± 0.1
**10b**	**1**	**H**	**Cl**	3.5 ± 1.5	4.9 ± 0.8
**10c**	**1**	**H**	**OCH_3_**	6.8 ± 1.8	10.1 ± 5.0
**10d**	**1**	**H**	**Am**	58.5 ± 5.9	36.9 ± 17.7
**11a**	**1**	**Br**	**H**	36.9 ± 1.5	8.7 ± 3.4
**11b**	**1**	**Br**	**Cl**	87.3 ± 3.3	88.3 ± 3.1
**11c**	**1**	**Br**	**OCH_3_**	19.7 ± 4.2	28.0 ± 0.6
**11d**	**1**	**Br**	**Am**	321.4 ± 32.8	984.5 ± 520.8
**12a**	**1**	**OCH_3_**	**H**	14.5 ± 4.0	91.2 ± 9.5
**12b**	**1**	**OCH_3_**	**Cl**	11.1 ± 0.3	21.5 ± 2.5
**12c**	**1**	**OCH_3_**	**OCH_3_**	9.7 ± 0.2	15.4 ± 2.0
**12d**	**1**	**OCH_3_**	**Am**	190.5 ± 11.5	120.0 ± 7.5
**13a**	**2**	**H**	**H**	2.6 ± 0.7	4.8 ± 0.3
**13b**	**2**	**H**	**Cl**	2.4 ± 1.4	3.9 ± 1.1
**13c**	**2**	**H**	**OCH_3_**	12.8 ± 0.5	11.9 ± 1.6
**13d**	**2**	**H**	**Am**	57.4 ± 1.9	198.3 ± 0.1
**14a**	**2**	**Br**	**H**	9.3 ± 0.9	13.0 ± 1.9
**14b**	**2**	**Br**	**Cl**	5.0 ± 1.4	14.2 ± 1.2
**14c**	**2**	**Br**	**OCH_3_**	11.4 ± 4.0	11.8 ± 3.2
**14d**	**2**	**Br**	**Am**	158.6 ± 22.6	450.8 ± 40.3
**15a**	**2**	**OCH_3_**	**H**	4.4 ± 0.3	11.2 ± 0.5
**15b**	**2**	**OCH_3_**	**Cl**	4.7 ± 1.5	5.2 ± 0.5
**15c**	**2**	**OCH_3_**	**OCH_3_**	6.9 ± 1.7	16.5 ± 5.7
**15d**	**2**	**OCH_3_**	**Am**	28.7 ± 9.4.	22.9 ± 4.3
**CQ**				11.1 ± 0.5	360.0 ± 27.15

^a^ IC_50_—compound concentration inhibited cell growth by 50%. Data represent mean IC_50_ (nM) values ± standard deviation (SD), n ≥ 2. **CQ**: Chloroquine.

**Table 3:** The statistical results of QSAR models for antiplasmodial activities against the 3D7 and Dd2 strains of *Plasmodium falciparum*.

Statistical Parameters	Model 1	Model 2	Validation Criteria Thresholds
*N_tr_*	20	20	
*N_ext_*	5	5	
*R* ^2^	0.886	0.859	≥0.6
*R* ^2^_adj_	0.865	0.832	≥0.6
*s*	0.219	0.288	as low as possible
*F*	41.589	32.525	significant at *p* < 0.05
*Kxx*	0.053	0.217	as low as possible
Δ*K*	0.328	0.102	≥0.05
*RMSE_tr_*	0.196	0.257	close to zero
*MAE_tr_*	0.159	0.207	close to zero
*CCC_tr_*	0.940	0.924	≥0.80
*Q* ^2^ *_LOO_*	0.840	0.799	>0.5
*RMSE_cv_*	0.232	0.307	*RMSE_tr_* < *RMSE_cv_*
*MAE_cv_*	0.192	0.256	close to zero
*CCC_cv_*	0.915	0.889	≥ 0.80
*R* ^2^ *_Yscr_*	0.160	0.158	< 0.2
*Q* ^2^ *_Yscr_*	−0.341	−0.353	*R*^2^*_yscr_* > *Q*^2^*_yscr_*
*RMSE_ext_*	0.201	0.266	close to zero
*MAE_ext_*	0.147	0.250	close to zero
*R* ^2^ *_ext_*	0.937	0.878	≥ 0.6
*CCC_ext_*	0.934	0.890	≥ 0.80
*Q* ^2^ *_F_*_1_	0.908	0.808	> 0.6
*Q* ^2^ *_F_*_2_	0.893	0.797	> 0.6
*Q* ^2^ *_F_*_3_	0.880	0.850	> 0.6
*r*^2^*_m_* average	0.685	0.827	≥ 0.5
*r*^2^*_m_* difference	0.111	0.030	as low as possible
Applicability domain			
*N* compounds outlier	-	1 (**12a**)	
*N* compounds out of app.dom.	1 (**14d**)	-	
h*	0.600	0.600	

*N_tr_* (number of compounds in the training set); *N_ext_* (number of compounds in the test set); LOO *(*leave-one-out); *R*^2^ (coefficient of determination); *R*^2^*_adj_* (adjusted coefficient of determination); *s* (standard deviation of regression); *F* (Fisher ratio); *Kxx* (global correlation among descriptors); Δ*K* (global correlation among descriptors); *RMSE_tr_* (root-mean-square error of the training set); *MAE_tr_* (mean absolute error of the training set); *CCC_tr_* (concordance correlation coefficient of the training set); *Q*^2^*_LOO_* (cross-validated explained variance); *RMSE_cv_* (root-mean-square error of the training set determined through the cross validated method; *MAE_cv_* (mean absolute error of the internal validation set); *CCC_cv_* (concordance correlation coefficient test set using cross validation); *R*^2^*_Yscr_* (Y-scramble correlation coefficients); *Q*^2^*_Yscr_* (Y-scramble cross-validation coefficients); *RMSE_ext_* (root-mean-square error of the external validation set); *MAE_ext_* (mean absolute error of the external validation set); *R*^2^*_ext_* (coefficient of determination of validation set); *Q*^2^*_F_*_1_, *Q*^2^*_F_*_2_, *Q*^2^*_F_*_3_ (predictive squared correlation coefficients); *CCC_ext_* (concordance correlation coefficient of the test set); *r*^2^*_m_* average (average value of squared correlation coefficients between the observed and (leave-one-out) predicted values of the compounds with and without intercept); *r*^2^*_m_* difference (absolute difference between the observed and leave-one-out predicted values of the compounds with and without intercept); h* (warning leverage of applicability domain).

## Data Availability

The original contributions presented in the study are included in the article/Supplementary Material, further inquiries can be directed to the corresponding author/s.

## Supplementary Materials

The following supporting information can be downloaded at https://www.mdpi.com/article/10.3390/molecules29132997/s1, Table S1: Experimental and calculated log3D7 by model (1), with values of decriptors included in the model.; Table S2: Experimental and calculated logDd2 by model (2). with values of decriptors included in the model.
