# Supplementary material for: Design, Synthesis, Antitumor, and Antiplasmodial Evaluation of New 7-Chloroquinoline–Benzimidazole Hybrids
Source: Molecules. 2024 Jun 24;29(13):2997. doi: 10.3390/molecules29132997 (PMC11243327; doi:10.3390/molecules29132997)
Supplement: Supplementary file 1 [file molecules-29-02997-s001.zip › Supplementary Molecules_new.pdf]

Supplementary

Design, synthesis, antitumor, and antiplasmodial evaluation of new 7-chloroquinoline-benzimidazole hybrids

Luka Krstulović[1*], Vesna Rastija[2], Lais Pessanha de Carvalho[3], Jana Held[3,7], Miroslav Bajić[1], Zrinka Rajić[4], Zorislava Živković[5], Ljubica Glavaš-Obrovac[6, *]

[1] Department of Chemistry and Biochemistry, Faculty of Veterinary Medicine, University of Zagreb, Heinzelova 55, HR-10000 Zagreb, Croatia

[2] Department of Chemistry, Faculty of Agrobiotechnical Sciences Osijek, Josip Juraj Strossmayer University of Osijek, Vladimira Preloga 1, 31000 Osijek, Croatia

[3] Institute of Tropical Medicine, University of Tübingen, Tübingen, Germany

[4] Department of Medicinal Chemistry, Faculty of Pharmacy and Biochemistry, University of Zagreb, Croatia

[5] General County Hospital of Našice, Bana Jelačića 10, 31500 Našice

[6] Department of Medicinal Chemistry, Biochemistry and Clinical Chemistry, Faculty of Medicine Osijek, Josip Juraj Strossmayer University of Osijek, J. Huttlera 4, HR-31000 Osijek, Croatia

[7] German Center for Infection Research (DZIF), Partner Site Tübingen, Tübingen, Germany;

* Correspondence: lkrstulovic@vef.hr (L. Krstulović), lgobrovac@mefos.hr (Lj. Glavaš-Obrovac),

Table S1. Experimental and calculated log3D7 by model (1), with values of decriptors included in the model.

ID	Name	Status	Exp. endpoint	Pred. by model eq.(1)	*GATS7v*	*G(N..Br)*	*RDF135 v*
1	**10a**	Training	0.427	0.7372	1.375	0	1.477
2	**10b**	Test	0.544	0.9202	1.313	0	2.189
3	**10c**	Test	0.831	0.8254	1.334	0	1.695
4	**10d**	Training	1.767	1.4727	1.222	0	5.418
5	**11a**	Training	1.567	1.5998	1.26	21.951	4.613
6	**11b**	Training	1.941	1.7545	1.215	21.845	5.309
7	**11c**	Training	1.294	1.551	1.218	22.328	3.728
8	**11d**	Training	2.507	2.5346	1.132	43.852	8.164
9	**12a**	Training	1.162	1.0478	1.439	0	4.587
10	**12b**	Training	1.043	1.3329	1.389	0	6.219
11	**12c**	Training	0.988	0.812	1.374	0	2.042
12	**12d**	Training	2.28	2.1358	1.284	0	11.217
13	**13a**	Training	0.407	0.4299	1.851	0	4.462
14	**13b**	Training	0.387	0.6118	1.766	0	4.907
15	**13c**	Training	1.107	0.705	1.675	0	4.601
16	**13d**	Training	1.759	1.7516	1.53	0	11.025
17	**14a**	Training	0.968	0.9973	1.844	22.921	6.443
18	**14b**	Test	0.699	0.8416	1.777	25.4	4.242
19	**14c**	Training	1.057	0.8828	1.664	24.51	3.379
20	**14d**	Test	2.2	1.9995	1.542	45.921	8.446
21	**15a**	Test	0.646	0.6549	1.542	0	2.721
22	**15b**	Training	0.672	0.7674	1.488	0	2.98
23	**15c**	Training	0.831	0.867	1.457	0	3.398
24	**15d**	Training	1.457	1.7179	1.358	0	8.833
25	**CQ**	Training	1.043	0.9548	1.192	0	1.095

Table S2. Experimental and calculated logDd2 by model (2). with values of decriptors included in the model

ID	Name	Status	Exp endpoint	Pred by model eq	RBF	LAI	F10[C-N]
1	**10a**	Training	0.524	0.7946	0.113	0	6
2	**10b**	Training	0.69	0.7946	0.113	0	6
3	**10c**	Training	10.020	12.668	0.123	0	6
4	**10d**	Training	15.660	18.082	0.125	0	8
5	**11a**	Training	0.94	0.7946	0.113	0	6
6	**11b**	Training	19.460	17.290	0.113	1	6
7	**11c**	Test	14.470	12.668	0.123	0	6
8	**11d**	Training	29.930	27.426	0.125	1	8
9	**12a**	Training	19.600	12.668	0.123	0	6
10	**12b**	Training	13.320	12.668	0.123	0	6
11	**12c**	Training	11.880	16.445	0.131	0	6
12	**12d**	Training	20.790	21.387	0.132	0	8
13	**13a**	Training	0.677	0.4673	0.125	0	2
14	**13b**	Training	0.585	0.4673	0.125	0	2
15	**13c**	Training	10.760	0.845	0.133	0	2
16	**13d**	Training	22.970	22.263	0.133	1	4
17	**14a**	Training	11.140	14.016	0.125	1	2
18	**14b**	Training	11.510	14.016	0.125	1	2
19	**14c**	Test	10.700	0.845	0.133	0	2
20	**14d**	Test	26.540	22.263	0.133	1	4
21	**15a**	Test	10.470	0.845	0.133	0	2
22	**15b**	Training	0.716	0.845	0.133	0	2
23	**15c**	Training	12.170	12.227	0.141	0	2
24	**15d**	Test	13.600	15.753	0.139	0	4
25	**CQ**	Training	25.560	24.850	0.163	0	3

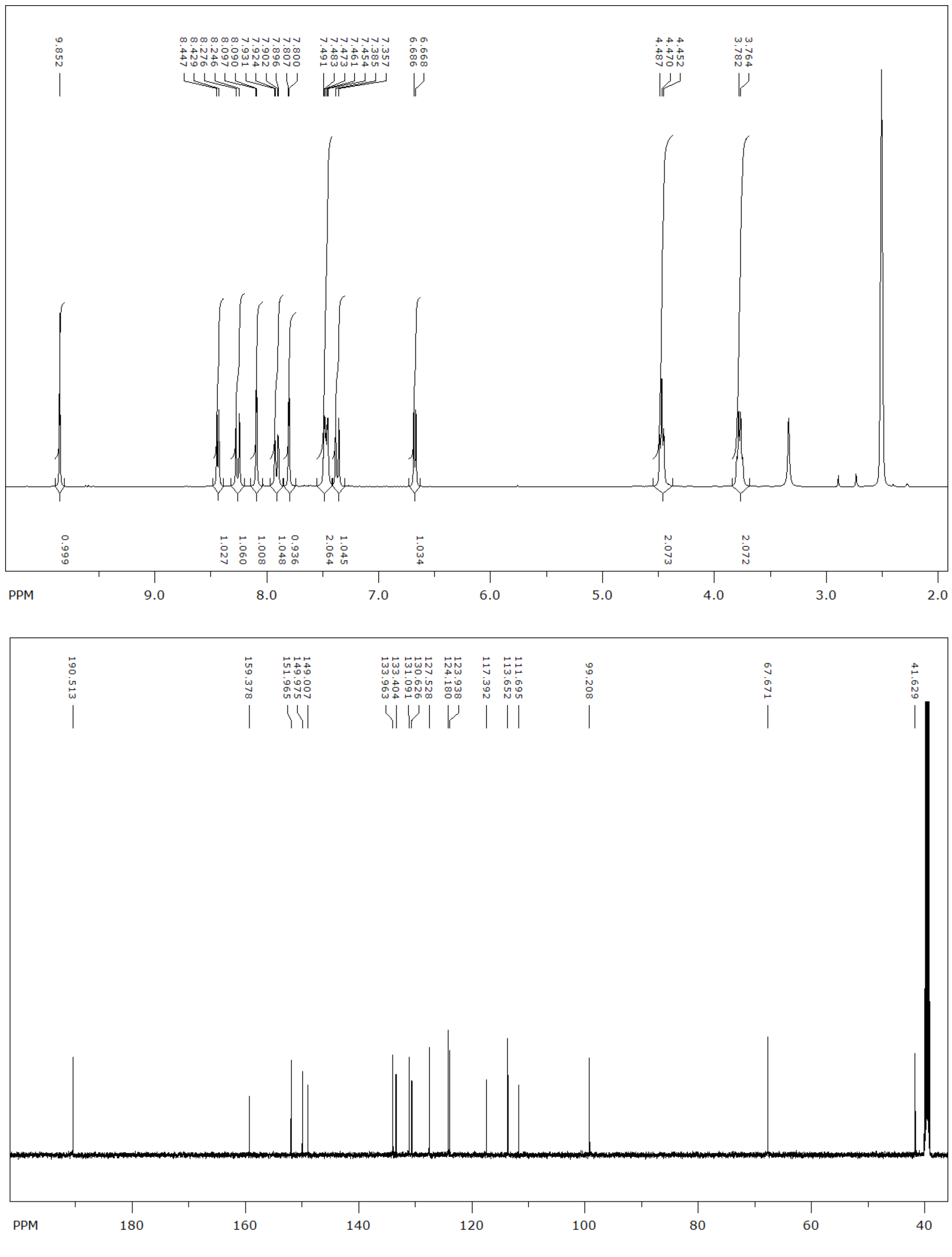

Figure S1 ^{1}H NMR and ^{13}C NMR of compound **4**

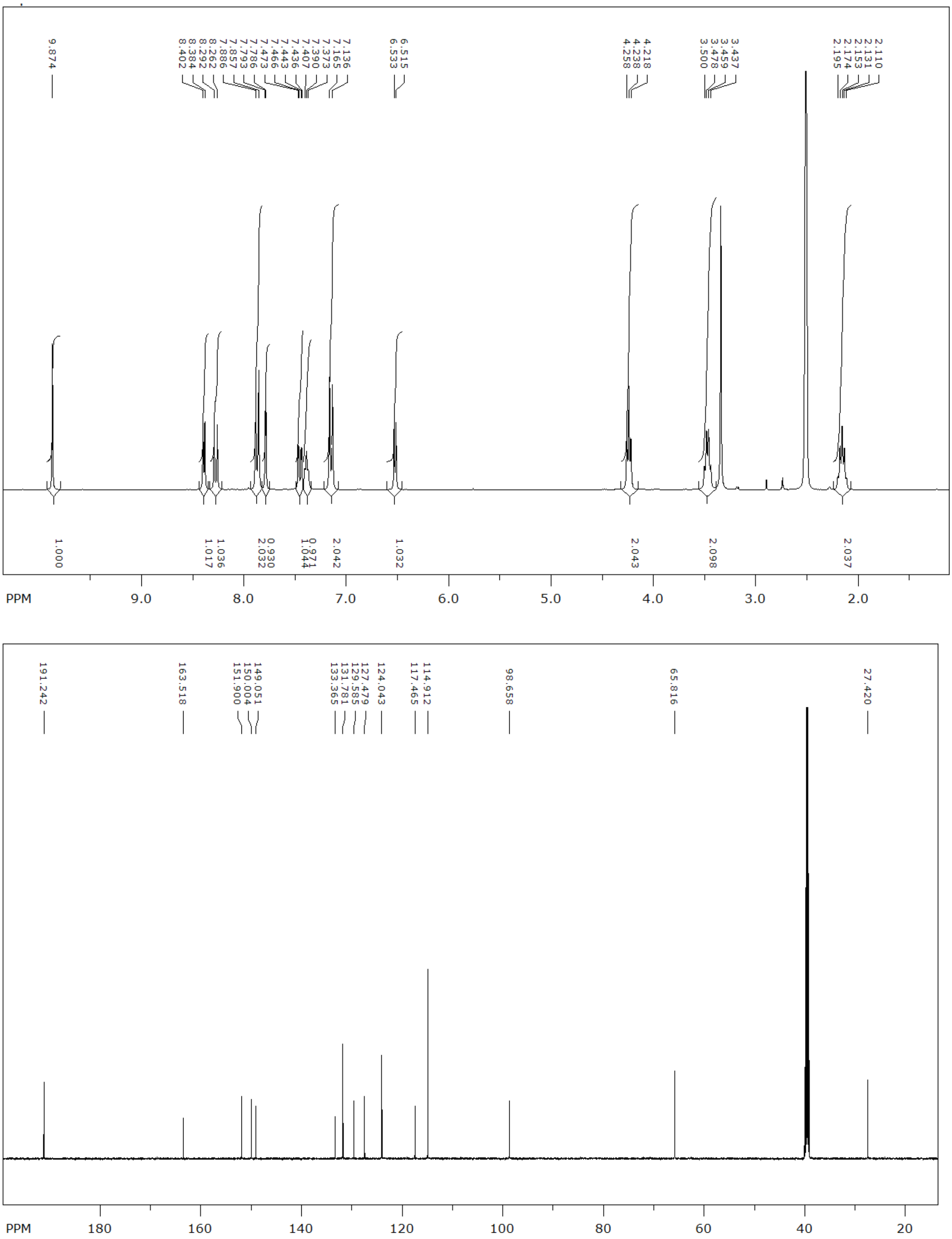

Figure S2 ^{1}H NMR and ^{13}C NMR of compound **6**

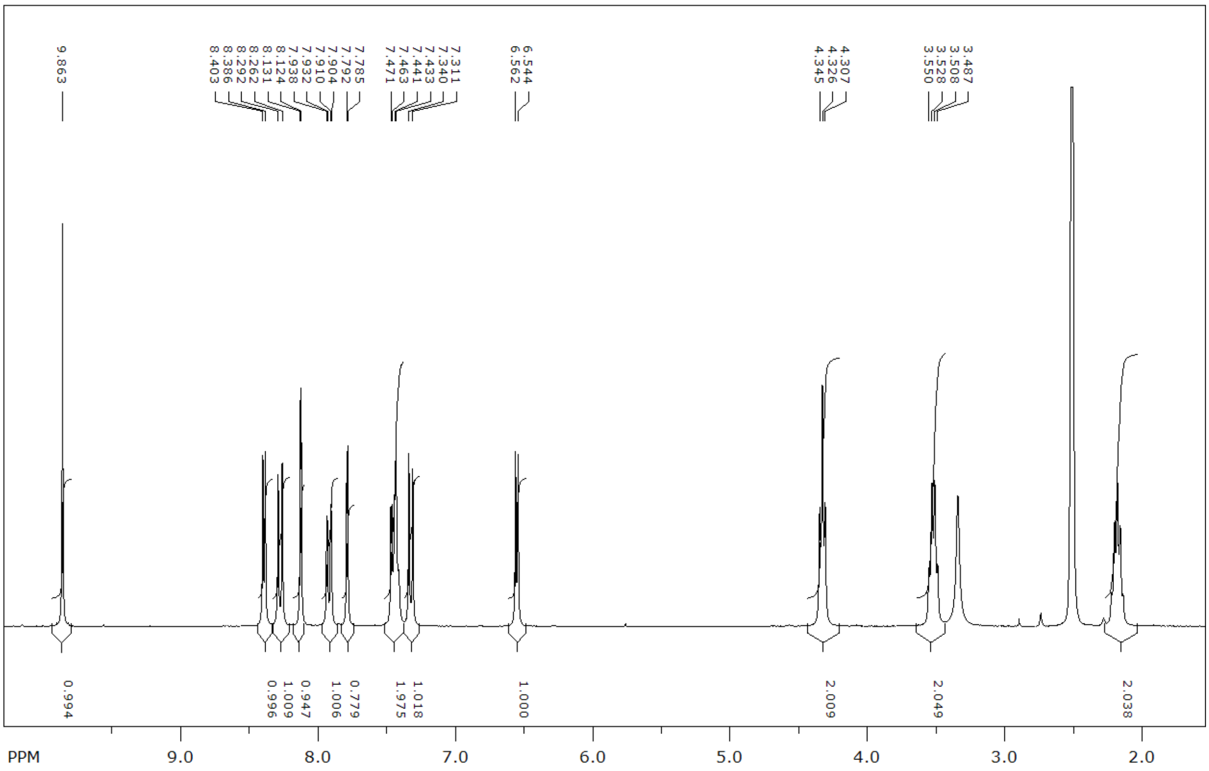

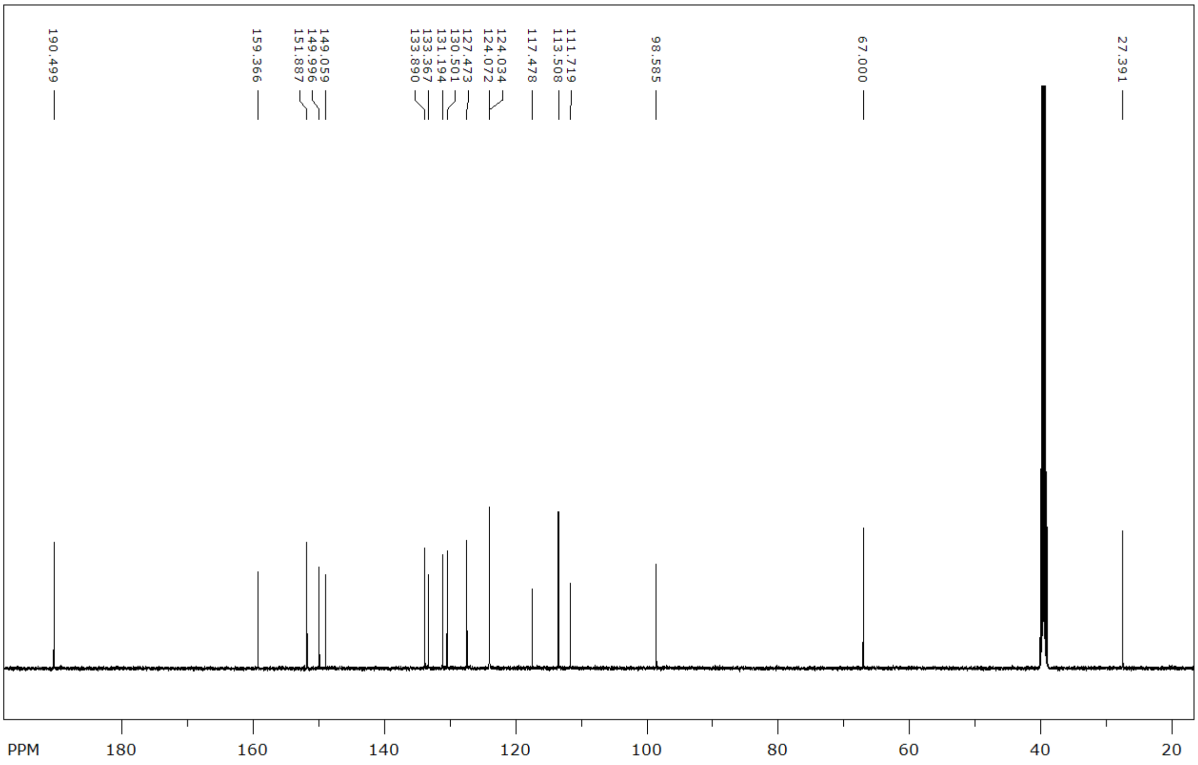

Figure S3 ^{1}H NMR and ^{13}C NMR of compound **7**

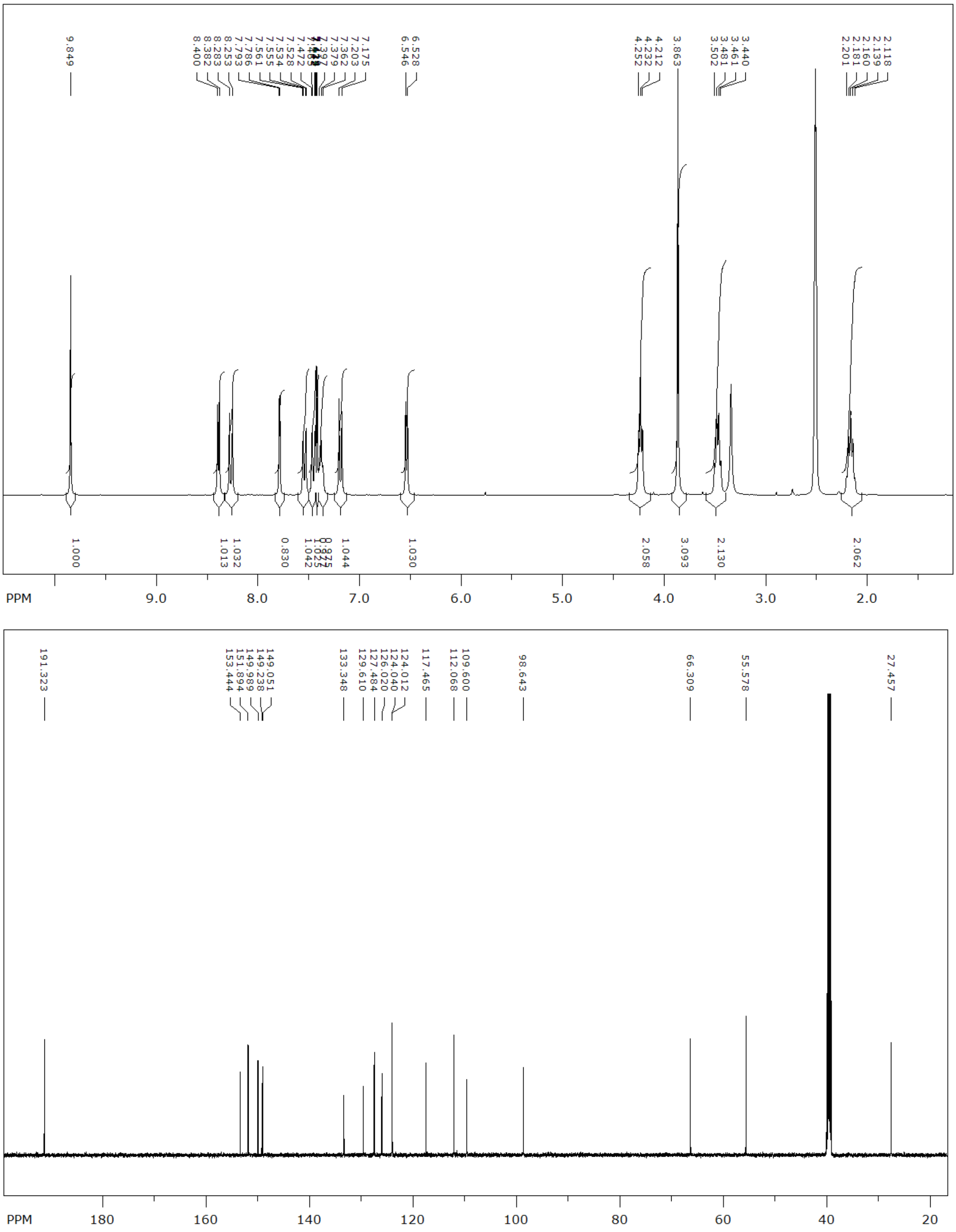

Figure S4 ^{1}H NMR and ^{13}C NMR of compound **8**

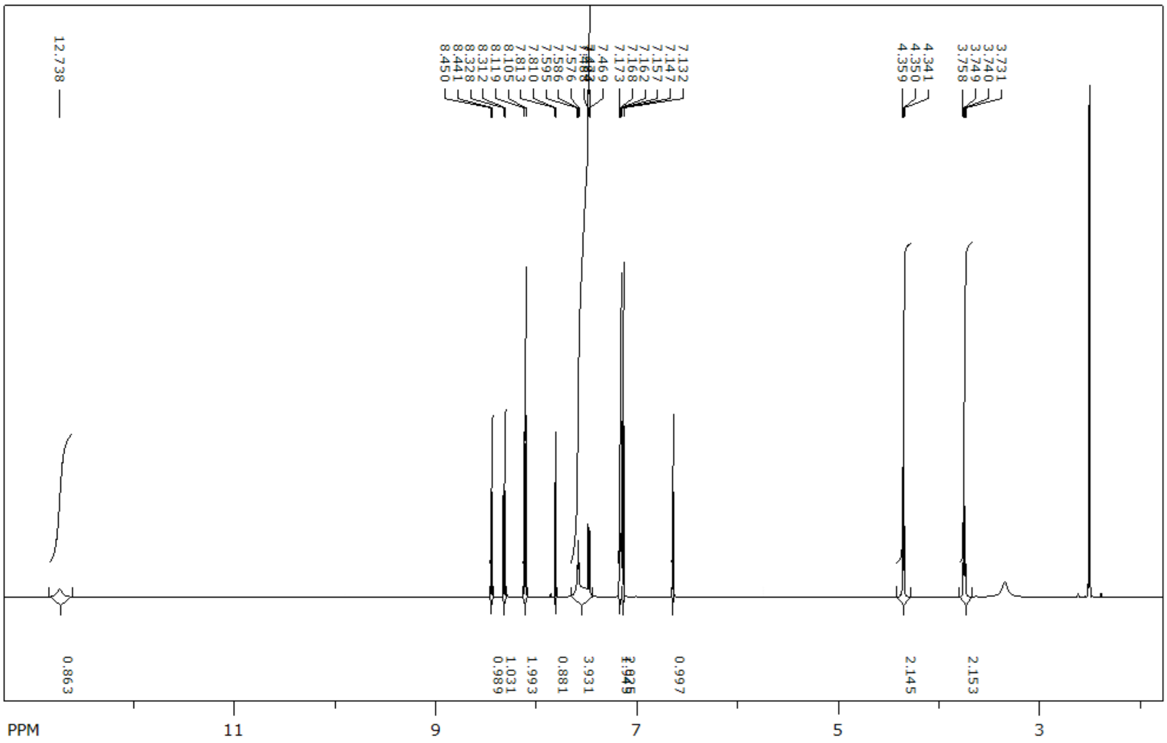

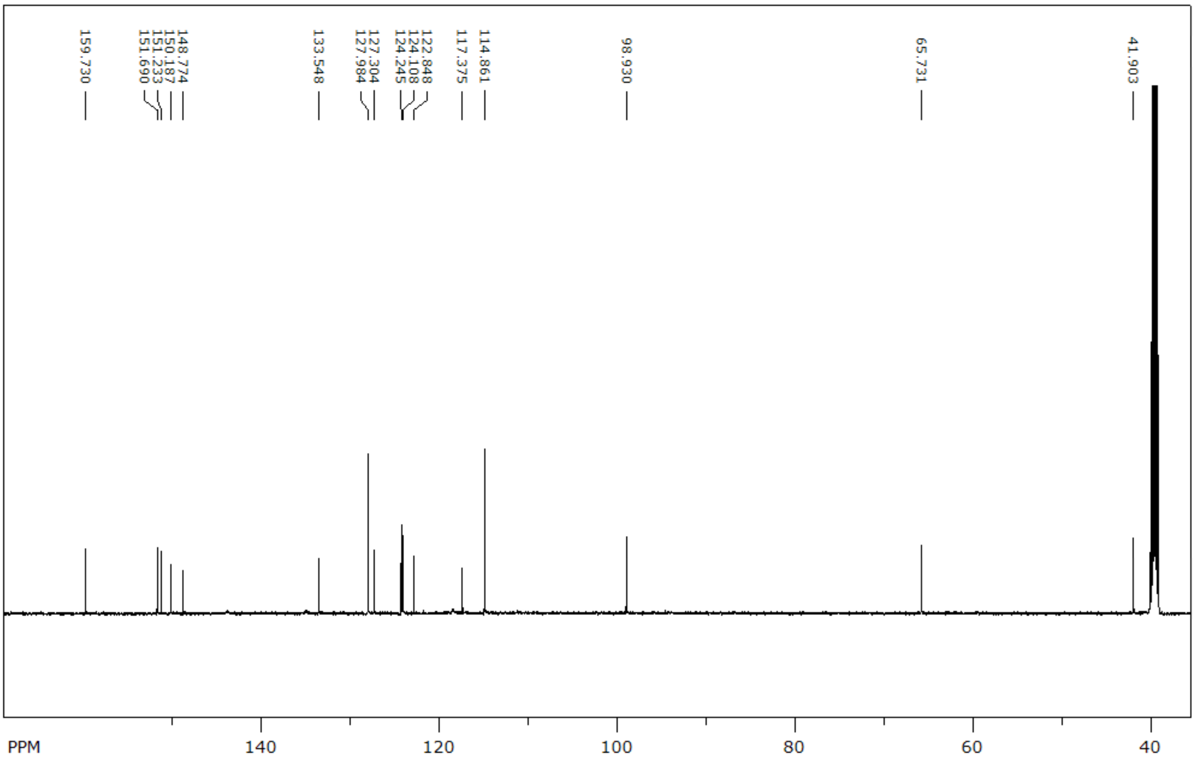

Figure S5 ^{1}H NMR and ^{13}C NMR of compound **10a**

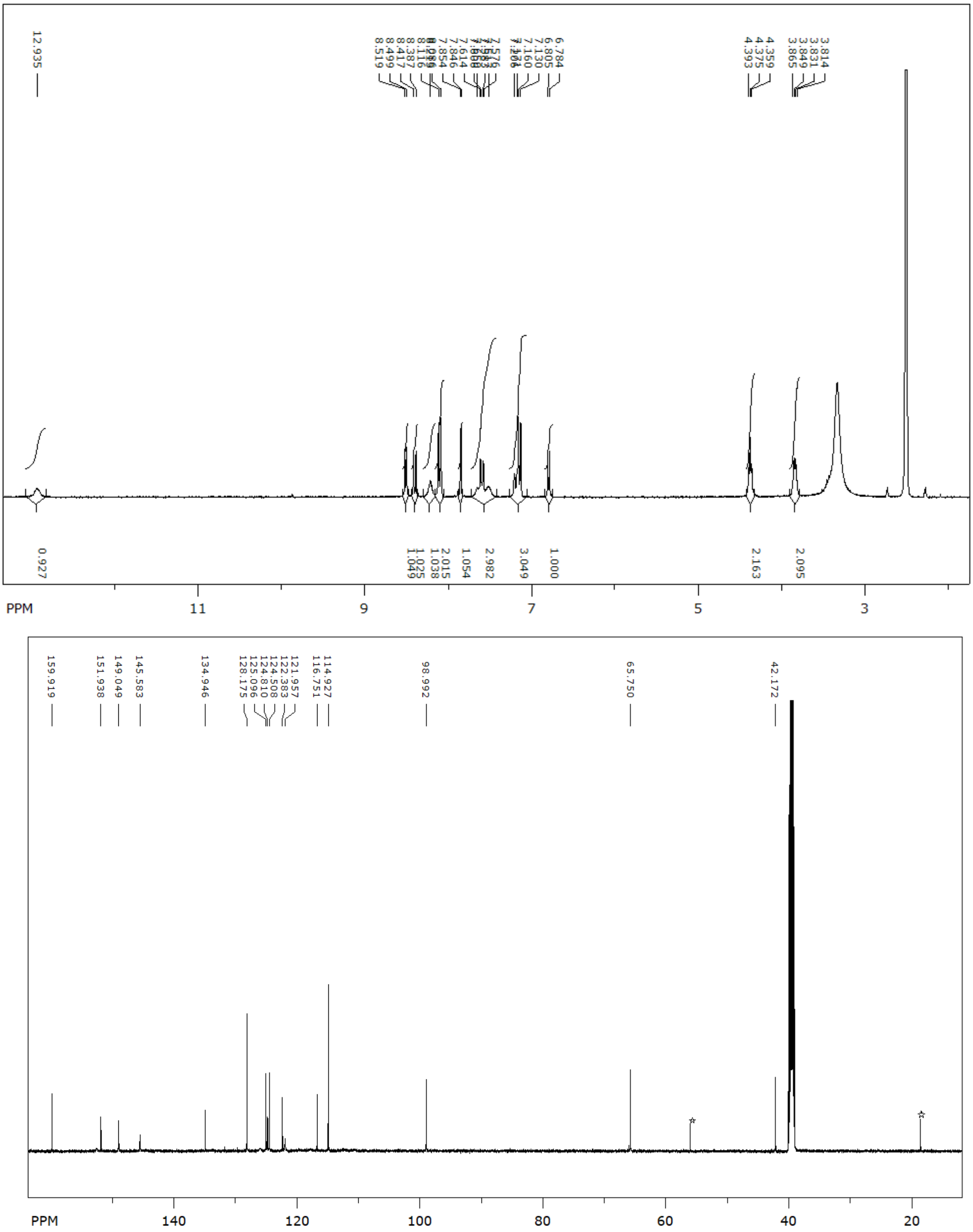

Figure S6 ^{1}H NMR and ^{13}C NMR of compound **10b** (*ethanol; residual solvent signals)

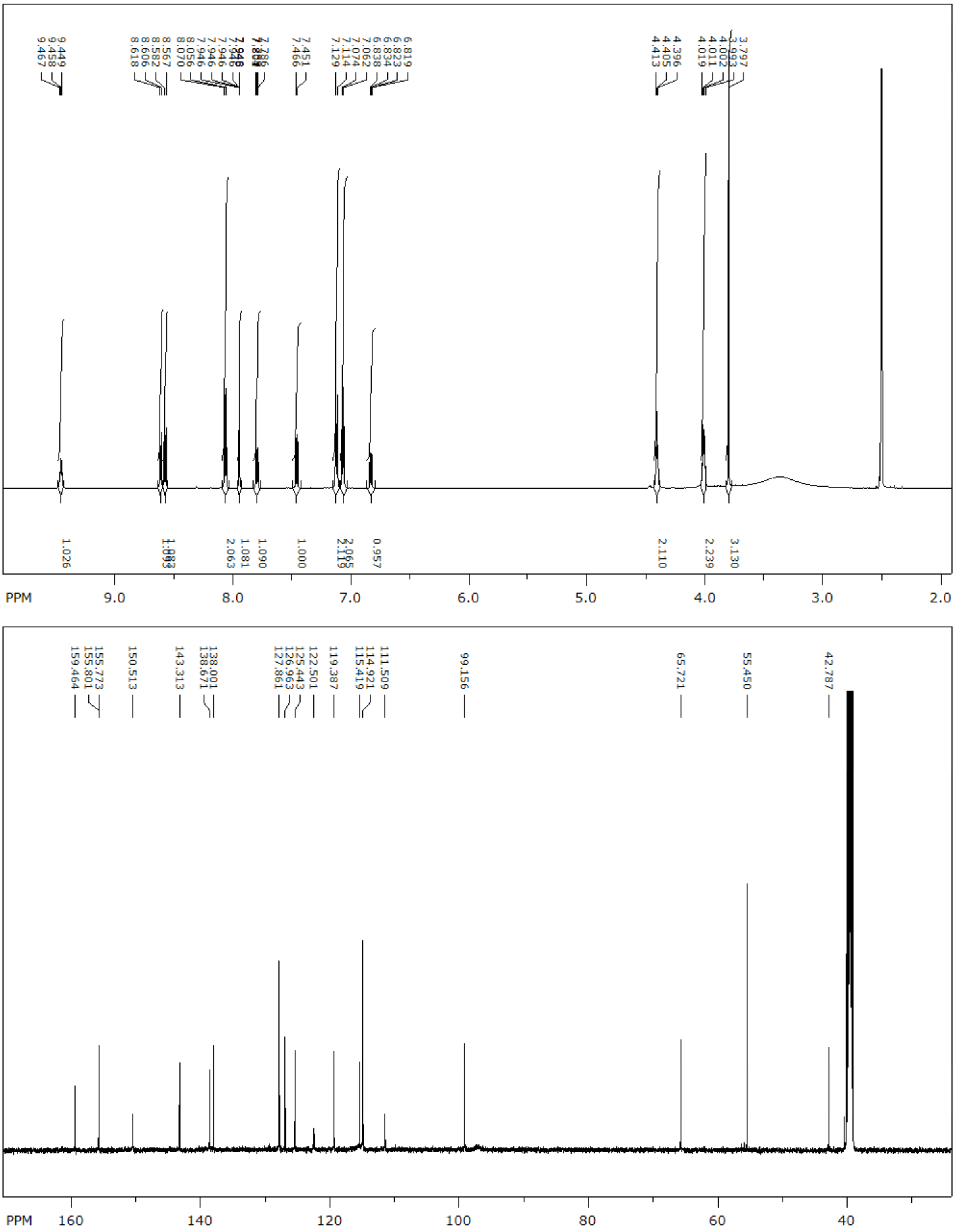

Figure S7 ^{1}H NMR and ^{13}C NMR of compound **10c**

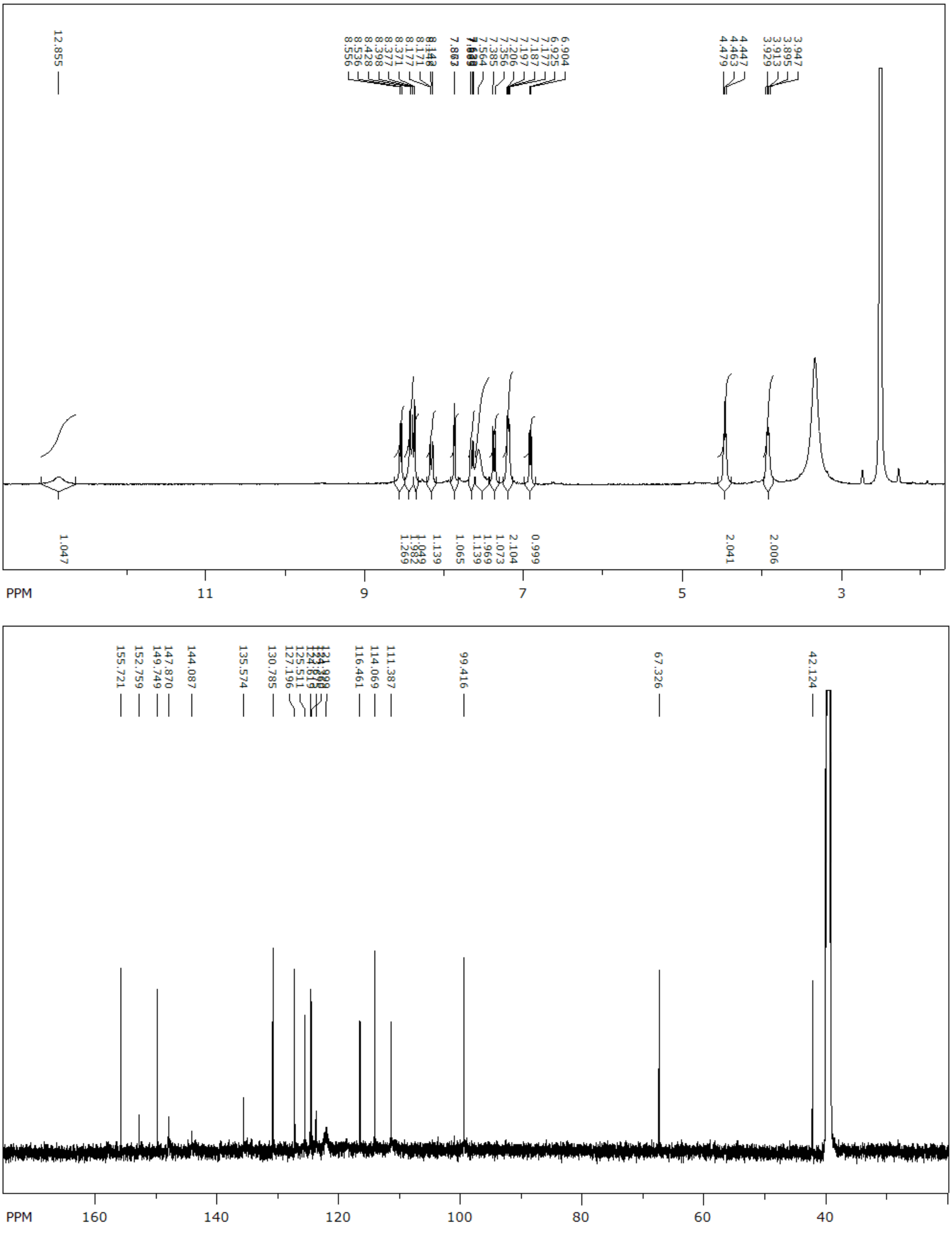

Figure S8 ^{1}H NMR and ^{13}C NMR of compound **11a**

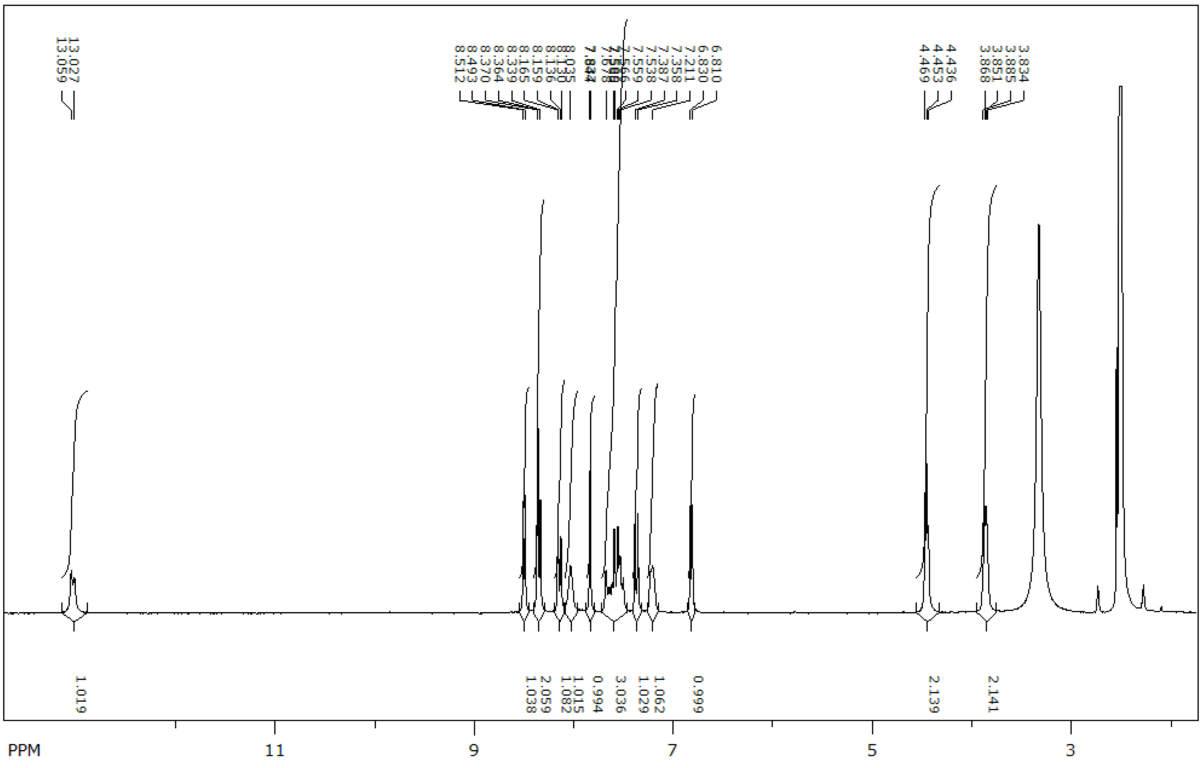

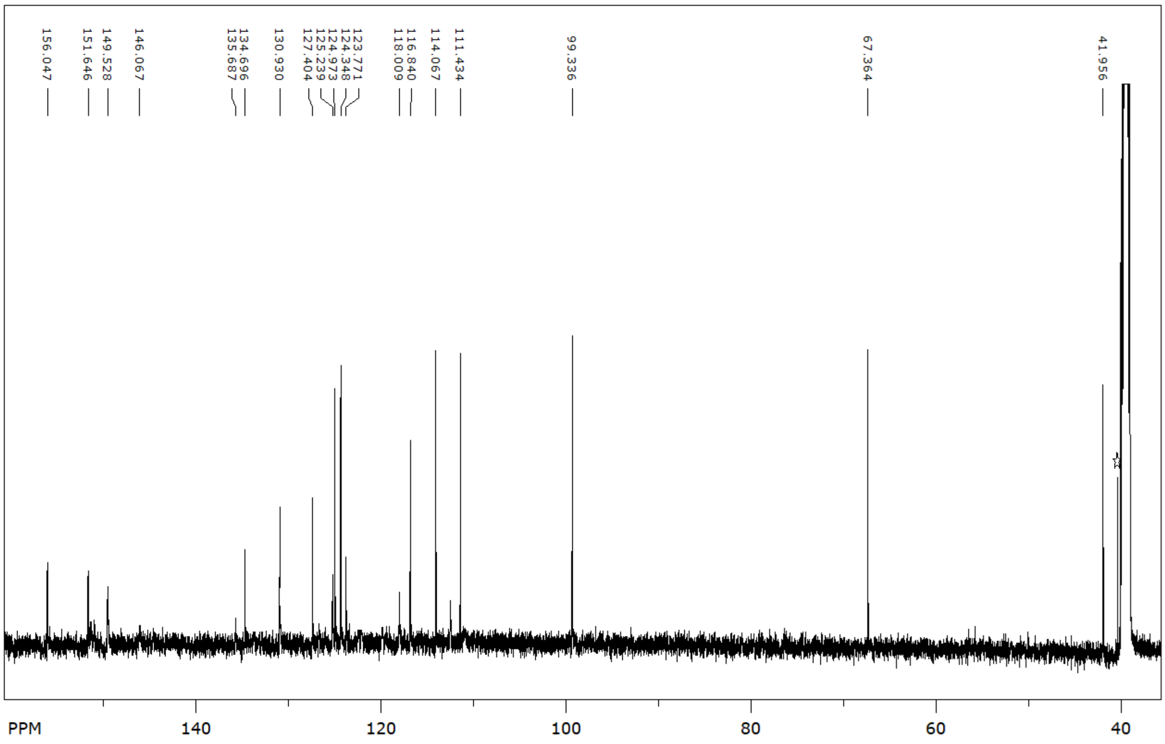

Figure S9 ^{1}H NMR and ^{13}C NMR of compound **11b** (*DMSO; residual solvent signal)

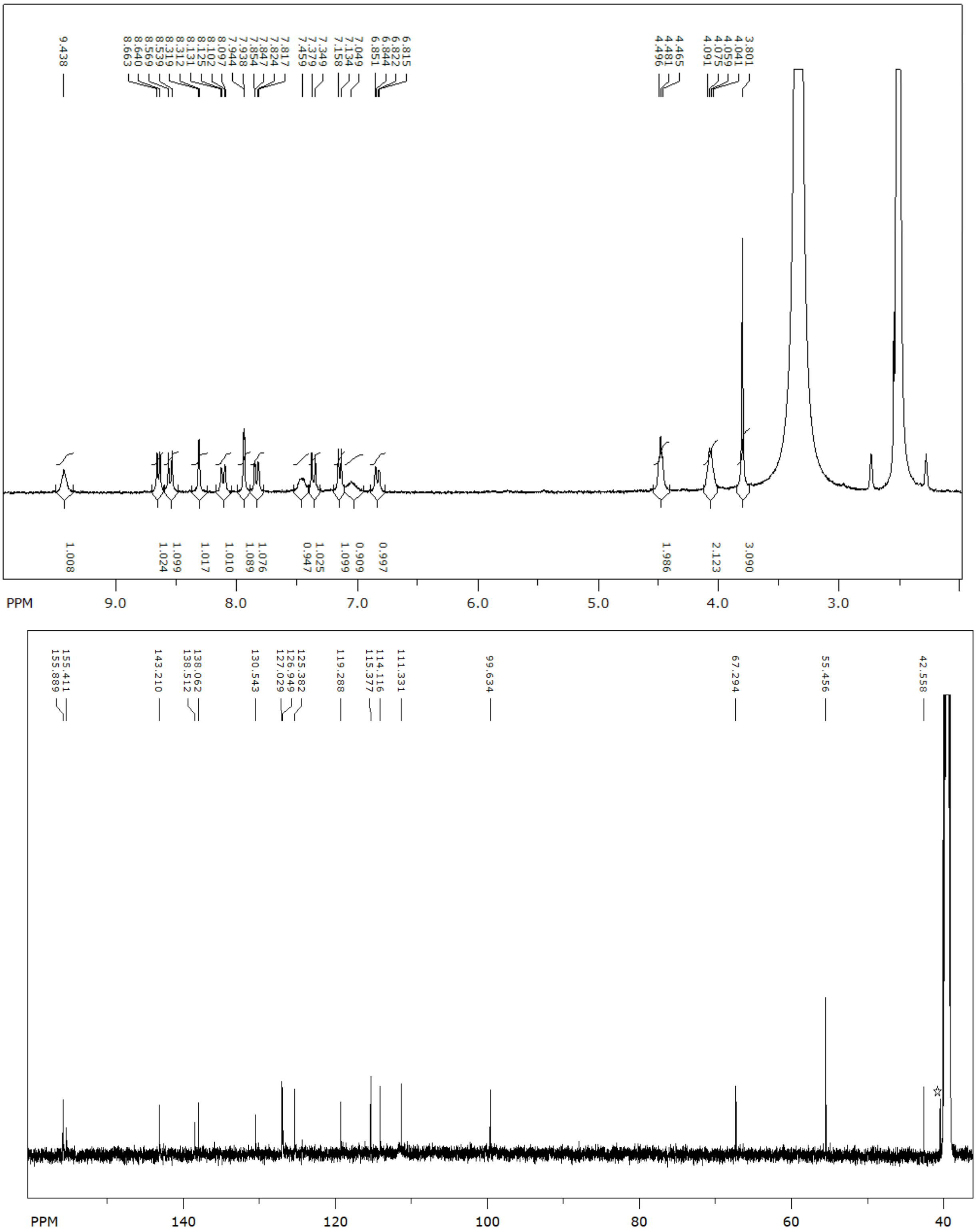

Figure S10 ¹H NMR and ¹³C NMR of compound **11c** (*DMSO; residual solvent signal)

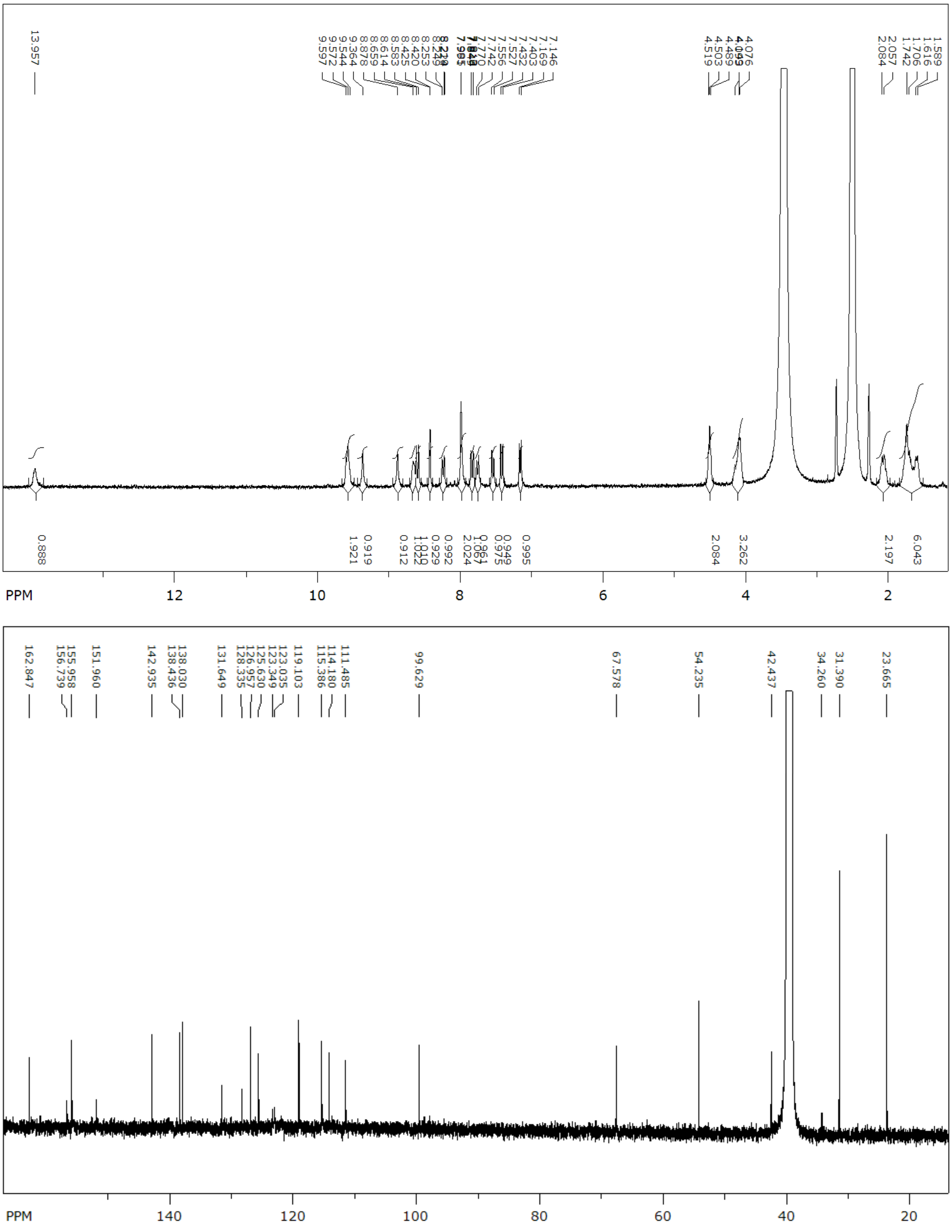

Figure S11 ^{1}H NMR and ^{13}C NMR of compound **11d**

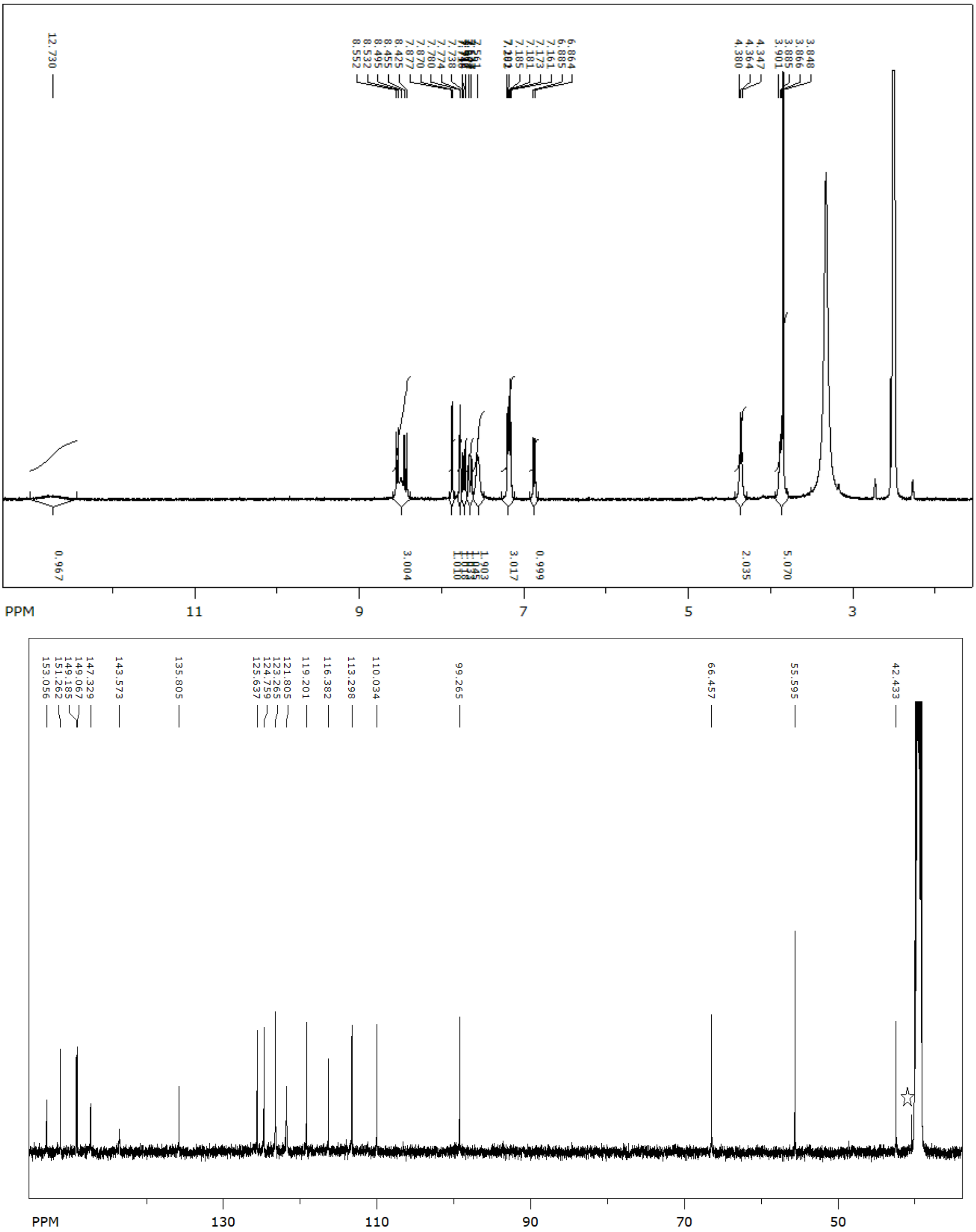

Figure S12 ^{1}H NMR and ^{13}C NMR of compound **12a** (*DMSO; residual solvent signal)

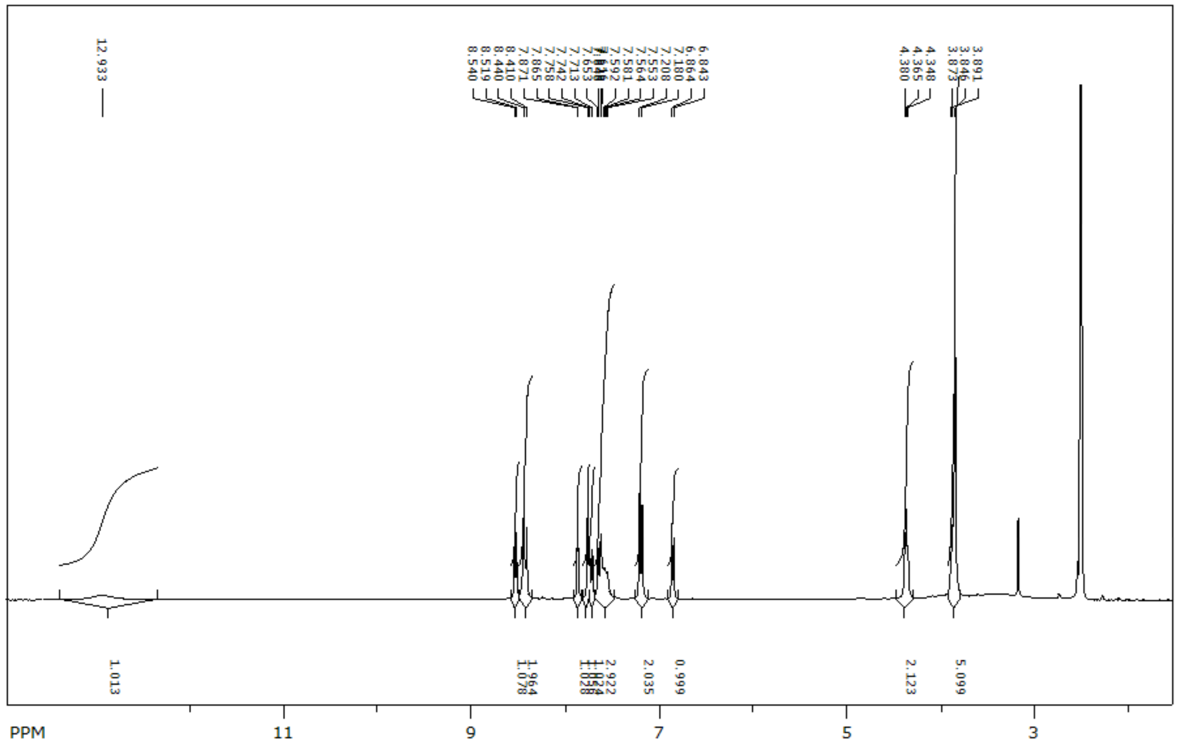

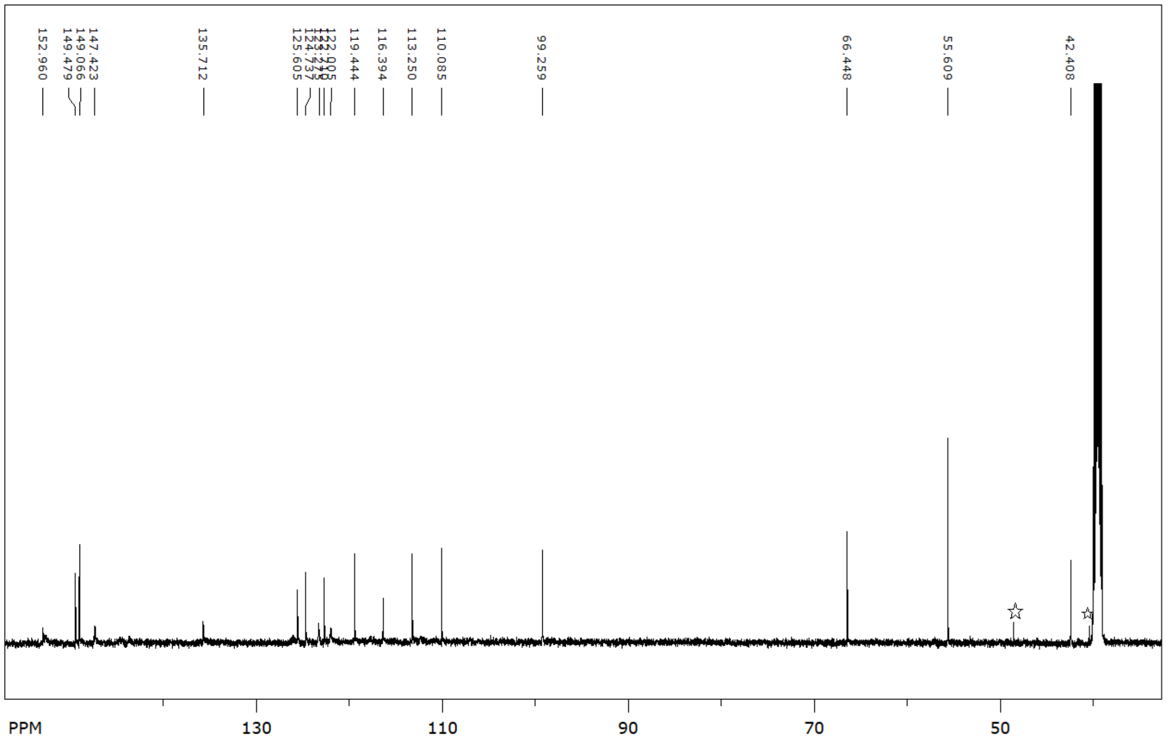

Figure S13 ^{1}H NMR and ^{13}C NMR of compound **12b** (*methanol and DMSO; residual solvent signals)

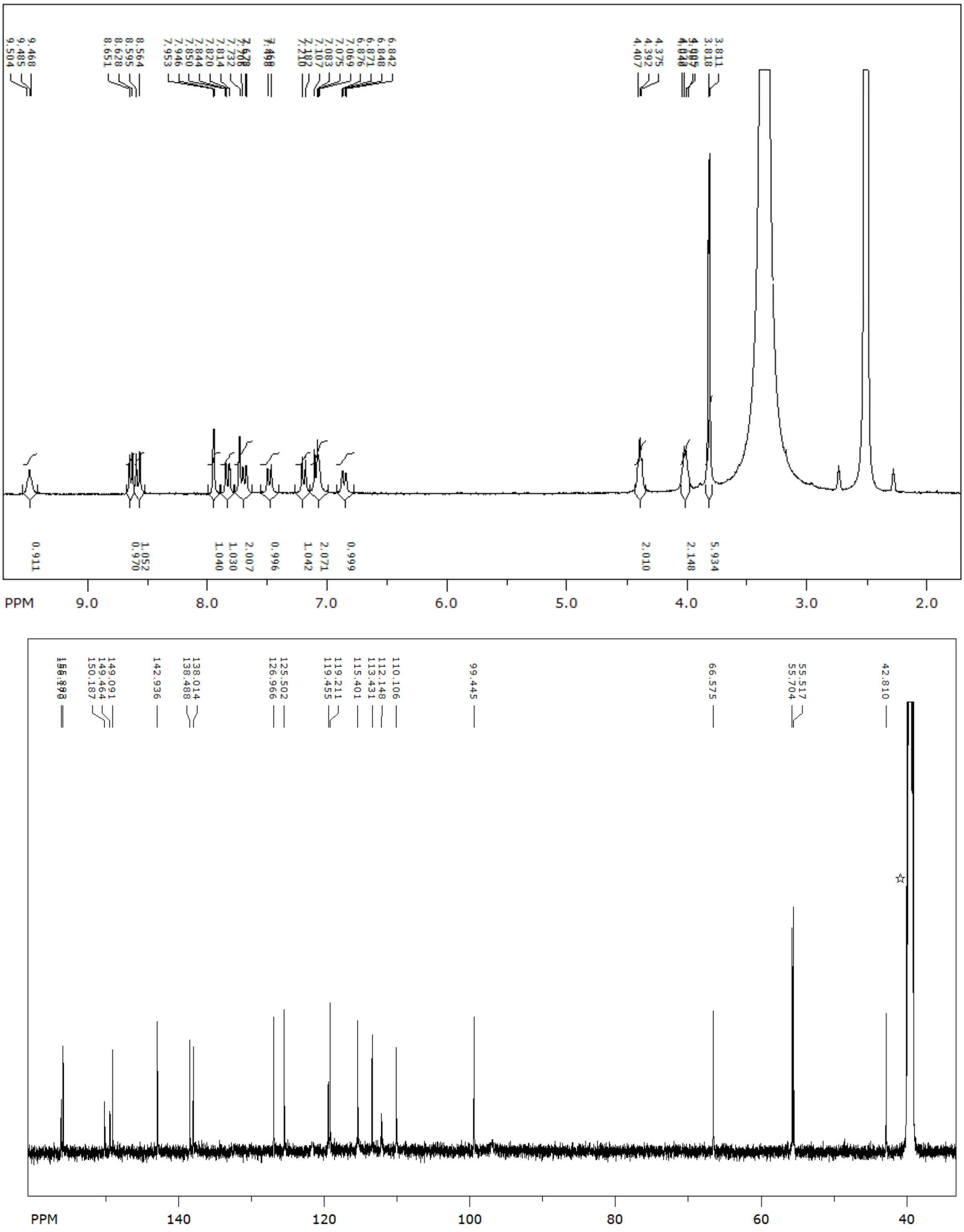

Figure S14 ^{1}H NMR and ^{13}C NMR of compound **12c** (DMSO; residual solvent signals)

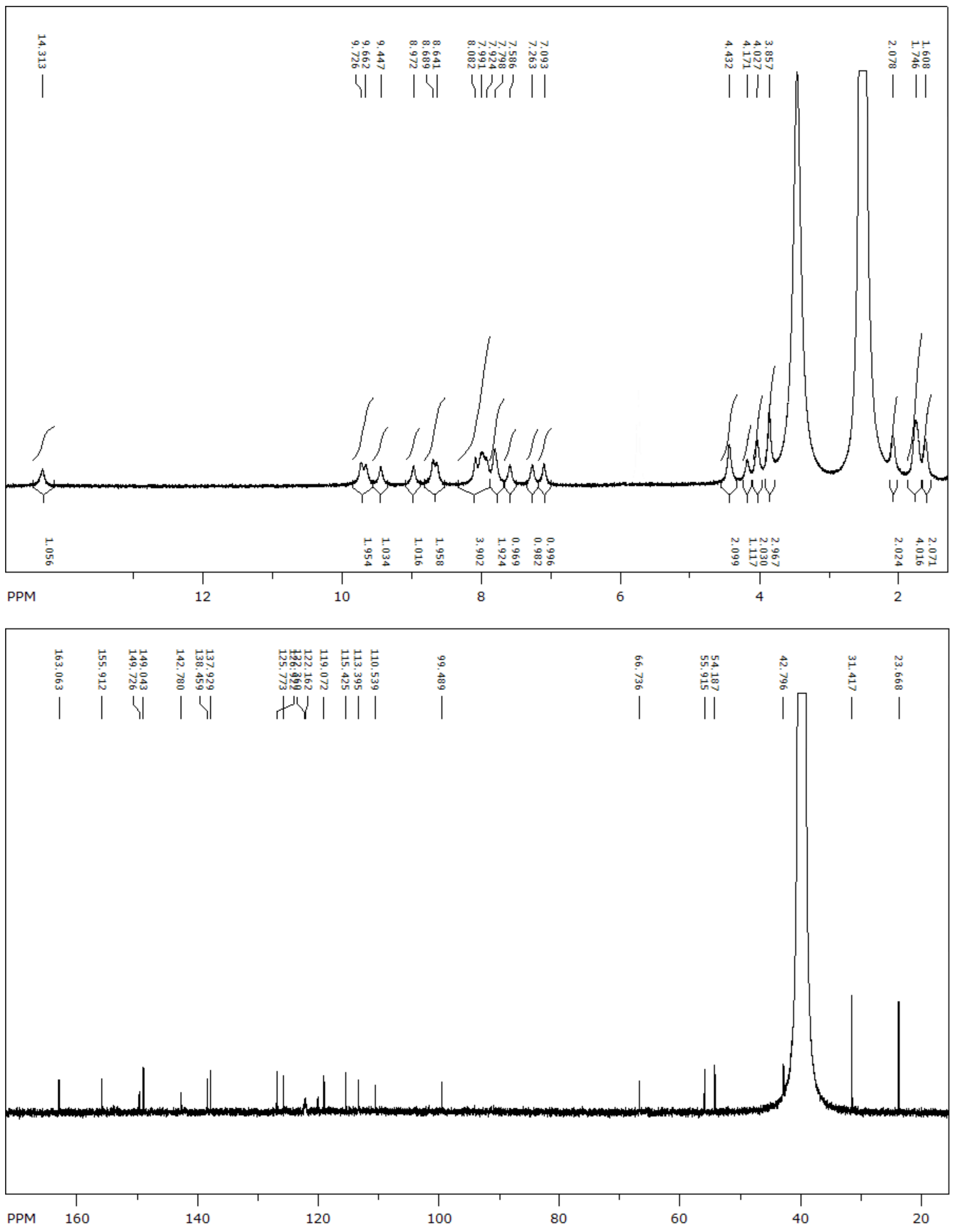

Figure S15 ^{1}H NMR and ^{13}C NMR of compound **12d**

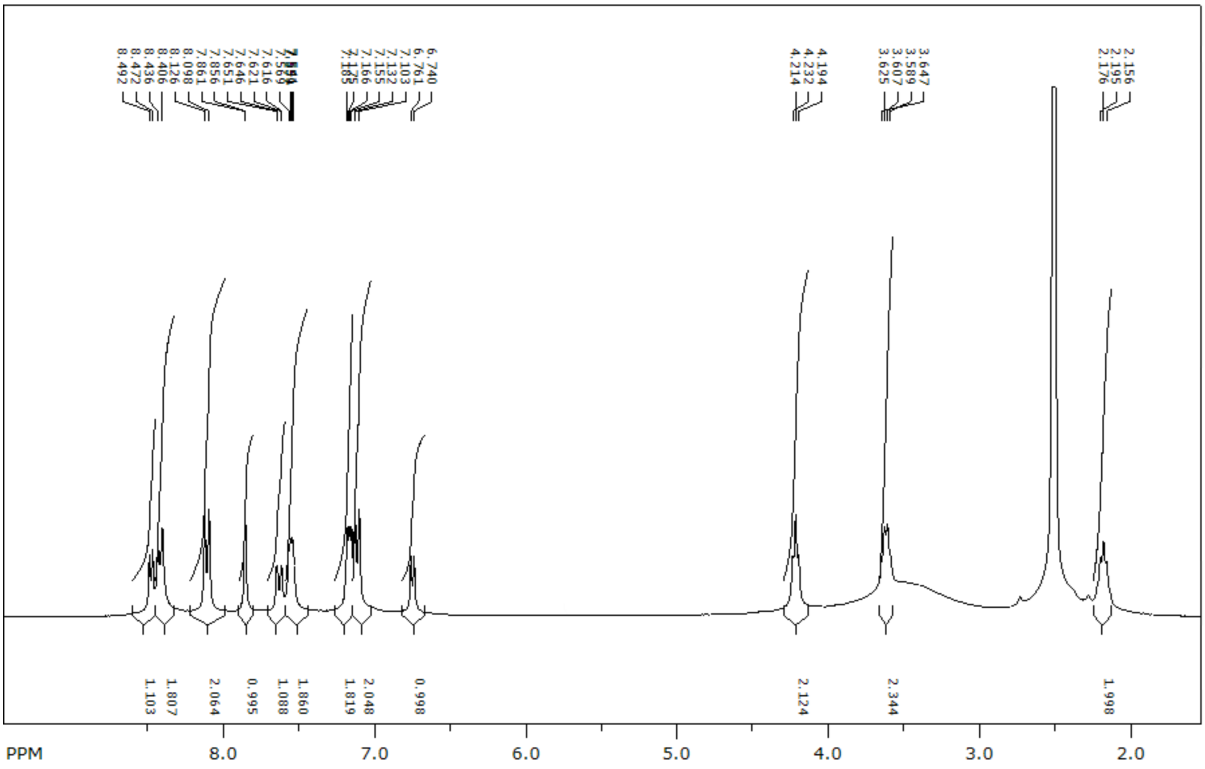

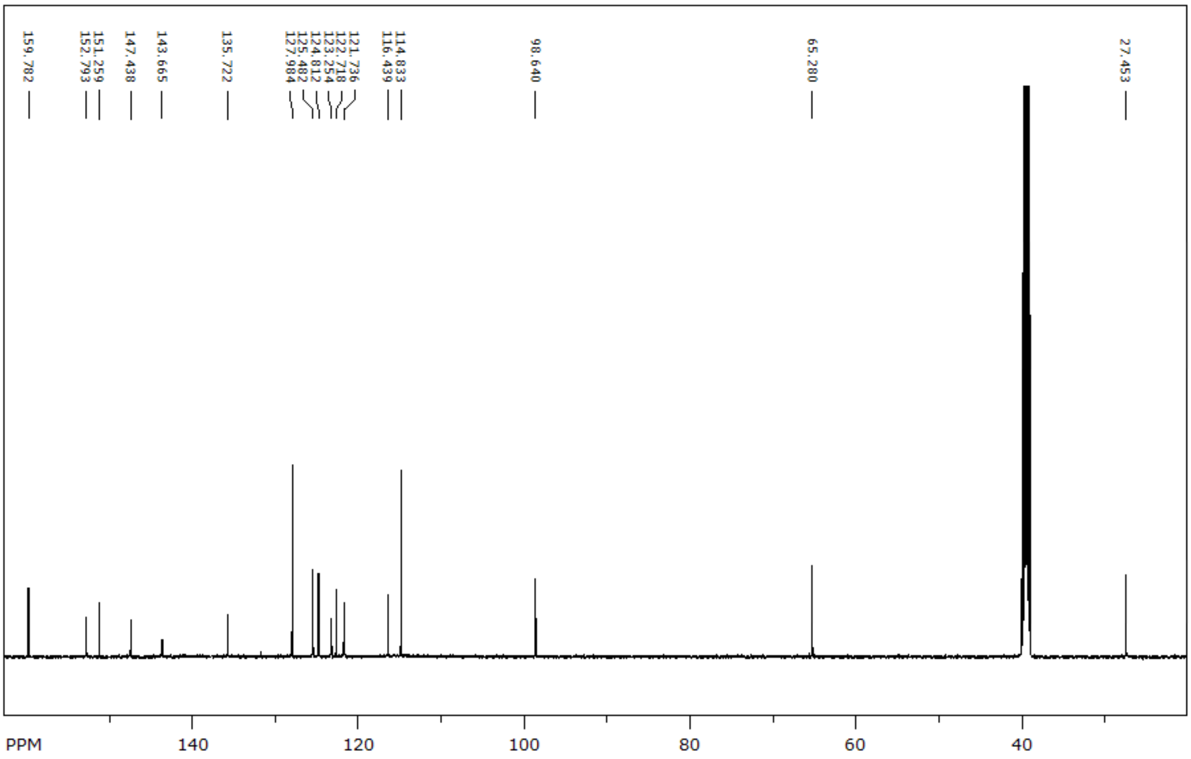

Figure S16 ^{1}H NMR and ^{13}C NMR of compound **13a**

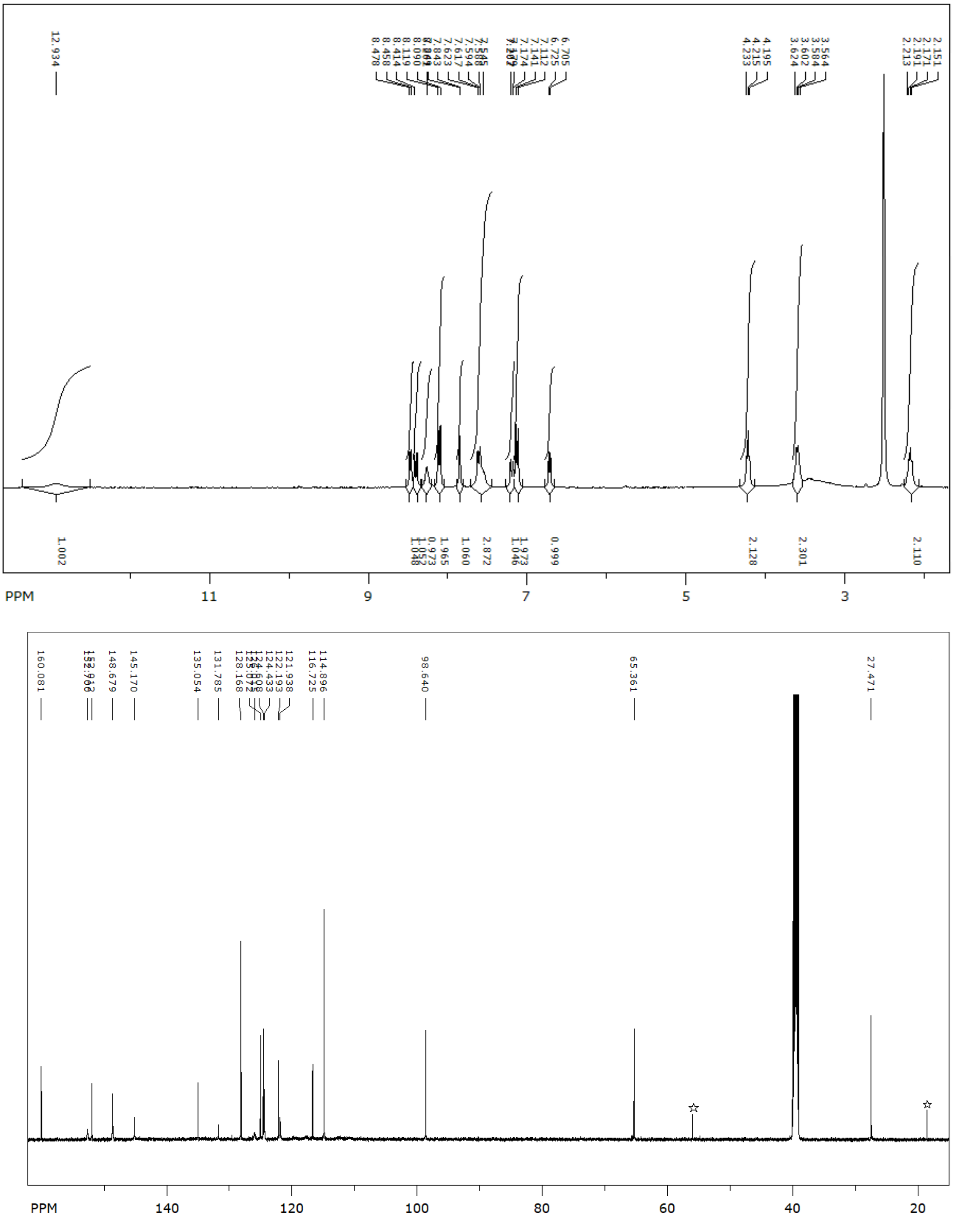

Figure S17 ^{1}H NMR and ^{13}C NMR of compound **13b** (*ethanol; residual solvent signal)

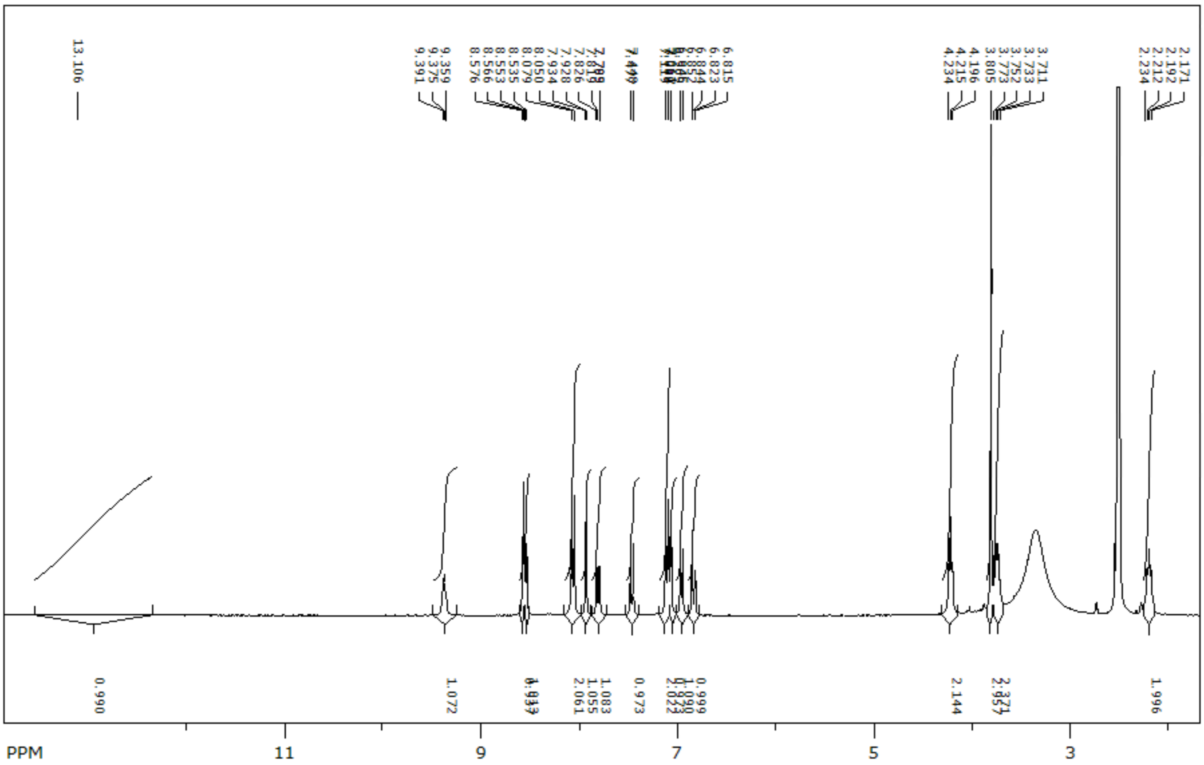

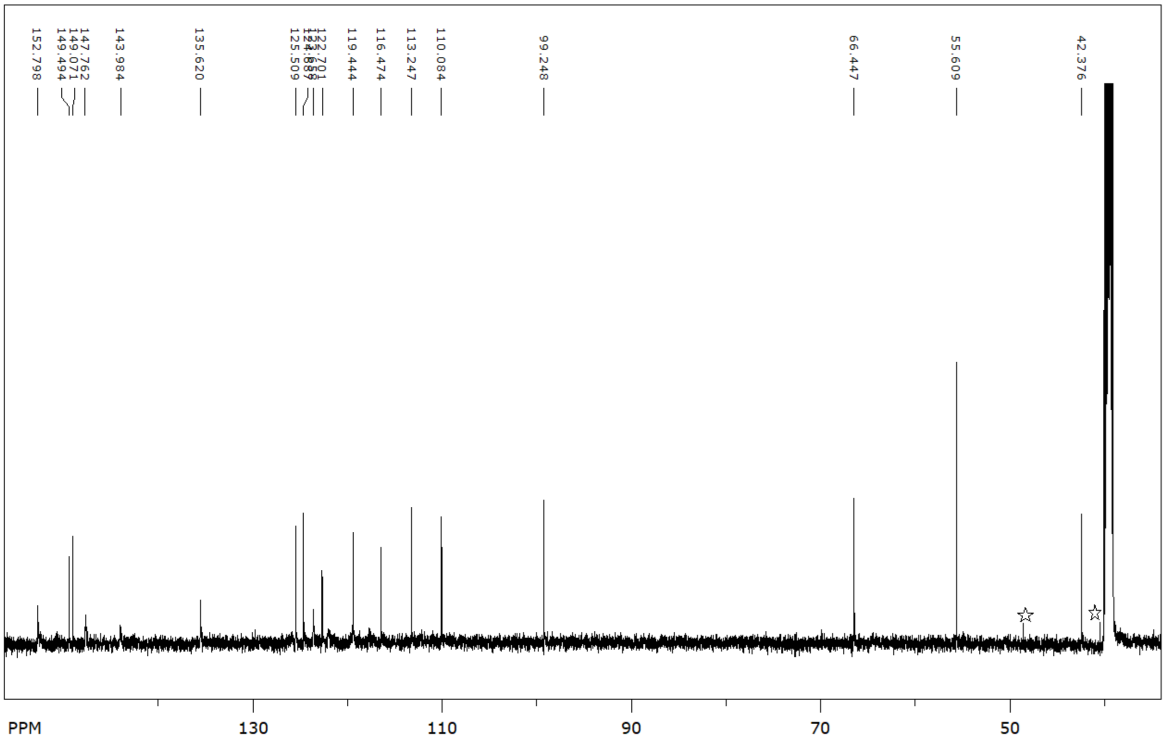

Figure S18 ^{1}H NMR and ^{13}C NMR of compound **13c** (*methanol, *DMSO; residual solvent signals)

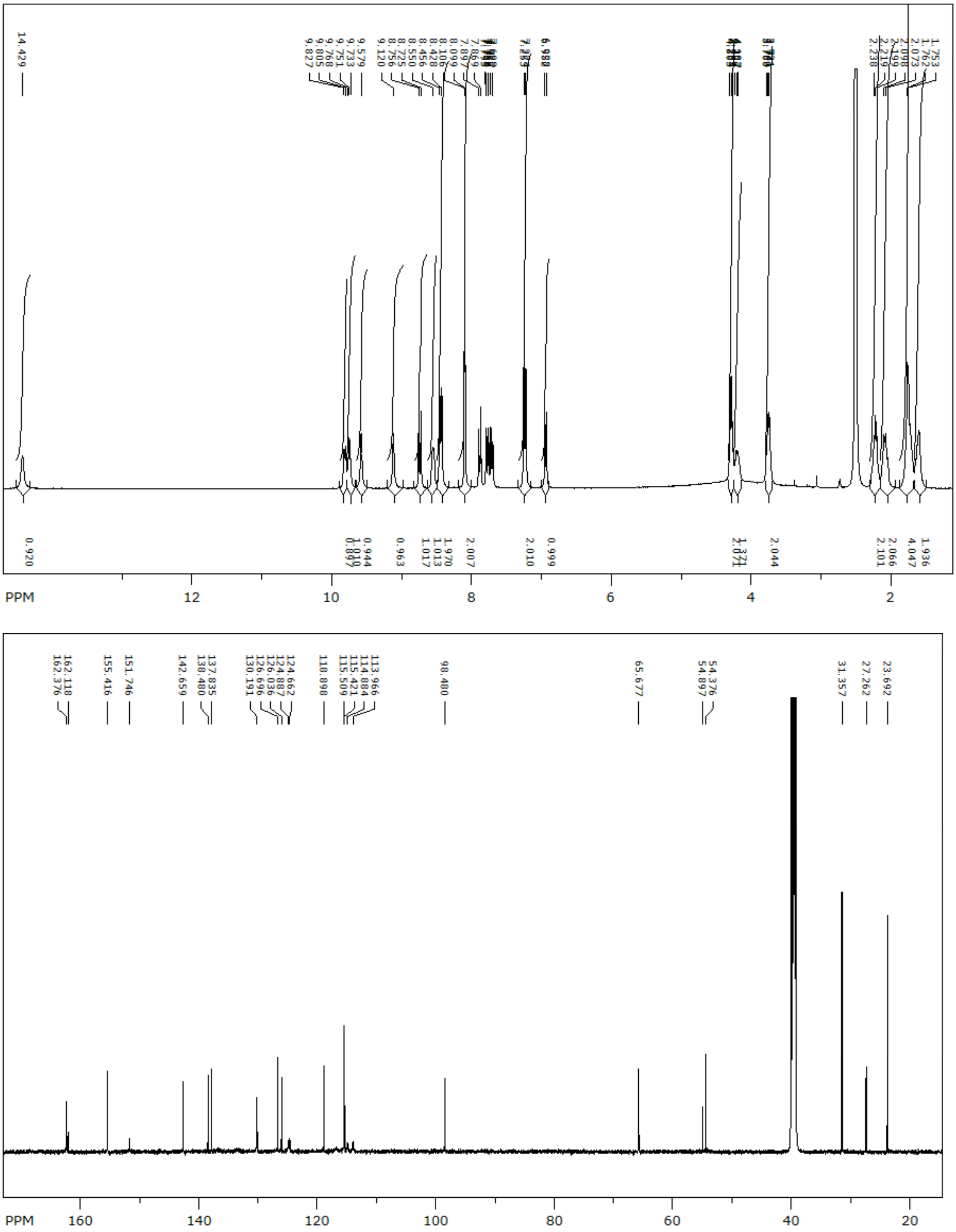

Figure S19 ^{1}H NMR and ^{13}C NMR of compound **13d**

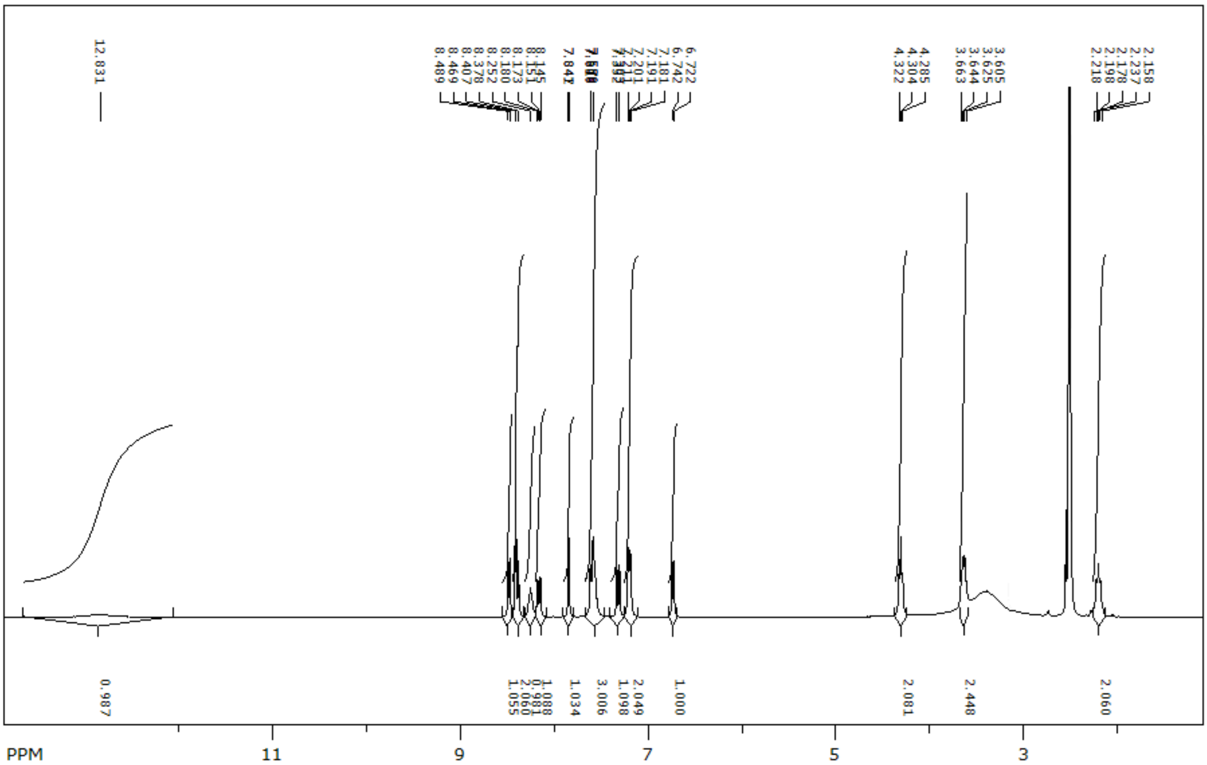

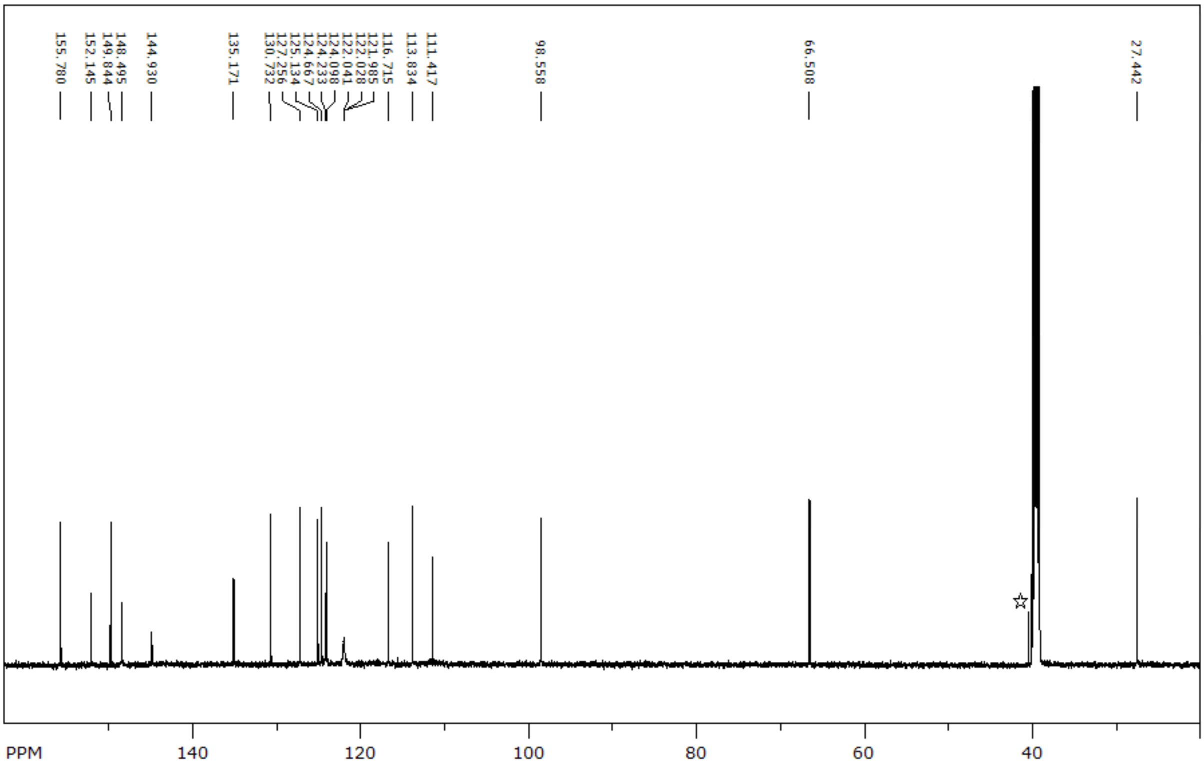

Figure S20 ^{1}H NMR and ^{13}C NMR of compound **14a** (*DMSO; residual solvent signal)

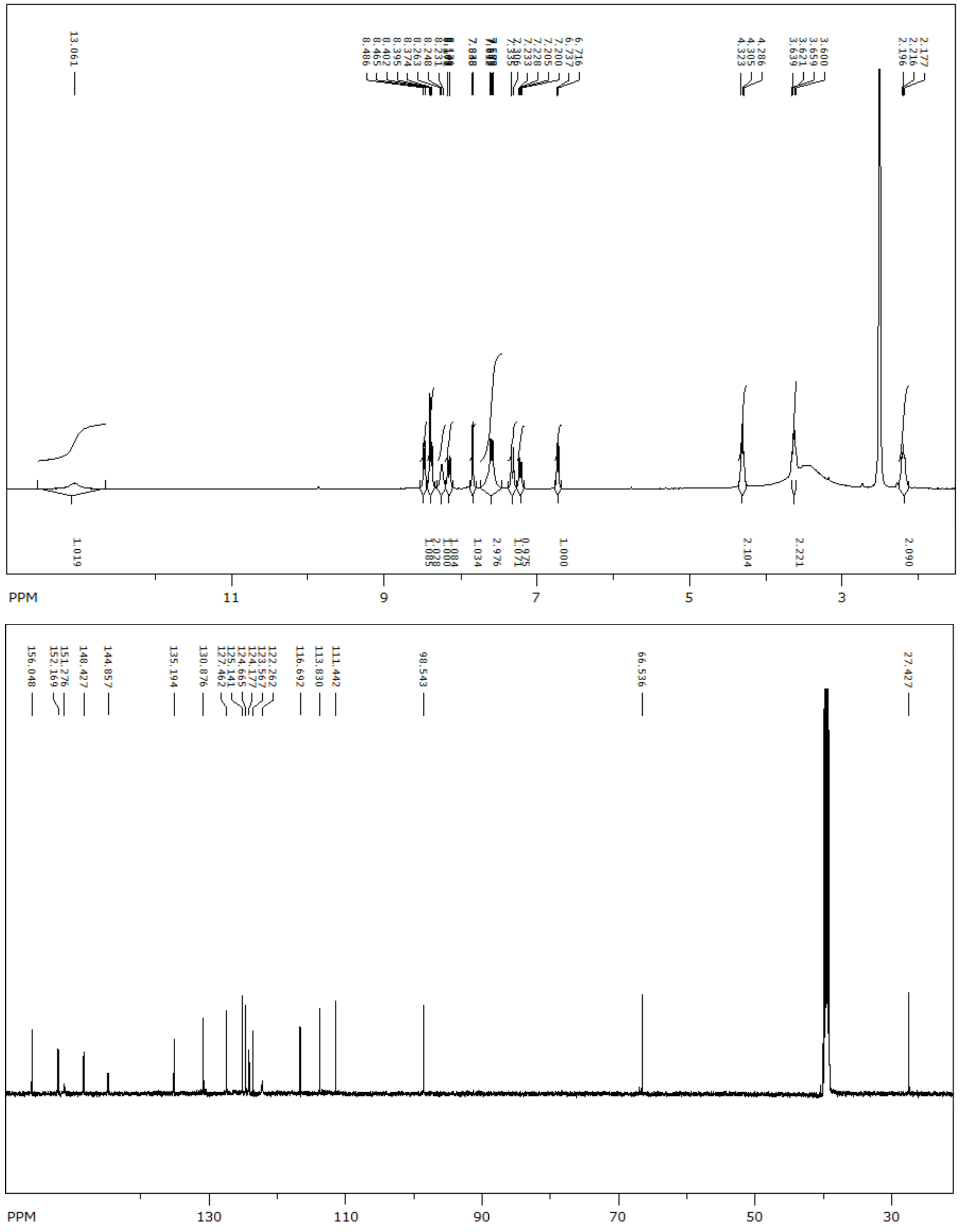

Figure S21 ^1H NMR and ^{13}C NMR of compound **14b**

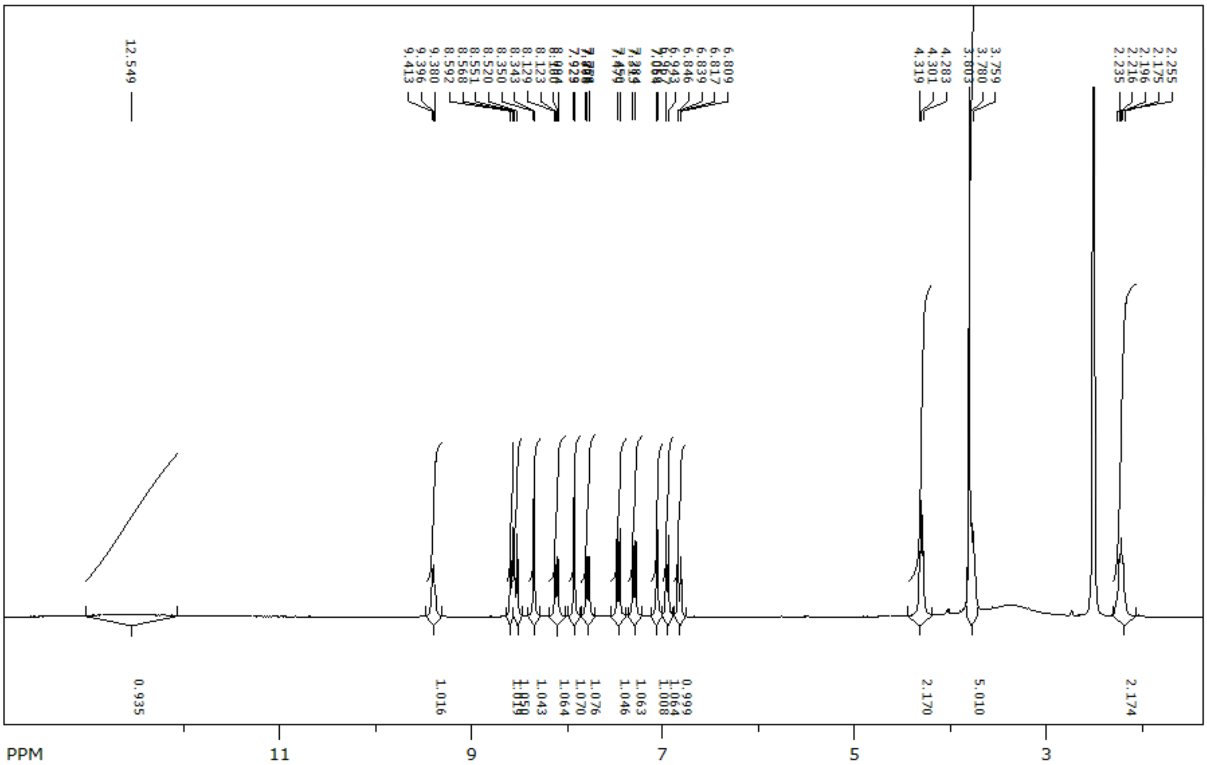

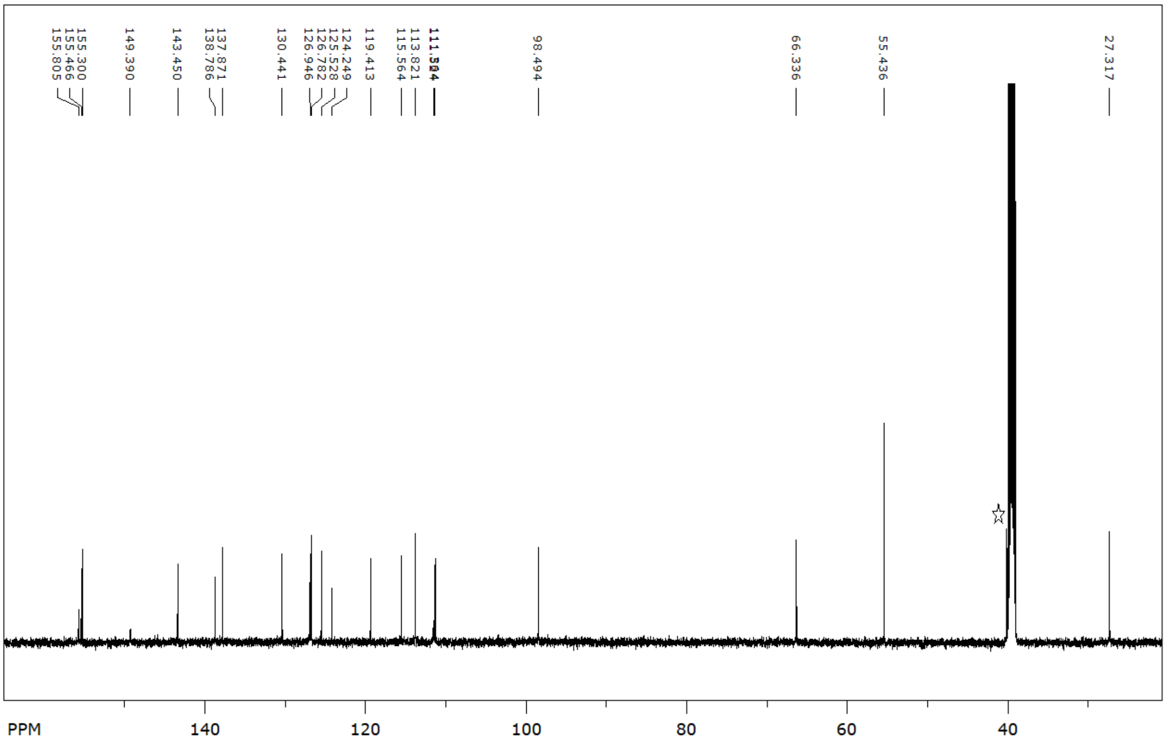

Figure S22 ^{1}H NMR and ^{13}C NMR of compound **14c** (*DMSO; residual solvent signal)

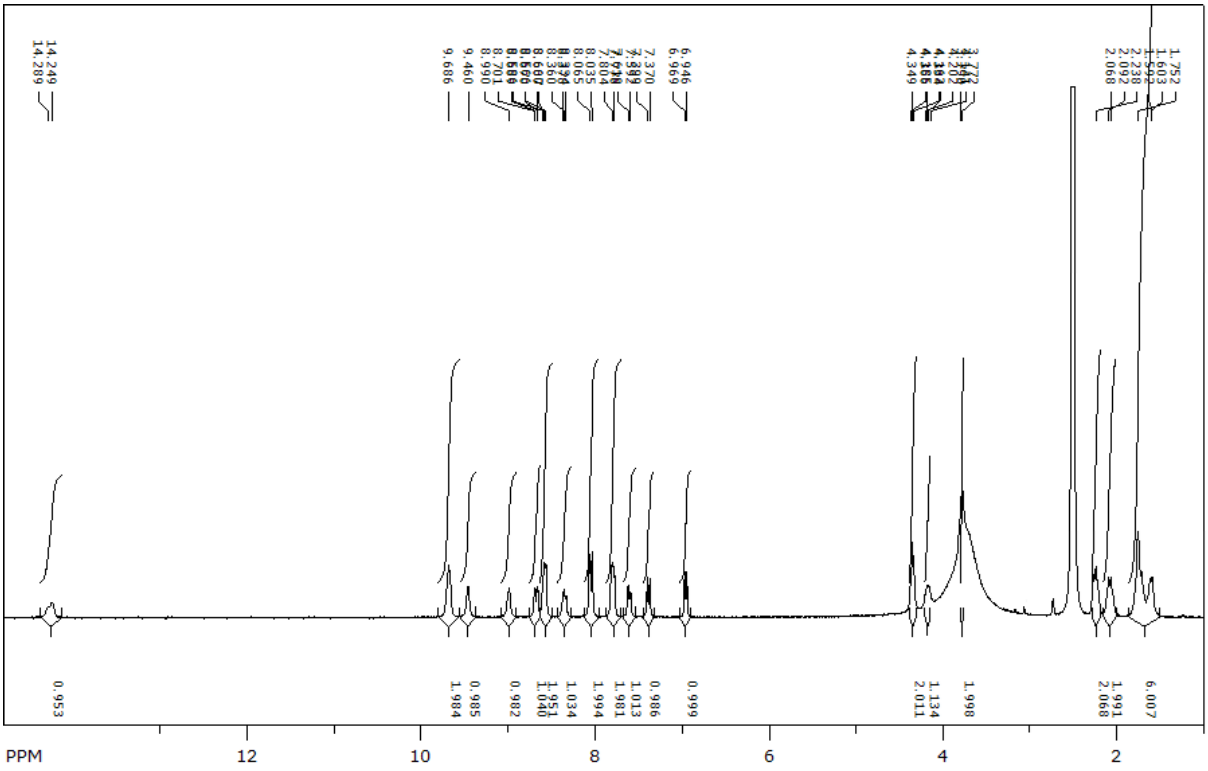

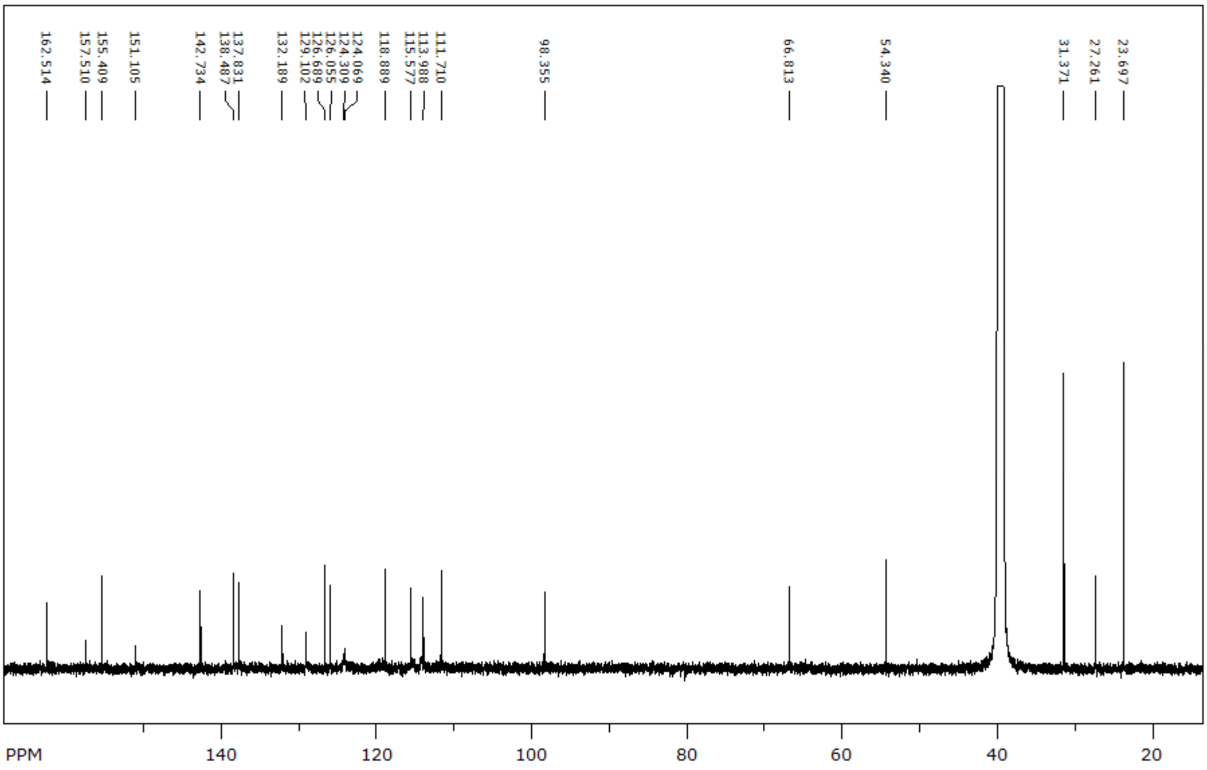

Figure S23 ^{1}H NMR and ^{13}C NMR of compound **14d**

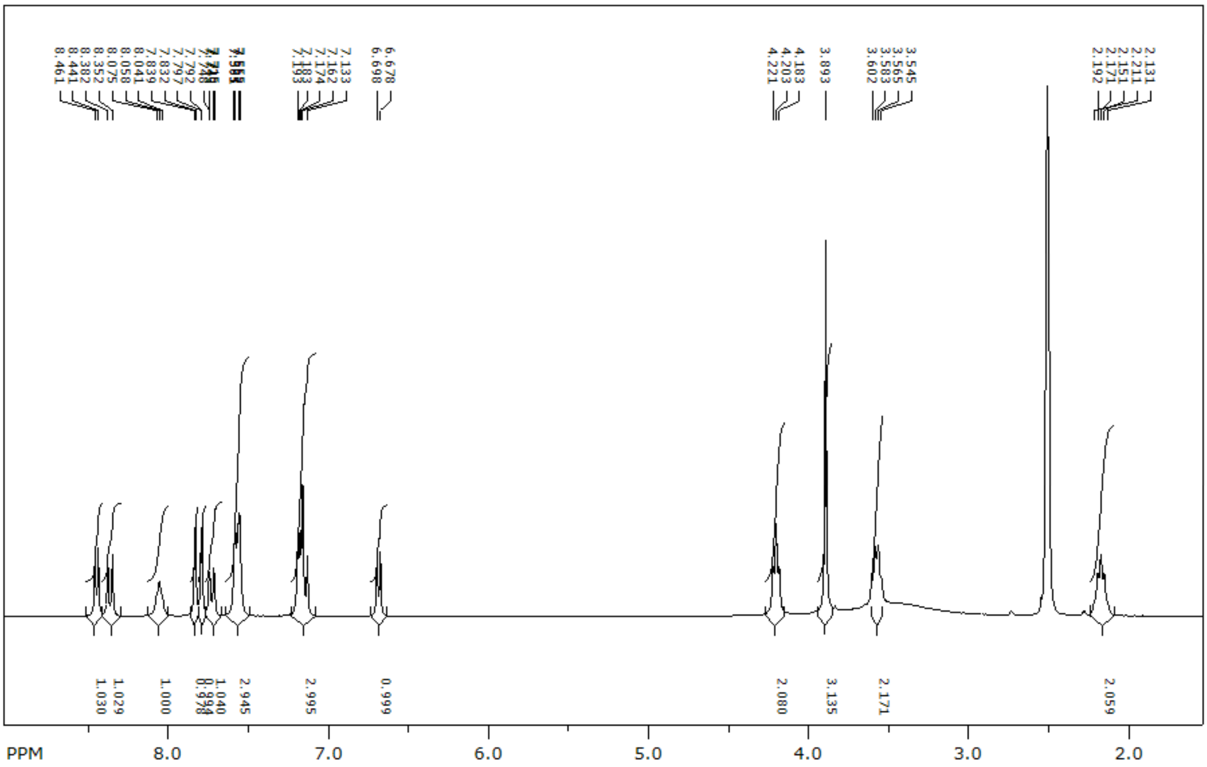

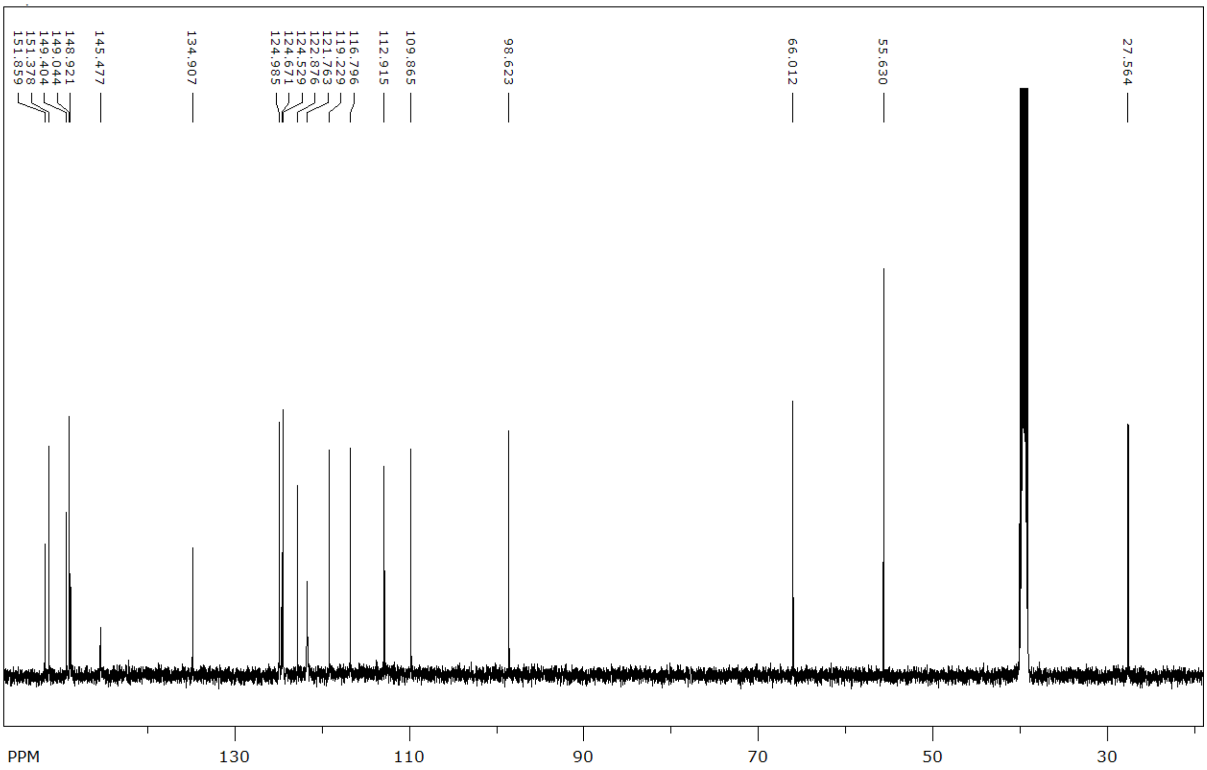

Figure S24 ^{1}H NMR and ^{13}C NMR of compound **15a**

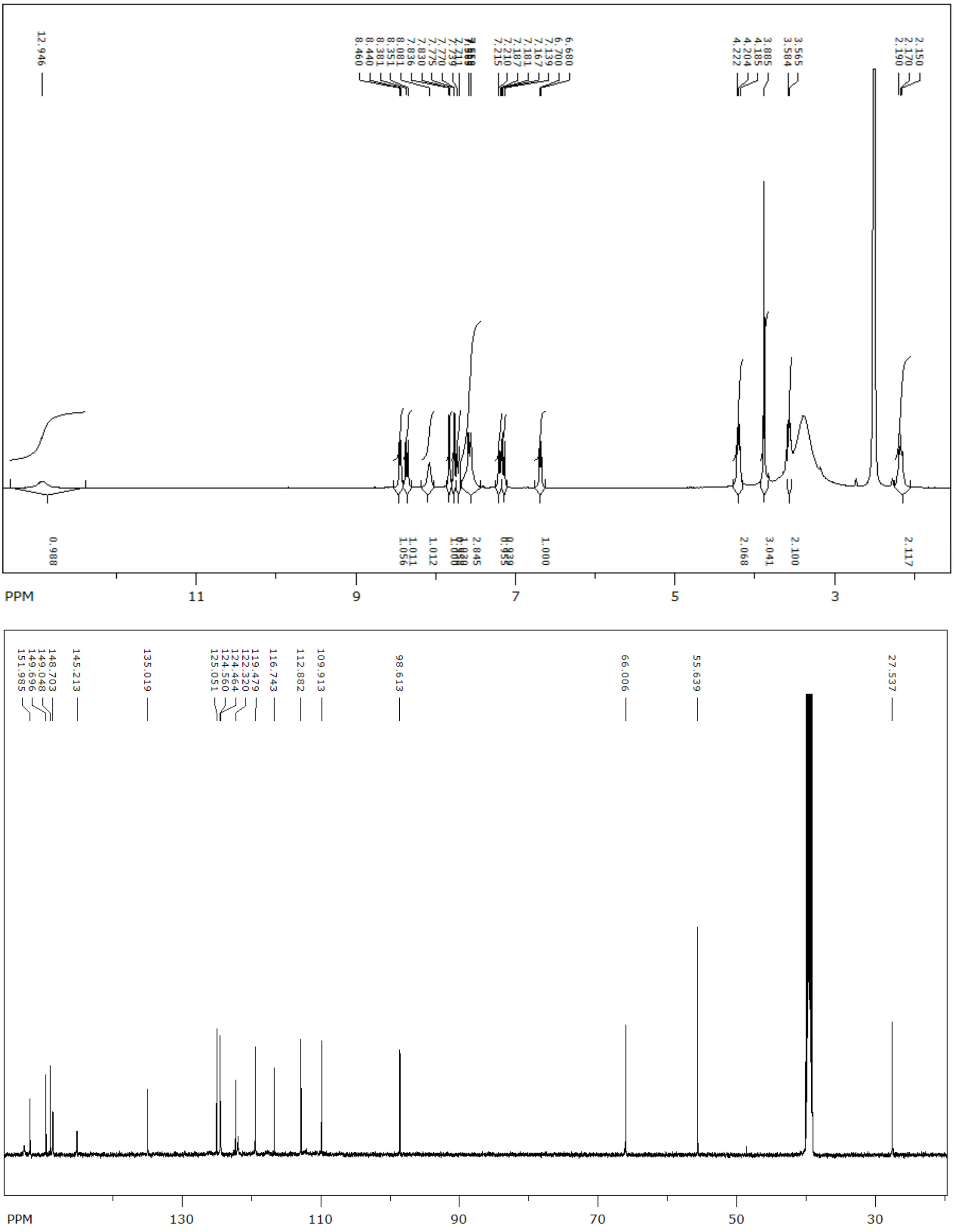

Figure S25 ^{1}H NMR and ^{13}C NMR of compound **15b**

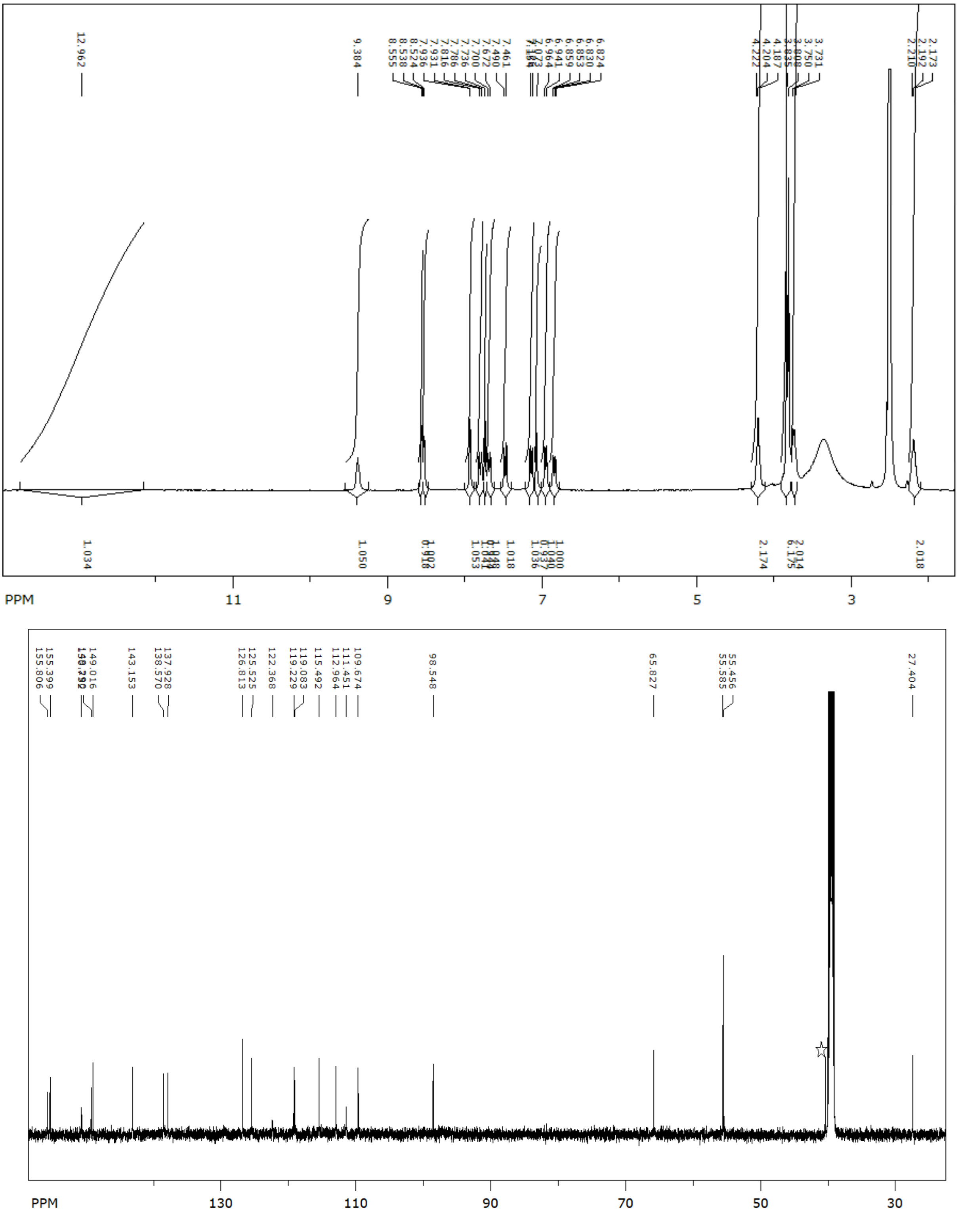

Figure S26 ^{1}H NMR and ^{13}C NMR of compound **15c** (*DMSO; residual solvent signal)

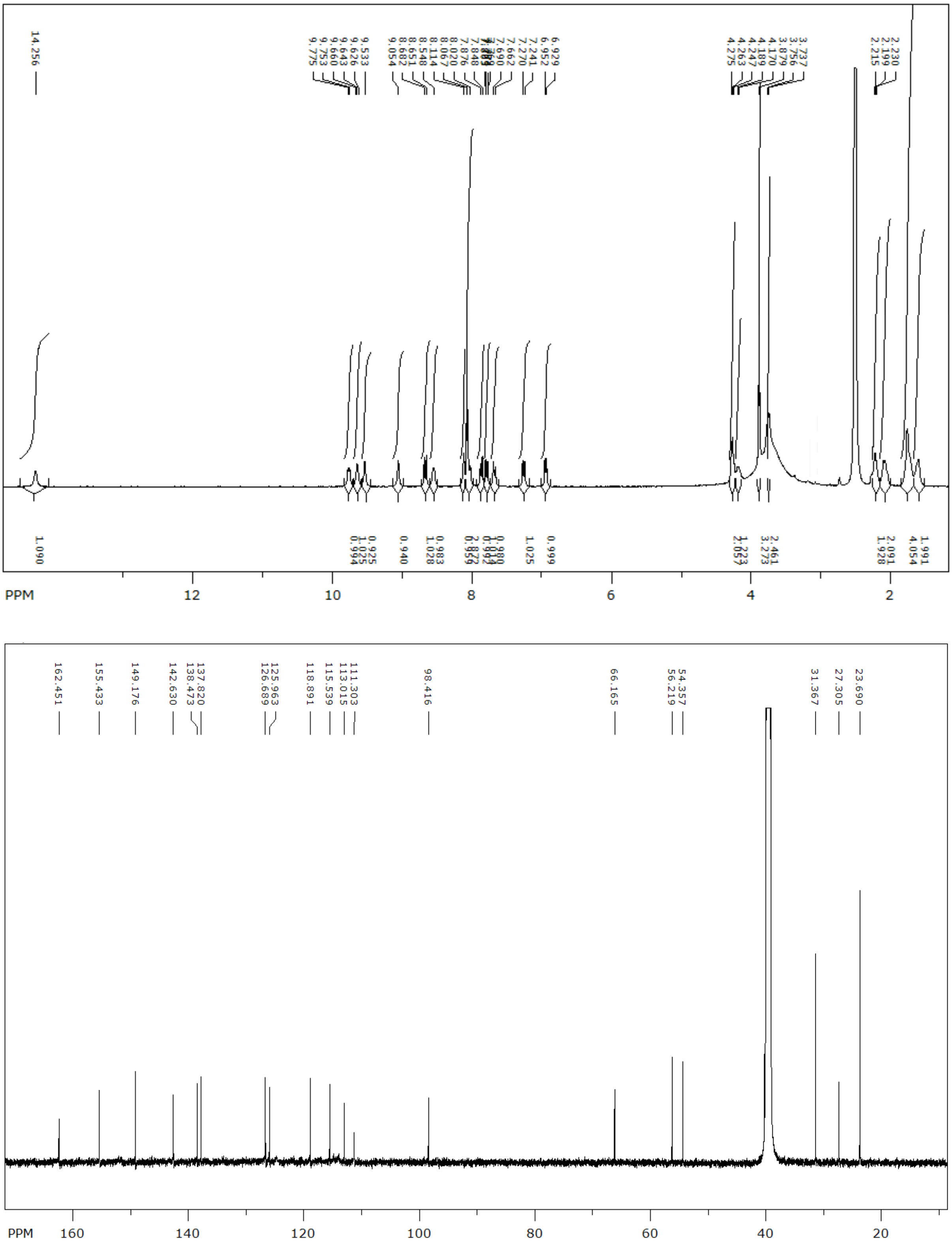

Figure S27 ^{1}H NMR and ^{13}C NMR of compound **15d**
